# Detection of 191 Taxifolin Metabolites and Their Distribution in Rats Using HPLC-ESI-IT-TOF-MS^n^

**DOI:** 10.3390/molecules21091209

**Published:** 2016-09-13

**Authors:** Ping Yang, Feng Xu, Hong-Fu Li, Yi Wang, Feng-Chun Li, Ming-Ying Shang, Guang-Xue Liu, Xuan Wang, Shao-Qing Cai

**Affiliations:** State Key Laboratory of Natural and Biomimetic Drugs, School of Pharmaceutical Sciences, Peking University Health Science Center, Beijing 100191, China; y.p.nn@163.com (P.Y.); lihongfu6688@163.com (H.-F.L.); wangyishaanxi@163.com (Y.W.); lcxbjmu@163.com (F.-C.L.); myshang@bjmu.edu.cn (M.-Y.S.); guangxl@bjmu.edu.cn (G.-X.L.); xuanwang6818@bjmu.edu.cn (X.W.)

**Keywords:** taxifolin, metabolites, HPLC-ESI-IT-TOF-MS^n^, in vivo, additive effect at the same target

## Abstract

Taxifolin is a ubiquitous bioactive constituent of foods and herbs. To thoroughly explore its metabolism in vivo, an HPLC-ESI-IT-TOF-MS^n^ method combined with specific metabolite detection strategy was used to detect and identify the metabolites of taxifolin in rats. Of the 191 metabolites tentatively identified, 154 were new metabolites, 69 were new compounds and 32 were dimers. This is the first report of the in vivo biotransformation of a single compound into more than 100 metabolites. Furthermore, acetylamination and pyroglutamic acid conjugation were identified as new metabolic reactions. Seventeen metabolites were found to have various taxifolin-related bioactivities. The potential targets of taxifolin and 63 metabolites were predicted using PharmMapper, with results showing that more than 60 metabolites have the same five targets. Metabolites with the same fragment pattern may have the same pharmacophore. Thus these metabolites may exert the same pharmacological effects as taxifolin through an additive effect on the same drug targets. This observation indicates that taxifolin is bioactive not only in the parent form, but also through its metabolites. These findings enhance understanding of the metabolism and effective forms of taxifolin and may provide further insight of the beneficial effects of taxifolin and its derivatives.

## 1. Introduction

Taxifolin (dihydroquercetin) is a bioactive flavanonol commonly found in grapes [1], citrus fruits [2], onions [2,3], green tea [1], olive oil [2], wine [1], and many other foods [2], as well as several herbs (such as milk thistle [4], French maritime bark [5], Douglas fir bark [6], and Smilacis Glabrae Rhizoma [7]). It is also widely used as a food additive and can be found in health supplement products such as silymarin (Legalon™), Pycnogenol^®^ and Venoruton^®^ [8].

As a ubiquitous constituent of foods and herbs, taxifolin is consumed regularly in the human diet and exerts a wide range of biochemical and pharmacological effects; these include antioxidant [9,10], antitumor [11] and anti-inflammatory effects [12], the prevention of Alzheimer’s disease [13], antidiabetic [14,15], antiviral [16], antimicrobial [17], hepatoprotective [18], cardioprotective [15,19], neuroprotective [20] and immunoregulatory effects [21], and xanthine oxidase inhibition [22]. Additionally, experimental data indicate that taxifolin use is safe and nontoxic [2,23].

It has been reported that the effective forms of flavonoids are not necessarily the natural phytochemical forms, but the metabolites [24,25,26] arising from them in vivo. It is well established that conjugation reactions with glucuronic acid, sulphuric acid, and their mixtures are the most common type of metabolic pathways for flavonoids [27,28]. Some studies have shown that phase II metabolites possess certain pharmacological activities such as anti-inflammatory, antioxidant and antitumor effects, and can interact with metabolic enzymes and transporters [26,27,28].

Like other flavonoids, taxifolin can be metabolized, absorbed, and circulated in conjugate form throughout the body, thus exerting beneficial effects in target tissues [29,30,31]. According to our previous studies, a single bioactive constituent of herbs can produce more than 50 [32] or 80 [33] metabolites in vivo. However, until now, only about 27 in vitro and in vivo metabolites of taxifolin have been described. The predominant metabolites include 3,4-dihydroxyphenylacetic acid [23,34], phloroglucinol [34], *m*-hydroxyphenylacetic acid [23], 3-methoxy-4-hydroxylphenylacetic acid [23], a dehydroxylation metabolite [35], methylation product [30,35], sulphate [35], glucuronide [35], methylated glucuronides [35], a diastereomer [30], methylation isomer [30] and dehydration metabolites [30]. Accordingly, the biotransformation of taxifolin and the biological activities of its metabolites still need further investigation.

The apparent permeability of taxifolin across Caco-2 cell monolayers (a widely used in vitro model of the human small intestinal mucosa) was shown to be less than 1 × 10^−6^ cm/s [36], and the absolute bioavailability of taxifolin was reported as 0.17% in rats [37]. The bioavailability of taxifolin was 36% in rabbits upon detection of total conjugated and free taxifolin in plasma following enzymatic hydrolysis [38]. The question therefore remains as to how taxifolin exerts its biochemical and pharmacological effects with such low bioavailability. Previous findings indicate that the parent compound of taxifolin is found at low levels in the blood, and that conjugates represent the main forms in vivo. Moreover, the urinary excretion of taxifolin was found to be only 0.24% of the dosage [30]. Therefore, we believe that taxifolin may be easily metabolized and that its metabolites are the prevalent form in vivo, although limited information is available on metabolism of taxifolin in vivo. To gain a more comprehensive understanding of taxifolin metabolism and its effective forms [39], mechanisms of action, and pharmacological effects in vivo, it is necessary to thoroughly profile its metabolites and determine their distribution. Accordingly, we used high-performance liquid chromatography with electrospray ionization ion trap time-of-flight multistage mass spectrometry (HPLC-ESI-IT-TOF-MS^n^) combined with a specific metabolite analysis strategy to profile and identify the metabolites of taxifolin and study their distribution in rats.

## 2. Results and Discussion

### 2.1. Identification of Taxifolin in Rats and Study on the Fragmentation Behaviours of Taxifolin and Reference Compounds

Taxifolin ([M − H]^−^ at *m/z* 303.0510, molecular formula C_15_H_12_O_7_) was unambiguously identified in rat plasma, urine, faeces and eight organ samples by comparing its retention time (t_R_ = 41.023 min) and MS^n^ data with the reference compound. To facilitate the identification of metabolites, the fragmentation characteristics of taxifolin in the negative ion mode (NI) were observed and analysed (Supplemental Table S1 and Figures S1 and S2). The characteristic fragment ions of taxifolin in NI were *m/z* 285.0407 ([M − H − H_2_O]^−^), *m/z* 241.0524 ([M − H − H_2_O − CO_2_]^−^), *m/z* 177.0253 (^1,4^B^−^ − 2H), *m/z* 175.0424 ([M − H − H_2_O − C_3_O_2_ − C_2_H_2_O]^−^) and *m/z* 125.0290 (^1,4^A^−^ + 2H) in its MS^2^ spectrum. Quercetin (C_15_H_10_O_7_) showed characteristic fragment ions at *m/z* 229.0526, *m/z* 211.0386, *m/z* 179.0015, *m/z* 151.0061 and *m/z* 107.0230 in its MS^2^ spectrum. Dihydrokaempferol (C_15_H_12_O_6_) showed characteristic fragment ions at *m/z* 269.0431, *m/z* 259.0613, *m/z* 243.0663, *m/z* 201.0564, *m/z* 173.0622 and *m/z* 125.0275 in its MS^2^ spectrum.

### 2.2. Identification of 191 Metabolites of Taxifolin in Rats

**Figure 1:**
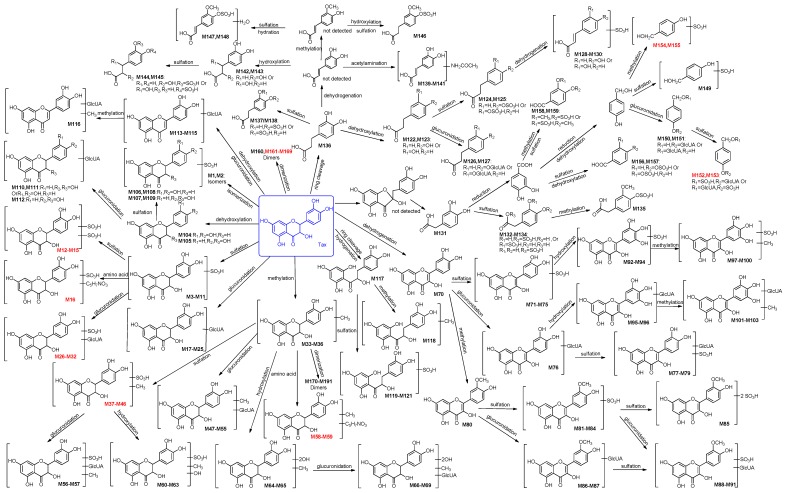
The proposed metabolic pathways of taxifolin in rats, with **M1**–**M191** metabolites. The blue is taxifolin, the red shows new compound.

Metabolites of taxifolin in rats were identified on the basis of knowledge of taxifolin metabolism and the strategy proposed in our previous study [39]. The metabolic reactions were identified according to characteristic neutral losses. Compared with the parent compound, the formation of metabolites with mass shifts of +15.99 Da (O), −15.99 Da (O), +14.01 Da (CH_2_), −2.01 Da (H_2_), +2.01 Da (H_2_), −18.01 Da (H_2_O), +18.01 Da (H_2_O), +79.95 Da (SO_3_) and +176.03 Da (C_6_H_8_O_6_) indicated hydroxylation, dehydroxylation, methylation, dehydrogenation, hydrogenation, dehydration, hydration, sulphation, and glucuronidation, respectively. The molecular formulae were predicted based on HRMS data, and the specific type and structure of metabolites were identified by the fragmentation characteristics in their NI MS^n^ spectra. In total, 191 metabolites (including 127 metabolites in urine, 83 metabolites in plasma, 43 metabolites in faeces and 46 metabolites in eight organs) of taxifolin were tentatively identified (Table 1) by careful MS^n^ data analysis, and their existence was further confirmed by comparing the corresponding extracted ion chromatograms (EICs) of drug and blank groups. The detailed LC-MS data are summarized in Table 1 and Table S1, with potential metabolic pathways of taxifolin shown in Figure 1. Metabolic reactions are summarized in Table 2. Among the 191 metabolites, 154 were new metabolites of taxifolin, and 69 metabolites were new compounds that could not be found in the SciFinder database, including 12 taxifolin conjugates, 22 methyl taxifolin derivatives, four phenolic acid derivatives, and 31 dimers. The 191 metabolites were divided into eight categories: 32 metabolites having the aglycone of taxifolin or its isomers, 37 metabolites having the aglycone of methyl taxifolin, 34 metabolites having the aglycone of quercetin, nine metabolites having the aglycone of dehydroxylated taxifolin, four metabolites formed through dehydration and glucuronidation, five metabolites having the aglycone of hydrogenated taxifolin, 38 metabolites having the aglycone of phenolic acid derivatives and 32 metabolites formed through dimerization.

#### 2.2.1. Identification of 32 Metabolites (**M1**–**M32**) Having the Aglycone of Taxifolin or Its Isomers

In total, 32 metabolites (12 new compounds) having the aglycone of taxifolin or its isomers were identified in the drug group, including two isomers and 30 conjugates of taxifolin or its isomers. Isomerization metabolites **M1** and **M2** had the same molecular formula and similar fragmentation behaviour as taxifolin. Because C-2 and C-3 are chiral centres, taxifolin has four stereoisomers [7], therefore, these metabolites were tentatively identified as stereoisomers of taxifolin. As for the taxifolin conjugates **M3**–**M32**, in the NI MS^2^ spectra of **M3**–**M32**, the same [aglycone − H]^−^ (*m/z* 303.05) was observed, with identical molecular formula and fragmentation behaviour to taxifolin. We therefore deduced that the metabolites were conjugates of taxifolin. According to their characteristic neutral losses, **M3**–**M11** were identified as sulphates of taxifolin or its isomers. Conjugates **M12**–**M15** were identified as taxifolin disulphates. The molecular formula of **M16** was determined to be C_20_H_19_NO_13_S from its HRMS data. The base peak ion at *m/z* 383.0083 was formed by neutral loss of 129.05 Da (C_5_H_7_NO_3_). According to our previous research [40], we deduced that the metabolic reaction was amino acid conjugation and the lost fragment C_5_H_7_NO_3_ was speculated to be pyroglutamic acid (the most referenced compound having the molecular formula of C_5_H_7_NO_3_) based on the SciFinder academic database. Hence, **M16** was tentatively identified as taxifolin sulphate and pyroglutamic acid conjugate. **M17**–**M25** were identified as glucuronides of taxifolin or its isomers, and **M26**–**M32** were identified as taxifolin glucuronide sulphates.

#### 2.2.2. Identification of 37 Metabolites (**M33**–**M69**) Having the Aglycone of Methyl Taxifolin

In total, 37 metabolites (22 new compounds) having the aglycone of methyl taxifolin or its isomer were found in the drug group, including four methyltaxifolin isomers, 23 conjugates of methyl taxifolin or its isomers, four conjugates of methyl and hydroxylated taxifolin, and six conjugates of methyl and dihydroxylated taxifolin.

The molecular formulae of **M33**–**M36** were calculated to be C_16_H_14_O_7_, which is 14.01 Da (CH_2_) more than that of taxifolin. Hence, these compounds were identified as methylated taxifolin. Generally, the hydroxyl group at the C-5 position is not readily metabolized [40]. Therefore, the sites of methylation were found to be the hydroxyl groups of the C-3, 7, 3′ and 4′ positions of taxifolin.

Based on the ClogP rule (the smaller the ClogP value, the smaller the retention time value) [39], and considering that the main in vivo methylation metabolite of taxifolin is 3′*-O-*methyltaxifolin [30], **M33** (t_R_ = 50.292, the relative peak area was the largest) was tentatively identified as 3′*-O-*methyl-taxifolin (ClogP = 1.21715); **M34** (t_R_ = 51.350) as 4′*-O-*methyl-taxifolin (ClogP = 1.21715); **M35** (t_R_ = 52.875) as 7*-O-*methyl-taxifolin (ClogP = 1.29372); and **M36** (t_R_ = 53.592) as 3*-O-*methyl-taxifolin (ClogP = 1.40805).

In NI MS^2^ spectra of **M37**–**M59**, the same [aglycone − H]^−^ (*m/z* 317.06) was observed with identical molecular formula and fragmentation behaviours to methyl taxifolin. We therefore deduced that they were conjugates of methyl taxifolin. **M37**–**M46** were methyl taxifolin sulphates; **M47**–**M55** were glucuronides of methyl taxifolin; **M56**–**M57** were methyl taxifolin glucuronide sulphates and **M58**–**M59** were identified as methyl taxifolin pyroglutamic acid conjugates similar to **M16**.

As for metabolites **M60**–**M63** formed through methylation, hydroxylation and sulphation, the neutral loss of 79.95 Da (SO_3_) was observed in the MS^2^ spectra of **M60**–**M63** and the aglycone had the molecular formula of C_16_H_14_O_8_, one more oxygen atom (mass shifts of +15.99) than that of methyl taxifolin. We therefore deduced that these metabolites were sulphates of hydroxylated methyl taxifolin.

**M64**–**M65** showed [M − H]^−^ at m/z 349.06. Their molecular formulae were calculated to be C_16_H_14_O_9_, 31.98 Da (2O) more than that of methyl taxifolin and resulting in their temporary identification as methylated and dihydroxyled taxifolin. **M66**–**M69** showed [M − H]^−^ at m/z 525.09 and then yielded [aglycone − H]^−^ at m/z 349.06 by neutral loss of 176.03 Da; the aglycones were identical to **M64**–**M65**. Hence, these metabolites were tentatively identified as glucuronides of methylated and dihydroxylated taxifolin.

#### 2.2.3. Identification of 34 Metabolites (**M70–M103**) Having the Aglycone of Quercetin

In total, 34 metabolites having the aglycone of quercetin were found from the drug group, including quercetin, isorhamnetin, nine quercetin conjugates, 11 isorhamnetin conjugates and 12 conjugates of hydroxylated quercetin.

Metabolite **M70** was formed through dehydrogenation. The [M − H]^−^ of **M70** was at *m/z* 301.0349 (C_15_H_9_O_7_), which is 2.01 Da (H_2_) less than taxifolin, and the retention time and characteristic fragment ions were the same as those for the reference compound quercetin. **M70** was thus determined to be quercetin.

In the NI MS^2^ spectra of **M71**–**M79**, the same [aglycone − H]^−^ (*m/z* 301.04) was observed with identical molecular formula and fragmentation behaviour as quercetin. We therefore deduced that they were conjugates of quercetin. Based on characteristic neutral losses, **M71**–**M75** were identified as quercetin sulphates. According to the ClogP rule, **M71** (t_R_ = 51.583) was quercetin-5*-O-*sulphate (ClogP = −0.897894), **M72** (t_R_ = 52.647) was quercetin-7-*O*-sulphate (ClogP = 0.00210607), **M73** (t_R_ = 56.3, relative peak area = 378222) and **M74** (t_R_ = 57.033, relative peak area = 3335213) were quercetin-3′/4′*-O-*sulphate (ClogP = 0.0554161) and **M75** (t_R_ = 58.173) was quercetin-3*-O-*sulphate (ClogP = 0.160939). According to the literature [41], the favoured sulphation sites of quercetin are 3′ and 7-OH. The relative peak area of **M74** was higher than that of **M73**, indicating that **M74** was quercetin-3′*-O-*sulphate and **M73** was quercetin-4′*-O-*sulphate. **M76** was identified as quercetin glucuronide and **M77**–**M79** were identified as quercetin glucuronide sulphates.

The molecular formula of **M80** was calculated as C_16_H_12_O_7_, 14.01 Da (CH_2_) more than quercetin. Given that 3′-OH is the main methylation site of quercetin according to the literature [41], **M80** was identified as 3′*-O-*methyl-quercetin (isorhamnetin). In the NI MS^2^ spectra of **M81**–**M91**, the same [aglycone − H]^−^ (*m/z* 315.05) was observed with identical molecular formula and fragmentation behaviour to isorhamnetin. Hence, these metabolites were considered as conjugates of isorhamnetin. **M81**–**M84** were isorhamnetin sulphates. Based on the ClogP rule, **M81** (t_R_= 48.633) was isorhamnetin-5-*O*-sulphate (ClogP = −0.452683), **M82** (t_R_ = 56.917) was isorhamnetin-7-*O*-sulphate (ClogP = 0.447317), **M83** (t_R_ = 58.042) was isorhamnetin-3-*O*-sulphate (ClogP = 0.605693) and **M84** (t_R_ = 58.922) was the isorhamnetin-4′-*O*-sulphate (ClogP = 0.631748). **M85** was identified as isorhamnetin disulphate and **M86**–**M87** were identified as glucuronides of isorhamnetin. According to the literature [41], **M86**–**M87** was tentatively identified as isorhamnetin-4′/7-*O*-glucuronide. Based on the ClogP rule, **M86** (t_R_ = 49.212) was isorhamnetin-4′-*O*-glucuronide (ClogP = −0.133551) and **M87** (t_R_ = 50.428) was isorhamnetin-7-*O*-glucuronide (ClogP = 0.0320181). **M88**–**M91** were identified as isorhamnetin glucuronide sulphates.

In the NI MS^2^ spectra of **M92**–**M96**, the same aglycone (C_15_H_10_O_8_), 15.99 Da (O) more than quercetin, was observed; hence, they were identified as hydroxylated quercetins. In addition, we can deduce that they were conjugates of hydroxyquercerin. According to characteristic neutral losses, **M92**–**M94** were identified as sulphates of hydroxylated quercetin. **M95**–**M96** were glucuronides of hydroxylated quercetin.

In the NI MS^2^ spectra of **M97**–**M103**, the same aglycone (C_16_H_12_O_8_), 15.99 Da (O) more than isorhamnetin, was observed, hence, it was identified as hydroxylated isorhamnetin. Furthermore, we deduced that these metabolites were conjugates of hydroxylated isorhamnetin. **M97**–**M100** were tentatively identified as sulphates of hydroxylated isorhamnetin and **M101**–**M103** were glucuronides of hydroxylated isorhamnetin.

#### 2.2.4. Identification of 9 Metabolites (**M104–M112**) Having the Aglycone of Dehydroxylated Taxifolin

In total, nine metabolites including two dehydroxylated taxifolins, and seven conjugates of dehydroxylated taxifolin or isomers were identified.

The molecular formulae of **M104** and **M105** were calculated to be C_15_H_12_O_6_ and they were identified as dehydroxylated taxifolin when compared with taxifolin. The fragment ions at *m/z* 137.0222 (^0,2^B^−^) in the MS^2^ spectrum of **M104** indicated that there were two hydroxyl groups linked to the B-ring, and that the A ring might have two hydroxyl groups based on *m/z* 107.0174 (^0,4^A^−^) and *m/z* 165.0205 (^1,2^A^−^). Therefore, **M104** was tentatively identified as eriodictyol. The characteristic fragment ions of **M105** at *m/z* 269.0368 ([M − H − H_2_O]^−^), *m/z* 259.0621 ([M − H − CO]^−^), *m/z* 243.0647 ([M − H − CO_2_]^−^), *m/z* 201.0554 ([M − H − CO_2_ − C_2_H_2_O]^−^), *m/z* 173.0683([M − H − CO − CO_2_ − C_2_H_2_O]^−^) and *m/z* 125.0290 (^1,4^A^−^ + 2H) were consistent with the reference compound dihydrokaempferol. Hence, **M105** was identified as dihydrokaempferol.

In the NI MS^2^ spectra of **M106**–**M112**, the same [aglycone − H]^−^ (*m/z* 287.05) with identical molecular formula and fragmentation behaviour to dehydroxylated taxifolin was observed, we therefore deduced that these were conjugates of dehydroxylated taxifolin. The characteristic fragment ions of the [aglycone − H]^−^ of **M106** and **M108** were the same as those of eriodictyol. Because the main sulphation sites were located at C-3′ and C-7, and based on the ClogP rule, **M106** (t_R_ = 37.325) was tentatively identified as eriodictyol-7-*O*-sulphate (ClogP = 0.224621) and **M108** (t_R_= 37.708) as eriodictyol-3′-*O*-sulphate (ClogP = 0.398051). The characteristic fragment ions of the [aglycone − H]^−^ of **M107** and **M109** were identical to those of dihydrokaempferol. Hence, **M107** (t_R_ = 38.200) was dihydrokaempferol 7-*O*-sulphate (ClogP = −0.255279) and **M109** (t_R_ = 40.383) was dihydrokaempferol 4′-*O*-sulphate (ClogP = −0.192048). **M110**–**M112** yielded [aglycone − H]^−^ by neutral loss of 176.03 Da (C_6_H_8_O_6_), which suggested that **M110**–**M112** were glucuronides of dehydroxylated taxifolin. The characteristic fragment ions of **M112** were consistent with dihydrokaempferol, so **M112** was considered to be dihydrokaempferol glucuronide.

#### 2.2.5. Identification of Four Metabolites (**M113–M116**) Formed through Dehydration and Glucuronidation

Four metabolites were identified, including three luteolin glucuronides and one methyl luteolin glucuronide. **M113**–**M115** showed [M + NH_3_ − H]^−^ at *m/z* 478.10 (predicted to be C_21_H_20_O_12_N) in their HRMS data. The [aglycone + NH_3_ − H]^−^ was formed by the neutral loss of 176.03 Da in the NI MS^2^ spectra and the aglycone had the molecular formula of C_15_H_10_O_6_, which is 18.01 Da (H_2_O) less than taxifolin (C_15_H_12_O_7_). The characteristic fragment ions of the aglycone were *m/z* 217.06 ([M − H − C_3_O_2_]^−^), *m/z* 175.03 ([M − H − C_3_O_2_ − C_2_H_2_O]^−^) and *m/z* 177.03 (^0^^,4^B^−^), indicating that there were two hydroxyl groups linked to the A-ring and B-ring, respectively. Accordingly, the aglycone was considered as the dehydration metabolite of taxifolin and tentatively identified as luteolin. As a result, **M113**–**M115** were glucuronides of luteolin. Because C-5 was not easily conjugated, the sites of glucuronidation were considered to be the hydroxyl groups of the C-7, 3′ and 4′ positions of luteolin. Based on the ClogP rule, **M113** (t_R_ = 16.017) was luteolin-7-*O*-glucuronide (ClogP = 0.335925), and **M114** (t_R_ = 16.583) and **M115** (t_R_ = 17.483) were luteolin-3′/4′-*O*-glucuronide (ClogP = 0.188342). **M116** showed [M + NH_3_ − H]^−^ at *m/z* 492.1165 (predicted to be C_22_H_23_O_12_N) in the HRMS data. In the MS^2^ spectrum, the neutral loss of 176.03 Da (C_6_H_8_O_6_) was observed and the aglycone was 14.01 Da (CH_2_) more than luteolin. Hence, the aglycone was methyl luteolin, and **M116** was identified as the glucuronide of methyl luteolin.

#### 2.2.6. Identification of Five Metabolites (**M117–M121**) Having the Aglycone of Hydrogenated Taxifolin

In total, five metabolites including hydrogenated taxifolin, hydrogenated methyltaxifolin and three hydrogenated taxifolin sulphates were detected. **M117** showed [M − H]^−^ at *m/z* 305.0652, which was 2.01 Da (H_2_) more than taxifolin, and the characteristic fragment ions were at *m/z* 287.0565 (C_15_H_11_O_6_), *m/z* 183.0309 (C_8_H_7_O_5_), *m/z* 165.0249 (C_8_H_5_O_4_), *m/z* 161.0287 (C_9_H_5_O_3_) and *m/z* 137.0301 (C_7_H_5_O_3_). Therefore, **M117** was tentatively identified as a hydrogenated product. The molecular formula of **M118** was calculated to be C_16_H_16_O_7_, which is 2.01 Da (H_2_) more than that of methyltaxifolin; hence, **M118** was identified as hydrogenated methyl taxifolin. **M119**–**M121** yielded [aglycone − H]^−^ at *m/z* 305.06 by neutral loss of 79.95 Da (SO_3_), indicating that they were hydrogenated taxifolin sulphates.

#### 2.2.7. Identification of 38 Metabolites (**M122–M159**) Having the Aglycone of Phenolic Acid Derivatives

In total, 38 metabolites (four new compounds) having the aglycone of phenolic acid derivatives were found in the drug group, including phenolic acids and their conjugations.

Metabolites having the aglycone of hydroxyphenylpropanoic acid: **M122**–**M130**. The [M − H]^−^ of **M122**–**M123** were at *m/z* 165.06, and characteristic fragment ions at *m/z* 121.07 and *m/z* 119.04 were observed in their MS^2^ spectra. According to a previous report [42], we identified **M122**–**M123** as 3/4-hydroxyphenylpropanoic acid. **M124**–**M127** yielded [aglycone − H]^−^ at *m/z* 165.06 by neutral loss of 79.95 Da or 176.03 Da. Hence, **M124**–**M125** were identified as sulphates of hydroxyphenylpropanoic acid. **M126**–**M127** were glucuronides of hydroxyphenylpropanoic acid. **M128**–**M130** yielded [aglycone − H]^−^ at *m/z* 163.04 (C_9_H_8_O_3_) by the loss of SO_3_ (79.96Da) and produced characteristic fragment ions at *m/z* 163.04 (100.0) and *m/z* 119.06 (17.51). According to a previous report [39], we identified **M128**–**M130** as *p*/*m*-coumaric acid sulphates.

Metabolites having the aglycone of dihydroxyphenylacetic acid: **M131**–**M135**. **M131** showed [M − H]^−^ at *m/z* 167.0349 (predicted to be C_8_H_7_O_4_), and characteristic fragment ions at *m/z* 123.0458 were observed in NI MS^2^ spectrum. According to a previous report [43], we identified **M131** as dihydroxyphenylacetic acid, a known metabolite of taxifolin. **M132**–**M134** yielded [aglycone − H]^−^ at *m/z* 167.04 by neutral loss of 79.95 Da, indicating that these were sulphates of dihydroxyphenylacetic acid. **M135** showed [M − H]^−^ at *m/z* 261.0073 and yielded [aglycone − H]^−^ at *m/z* 181.0569 by neutral loss of 79.95Da (SO_3_) with characteristic fragment ions at *m/z* 217.0189 ([M − H − CO_2_]^−^), 181.0569 ([M − H − SO_3_]^−^), 137.0659 ([M − H − SO_3_ − CO_2_]^−^) and 123.0520 ([M − H − SO_3_ − CO_2_ − CH_2_]^−^). According to the previous report [44], **M135** was tentatively identified as homovanillic acid sulphate.

Metabolites having the aglycone of dihydrocaffeic acid: **M136**–**M138**. **M136** showed [M − H]^−^ at *m/z* 181.0504 (predicted to be C_9_H_9_O_4_) and characteristic fragment ion at *m/z* 137.0642 ([M − H − CO_2_]^−^) was observed in the NI MS^2^ spectra. According to a previous report [45], we identified **M136** as dihydrocaffeic acid. **M137**–**M138** yielded [aglycone − H]^−^ at *m/z* 181.05 by neutral loss of 79.95 Da and were tentatively identified as dihydrocaffeic acid sulphate.

Metabolites having the aglycone of caffeic acid: **M139**–**M145**. **M139**–**M141** showed [M − H]^−^ at *m/z* 238.07 (predicted to be C_11_H_12_NO_5_) and yielded [aglycone − H]^−^ at *m/z* 179.04 in the MS^2^ spectra by neutral loss of 59.03 Da (C_2_H_5_NO). The aglycone had the same molecular formula and characteristic fragment ions as caffeic acid. Therefore, **M139**–**M141** were designated caffeic acid acetyl amination metabolites. **M142**–**M143** showed [M − H]^−^ at *m/z* 197.05, which is 18.01 Da (H_2_O) more than caffeic acid; thus, they were tentatively identified as hydration metabolites of caffeic acid. Based on the ClogP rule, **M142** (t_R_ = 11.692) was 3-(3,4-dihydroxyphenyl)-3-hydroxypropanoic acid (ClogP = −0.6414) and **M143** (t_R_ = 12.658) was 3-(3,4-dihydroxyphenyl)-2-hydroxypropanoic acid (ClogP = −0.5798). **M144**–**M145** showed [M − H]^−^ at *m/z* 277.00, and the [aglycone − H]^−^ at *m/z* 197.05 was formed by the loss of 79.95 Da. Therefore, **M144**–**M145** were tentatively identified as the sulphates of caffeic acid hydrate.

Metabolites having the aglycone of ferulic acid: **M146**–**M148**. The molecular formula of **M146** was calculated to be C_10_H_12_O_7_S. The [aglycone − H]^−^ at *m/z* 195.0681 (C_10_H_11_O_4_) was formed by the loss of SO_3_ (79.95Da). Characteristic fragment ions at *m/z* 195.0681, 151.0845, 149.0632, 136.0607 and 119.0578 were observed in NI MS^2^ spectra. According to a previous report [45], we identified **M146** as dihydrogen ferulic acid sulphate. **M147**–**M148** showed [M − H]^−^ at *m/z* 291.02 and the [aglycone − H]^−^ at *m/z* 211.06 (C_10_H_11_O_5_) was formed by loss of SO_3_ (79.95 Da), which was 18.01 Da (H_2_O) more than ferulic acid (C_10_H_9_O_4_). Therefore, these metabolites were tentatively identified as the sulphates of ferulic acid hydrate.

In NI MS^2^ spectra of **M149**–**M155**, the same [aglycone − H]^−^ (*m/z* 123.05) was observed with a molecular formula identical to hydroxybenzyl alcohol. We therefore deduced that they were conjugates of hydroxybenzyl alcohol [46]. According to characteristic neutral losses, **M149** was tentatively identified as a sulphate of hydroxybenzyl alcohol, **M150–M151** were identified as glucuronides of hydroxybenzyl alcohol and **M152**–**M153** were identified as hydroxybenzyl alcohol glucuronide sulphates.

**M154**–**M155** showed [M − H]^−^ at *m/z* 217.02 (predicted to be C_8_H_9_O_5_S), and yielded [aglycone − H]^−^ at *m/z* 137.07 by neutral loss of 79.95 Da. The aglycone was 14.01 Da (CH_2_) more than hydroxybenzyl alcohol, so the compounds were tentatively identified as sulphates of methyl hydroxybenzyl alcohol.

Metabolites having the aglycone of hydroxybenzoic acid: **M156**–**M159**. **M156**–**M159** yielded [aglycone − H]^−^ by loss of 79.95 Da and so were sulphate conjugates. From the [aglycone − H]^−^ of **M156**–**M157** at *m/z* 137.03 (C_7_H_6_O_3_), these were identified as 3/4-hydroxybenzoic acid sulphates according to a previous report [39]. From the [aglycone − H]^−^ of **M158**–**M159** at *m/z* 167.04 (C_8_H_8_O_4_), they could identify as vanillic acid sulphate and isovanillic acid sulphate according to the previous report [39].

#### 2.2.8. Identification of 32 Metabolites (**M160–M191**) Formed through Dimerization

In total, 32 metabolites of dimerization (31 new compounds), including 10 taxifolin dimer derivatives and sulphates and 22 methyl taxifolin dimer derivatives and sulphates, were identified.

Dimers having the aglycone of taxifolin: **M160**–**M169**. The characteristic fragment ions of taxifolin at *m/z* 303.05, *m/z* 285.04 and *m/z* 241.05 were observed in the NI MS^2^ spectra of **M160**–**M169**. We therefore deduced that their structures contained taxifolin, and that they were taxifolin dimer derivatives. The molecular formula of **M160** was calculated to be C_31_H_24_O_13_ and, when compared with the molecular formula (C_15_H_12_O_7_) of taxifolin, we predicted that **M160** might be a dimer of taxiflolin and dehydroxylated methyl taxifolin. However, the site of dimerization was ambiguous. Only two forms of coupling bond are found between monomers of biflavonoids, namely C-C coupling and C-O coupling. In the NI MS^2^ spectra of **M160**, the relative abundance of *m/z* 303.0557 was less than 5% (4.08%), thus implying that the coupling bond between two monomers was extremely difficult to cleave [47]. Therefore, the dimer was considered to have formed through C-C coupling. One possible structure of **M160** and its fragmentation pathways are shown in Figure S3. Similar to **M160**, we predicted that **M161**–**M162** might be the dimers of taxiflolin and methyltaxifolin formed through C-C coupling. **M163** might be a dimer of taxiflolin and dimethyltaxifolin formed through C-C coupling. **M164**–**M166** were tentatively identified as sulphates of dimers of taxiflolin and methyltaxifolin. **M167**–**M169** were tentatively identified as sulphate of dimers of taxiflolin and dimethyltaxifolin.

Dimers having the aglycone of methyltaxifolin: **M170**–**M191**. The characteristic fragment ions of methyl taxifolin at *m/z* 317.06, *m/z* 299.05 and *m/z* 289.07 were observed in the NI MS^2^ spectra of **M170**–**M191** (except **M170**, **M172**, **M175**). Similar to **M160**, we predicted that **M170**–**M172** might be dimers of methyltaxiflolin and dehydroxylated methyltaxifolin. **M171** was identified as a dimer formed through C-O coupling. **M173**–**M176** might be dimers of methylquercetin and methyl-taxifolin. Among these, **M174** was identified as a dimer formed through C-C coupling while **M173**, **M175** and **M176** were identified as dimers formed through C-O coupling. **M177**–**M179** might be dimers of methyl taxiflolin and dehydroxylated dimethyltaxifolin formed through C-O coupling. **M180** and **M181** might be dimers of methyltaxiflolin and methyltaxifolin formed through C-O and C-C coupling, respectively. **M182**–**M183** might be dimers of methyltaxiflolin and dimethylquercetin formed through C-O coupling. **M184**–**M190** might be dimers of methyltaxiflolin and dimethyl- taxifolin; **M190** was formed through C-C coupling while the other metabolites were formed through C-O coupling. **M191** was tentatively identified as a sulphate of dimers of methyltaxiflolin and dehydroxylated methyltaxifolin.

In total, 32 dimers were newly identified as metabolites of taxifolin, and this is the first report of dimers formed as metabolites of flavanonol in vivo. To the best of our knowledge, the number of dimers found is the largest in metabolism studies to date, although six honokiol dimers were previously identified from the faeces of rats [48] and seven dimer metabolites of calycosin were identified in a rat hepatic 9000× *g* supernatant incubation system [47]. Dimers found in such large numbers may have important roles in pharmacological actions of taxifolin in vivo, because dimerization to homodimer or heterodimer (the twin drug approach) is a well known strategy in medicinal chemistry [49]. Therefore, the specific structure, formation mechanism and function of these metabolites require further study.

Unequivocal structure identification of the metabolites (known as the level 1 metabolite identification) is a fundamental issue in the field of drug metabolism research. Generally speaking, to solve this issue, the metabolites have to be prepared and purified from complex biological or chemical matrix, and then be analyzed by modern spectroscopic techniques such as NMR, circular dichroism (CD) and even X-ray diffraction. Unfortunately, the process is usually very difficult, because the contents of these metabolites in the biological matrix (such as urine, feces, plasma, etc.) are very low.

Since the substrate (original compound) is known in drug metabolism research, i.e., the exact chemical structure of the substrate is definite, the LC-HRMS^n^ becomes the most common and effective method for quickly profiling and tentative identification of the metabolites to get a preliminary global view of the metabolic pathways of the original compound.

In this study, 191 metabolites of taxifolin were tentatively identified by their high resolution LC-MS^n^ data. However, it′s usually difficult or even impossible to determine regioisomers, stereoisomers and the exact metabolic site only by current MS techniques. Moreover, it is still a difficult problem to determine the exact sulphation site in flavonoids bearing a catechol moiety even by NMR technique. Fortunately, Purchartova et al. recently proposed a novel approach to solve this problem. They found that the methylation of flavonoid sulphates could be used for the direct and unequivocal determination of the position of sulphates in quercetin derivates by NMR [50]. This method is very useful for further determination of the specific structure of sulphates. According to their report, taxifolin can be metabolized to 4′-*O*-sulphate and 3′-*O*-sulphate in a ratio of 80:20 by bacterial aryl sulfotransferase from *Desulfitobacterium hafniense*. Besides, rat aryl sulfotransferase AstIV (EC 2.8.2.1) expressed recombinantly in *Escherichia coli* can biotransform taxifolin into taxifolin 3′-*O*-sulphate and quercetin 3′-*O*-sulphate [50]. These results imply that the metabolism of taxifolin is species-dependent. In addition, we also find that taxifolin can be metabolized to its sulphates (e.g., **M3**–**M11**) and quercetin sulphates (e.g., **M71**–**M75**), which is consistent with the results of rat AstIV, indicating the similarity between rat and recombinant rat AstIV.

There are four optical isomers of taxifolin because C-2 and C-3 are chiral centers, and we found two isomers metabolites (**M1**, **M2**) of taxifolin in this study. Since taxifolin has five hydroxyl groups, five sulphates could be formed at most. However, we have found nine taxifolin sulphates (**M3**–**M11**) based on LC-HRMS^n^ data, which indicates that the metabolites should include optical isomers. Because the amount of metabolites are small, we were not able to isolate sufficient metabolites and determine their exact structures. It needs more work and time to determine their exact structures by moder spectroscopic techniques in future.

### 2.3. Distribution of the Metabolites of Taxifolin in Rats

The distributions of 191 metabolites in eight rat organs (heart, liver, spleen, lung, kidney, brain, stomach and small intestine) were reported for the first time (shown in Table 3).

In total 46 metabolites were detected in eight organs, and there were 35 metabolites in the small intestine, 31 in the kidneys, 29 in the stomach, 22 in the liver, 12 in the lungs, 10 in the spleen, seven in the heart, and three in the brain. Therefore, the small intestine, kidney, stomach and liver were the main organs for the distribution of the 46 metabolites of taxifolin. The methylated metabolite **M33** was observed in all eight organs. **M11**, **M18**, **M19**, **M34** and **M49** were detected in seven organs. In total, 19 metabolites (**M2**, **M11**, **M18**–**M21**, **M23**, **M25**, **M33**–**M35**, **M43**, **M45**, **M48**–**M50**, **M52**, **M84**, **M105**) can be found in more than three organs. Therefore, these 19 metabolites were distributed more widely than the other metabolites, and they might contribute to the pharmacological activities of taxifolin in vivo.

### 2.4. Bioactivities of the Metabolites of Taxifolin

Among the metabolites of taxifolin, the nine phase I metabolites, taxifolin enantiomers (**M1** and **M2**), quercetin (**M70**), eriodictyol (**M104**), dihydrokaempferol (**M105**), 3/4-hydroxyphenylpropionic acid (**M122**, **M123**), dihydroxyphenylacetic acid (**M131**) and dihydrocaffeic acid (**M136**), and the eight phase II metabolites, quercetin-4′-*O*-sulphate (**M73**), quercetin-3′-*O*-sulphate (**M74**), quercetin-3-*O*-sulphate (**M75**), quercetin glucuronide (**M76**), isorhamnetin (**M80**), isorhamnetin-3-*O*-sulphate (**M83**), isorhamnetin disulphate (**M85**) and luteolin-7-*O*-glucuronide (**M113**), have similar bioactivities to taxifolin according to the literature (Supplemental data Table S2). The activities of 17 bioactive metabolites can cover all biological activities (about 12 in total) of taxifolin, and the number of bioactive metabolites identified appears to be the largest reported in a metabolic study of a single compound. Hence, we considered that these active metabolites were the effective forms of taxifolin and could exert their in vivo effects simultaneously with taxifolin or successively.

### 2.5. Prediction of Taxifolin Metabolite Targets

Among the 191 metabolites, the specific structures of 63 were identified tentatively by their HRMS data, reference compounds, and previous studies (detailed in Table 1). The potential targets of taxifolin and 63 metabolites were predicted using the PharmMapper server. The predicted results showed that more than 60 metabolites have the same five targets: actin, alpha skeletal muscle (target 1), cystic fibrosis transmembrane conductance regulator (target 2), UDP-glucose 4-epimerase (target 3), nucleoside diphosphate kinase (target 4), and cytosolic and pancreatic ribonuclease (target 5). This finding indicates that these metabolites may act on the same target in vivo. According to the literature, some metabolites have the same target as taxifolin; these reported targets are summarised in Table S3. For example, taxifolin, **M70** and **M80** all target phosphoinositide 3-kinase (PI3K) to suppress cancer [11,51,52].

Five of the top 300 PharmMapper-predicted target proteins of quercetin (**M70**) are reported in the literature: angiotensin-converting enzyme [53], glycogen synthase kinase-3 beta [54], beta-lactamase [55], beta-secretase 1 [56] and aspartate aminotransferase [57], as described in Table S4. Among these, glycogen synthase kinase-3 beta is a well-established target related to cancer. A total of 41 metabolites were predicted to act via this target, and six metabolites were reported to exert antitumor activity. These results indicate the reliability of this server tool and indicate that these compounds may exert the same pharmacological effects on the same targets.

We also considered the structural similarity of the 63 identified metabolites. Their chemical structures have several common fragments, summarized as follows (and detailed in Table 4): four metabolites, **M33**, **M34**, **M105** and **M109,** have fragment 1 (in red); six metabolites, **M70**, **M73**, **M74**, **M80**, **M84** and **M86,** include fragment 2 (in red); eighteen metabolites, **M33**, **M34**, **M36**, **M70**, **M73**, **M74**, **M75**, **M80**, **M83**, **M84**, **M86**, **M104**, **M105**, **M107**, **M109**, **M112**, **M114** and **M115,** contain fragment 3 (in red); and fourteen metabolites, **M35**, **M36**, **M71**, **M72**, **M75**, **M80**, **M104**, **M106**, **M113**, **M131**, **M134**, **M136**, **M142** and **M143,** include fragment 4 (in red). Metabolites with the same fragment may contain the same pharmacological groups in their structures and act at the same targets with the same effects. For example, according to the literature, among the eighteen metabolites with fragment 3, eight metabolites (**M70** [58], **M73** [59], **M74** [59], **M75** [60], **M80** [61], **M83** [59], **M104** [58] and **M105** [62]) exhibit antioxidant activity and five metabolites (**M70** [63], **M75** [64], **M80** [63], **M104** [65] and **M105** [66]) exhibit anti-inflammatory effects. Therefore, we speculated that other metabolites with the same fragment 3 may also exhibit the same bioactivities because they may act on the same individual targets.

## 3. Materials and Methods

### 3.1. Chemicals and Reagents

(2R,3R)-(+)-Taxifolin (purity > 98%) was purchased from Chengdu Must Bio-technology Co., Ltd (Chengdu, China) and used as the experiment source of taxifolin in the study. Quercetin and dihydrokaempferol were isolated in our laboratory, and the purities of these two standards were >98% as determined by high-performance liquid chromatography coupled with diode array detector analysis (area normalization method). Formic acid (Roe Scientific Inc., Newark, NJ, USA), acetonitrile (Fisher Chemicals, Fairlawn, NJ, USA), and methanol (Tianjin Damao Chemicals, Tianjin, China) were of HPLC grade. Ultrapure water was prepared using a Milli-Q water purification system (Millipore, Billerica, MA, USA). Analytical-grade sodium carboxymethyl cellulose (CMC-Na) was purchased from Tianjin Guangfu Fine Chemical Research Institute (Tianjin, China). All other reagents and chemicals were of analytical grade.

### 3.2. Animals and Drug Administration

Twelve male Sprague-Dawley rats (weighing 180–220 g) were obtained from the Experimental Animal Center of Peking University Health Science Center (Beijing, China). The rats were maintained in metabolic cages (type DXL-DL, Suzhou Fengshi Laboratory Animal Equipment Co. Ltd, Suzhou, China) and acclimatized to the facilities for 5 days prior to experiments. All rats were housed in an environmentally controlled animal room, with food and water provided *ad libitum*. The rats were randomly divided into two groups (six rats per group), a drug group and a blank group. Taxifolin was suspended in 0.5% CMC-Na solution and orally administered to the drug group at a dose of 200 mg/kg body weight, while blank group rats were orally administered 0.5% CMC-Na solution at the same volume. All rats were dosed once a day (at 9:00 a.m.) for 3 days. All animal treatments were conducted according to the Guide for the Care and Use of Laboratory Animals of the US National Institutes of Health. The animal research protocols were approved by the Biomedical Ethical Committee of Peking University (approval no. LA2015134).

### 3.3. Urine and Faeces Samples Collection and Preparation

During the administration period, urine and faeces samples from animals in the drug and blank groups were collected at 0–24 h after the first and second dosing, respectively. The urine samples were collected every 6 h from the urine collection tube (pre-filled with a small volume of methanol as preservative), a 1-fold volume of methanol was added, and samples were temporarily stored at 4 °C. Finally, all urine samples from the same group were merged into one sample and immediately evaporated to dryness at 40 °C under reduced pressure by a rotator evaporator. The dried sample was then extracted ultrasonically with a 4-fold volume of methanol for 30 min using an ultrasonic cleaner (at about 25 °C) and the extract was centrifuged at 5000 rpm for 15 min. Subsequently, the supernatant was dried in a vacuum at 40 °C. Each 1 g residue was reconstituted in 2.0 mL methanol and filtered through a 0.45-μm Millipore filter before undergoing LC-MS analysis.

Faecal samples were collected every 6 h and dried immediately using an electro-thermostatic blast oven at 40 °C. Finally, all faecal samples from the same group were merged into one sample. The dry sample was ground to powder, and 3.0 g powder from each group was mixed with 15 mL of methanol and extracted ultrasonically for 30 min three times. Next, the extracts were centrifuged at 5000 rpm for 15 min and the three supernatants were combined and evaporated to dryness under reduced pressure at 40 °C. The resulting residue was dissolved in 3.0 mL methanol and filtered through a 0.45-μm Millipore filter, and the filtrate was then subjected to LC-MS analysis.

### 3.4. Blood Sample Collection and Preparation

Blood samples were collected into heparinized tubes using a heart puncture technique under anaesthesia at 0.5, 1, and 1.5 h (two rats were sacrificed at each time point) after the last administration and were centrifuged at 5000 rpm, 4 °C for 10 min to obtain plasma. Plasma samples from the same time point within each group were combined into one sample and stored at −80 °C until processing. Upon thawing, 24 mL methanol was added to 6 mL of plasma (2 mL plasma from each of the three time points combined) in an ultrasonic bath for 30 min at about 25 °C and samples were then centrifuged at 5000 rpm for 15 min to remove precipitated protein. Next, the supernatant was concentrated to a small volume under reduced pressure at 40 °C, transferred to a clean conical tube and dried under a gentle stream of nitrogen gas at ambient temperature. The residue was then reconstituted in 300 μL of methanol and filtered through a 0.45-μm Millipore filter before undergoing LC-MS analysis.

### 3.5. Organ Sample Collection and Preparation

After collection of blood samples and rapid removal of blood from organs via heart perfusion (until the liver became yellow in colour), the heart, liver, spleen, lungs, kidneys, brain, stomach and small intestine were rapidly removed and flushed with cold normal saline (with repeated washing three times to remove surface blood and other material), dried with filter paper, and weighed. All organ samples were stored at −80 °C until further processing. The same organ samples from each group were combined into one sample and processed using a homogenizer following suspension in a four-fold (volume/mass organ wet weight) volume of cold normal saline. Next, a 6 mL aliquot of homogenate from each organ sample was added to 48 mL of methanol, extracted ultrasonically for 30 min at about 25 °C, and centrifuged at 5000 rpm for 15 min to remove the protein. The supernatant was evaporated to a small volume under reduced pressure at 40 °C and transferred into a clean tube. The supernatant was then dried under a gentle flow of nitrogen at ambient temperature, the residue was reconstituted in 1 mL methanol, and filtered through a 0.45-μm Millipore filter, and the filtrate was subjected to LC-MS analysis.

### 3.6. Instruments and Conditions for HPLC-ESI-IT-TOF-MS^n^

HPLC-ESI-IT-TOF-MS^n^ analysis was performed on a Shimadzu HPLC instrument (consisting of two LC-20AD pumps, a CTO-20A column oven, an SIL-20AC autosampler, an SPD-M20A PDA detector and a CBM-20A system controller) coupled to an IT-TOF mass spectrometer (Shimadzu, Kyoto, Japan) through an ESI interface. The chromatographic separations were carried out on an Agilent Zorbax SB-C18 column (250 mm × 4.6 mm, 5 μm) maintained at 30 °C and protected using an Agilent Security Guard column (4.0 mm × 3.0 mm, 5 μm; Agilent, Waldbronn, Germany). The mobile phase consisted of 0.1% formic acid in water (A) and acetonitrile (B). The gradient was as follows: 0 min, 2% B; 15 min, 10% B; 30 min, 15% B; 45 min, 22% B; 60 min, 35% B; 70 min, 55% B; 85 min, 82% B; 86 min, 100% B; 95 min, 100% B; 96 min, 2% B; and 105 min, 2% B (*v/v*). The volume injected was 20 μL. High-resolution mass spectra were recorded using an IT-TOF mass spectrometer programmed to carry out a full scan over *m/z* 100–1500 Da (MS) and *m/z* 50−1000 Da (MS^2^ and MS^3^) in both positive ion (PI) and negative ion (NI) detection mode. The flow velocity was maintained at 1.0 mL/min and was spilt at 0.2 mL/min through a flow divider to flow into the mass spectrometer. A trifluoroacetic acid sodium solution (2.5 mM) was used to calibrate the mass range of 50−1500 Da. The other operating parameters were set as follows: interface voltage was (+), 4.5 kV; (−), −3.5 kV; nebulizing nitrogen gas flow was 1.5 L/min; detector voltage was 1.70 kV; relative collision-induced dissociation energy was 50%; and heat block and curved desolvation line temperature was 200 °C. All data were recorded and processed using LCMS solution version 3.60, Formula Predictor version 1.2 and Accurate Mass Calculator software (Shimadzu, Kyoto, Japan).

### 3.7. Prediction of Taxifolin Metabolite Targets

The potential targets of the metabolites of taxifolin were predicted using PharmMapper server (provided by the Shanghai Institute of Materia Medica, Chinese Academy of Sciences). PharmMapper is available at http://59.78.96.61/pharmmapper.

### 3.8. Determination of the Level of Identification for All Metabolites

The definition of metabolite identification level reported in the literature was generally adopted [67]. However, considering the difference between the research field of drug metabolism and metabolomics, we tentatively modify and define the identification levels (a little different from that in [67]) as follows:

Level 1:  The metabolites are identified by comparison with reference compounds.
Level 2:  The metabolites are identified by comparison with reference literature or can be found in the Scifinder database.
Level 3:  New metabolites/compounds that could not be found in the SciFinder database.

## 4. Conclusions

A total of 191 metabolites (including 153 flavonoids and 38 phenols) of taxifolin were tentatively identified, 154 of whom were new metabolites of taxifolin. Furthermore, 69 metabolites were new compounds that were not found in the SciFinder database, including 12 taxifolin conjugates, 22 methyl taxifolin derivatives, four phenolic acid derivatives and 31 dimers. To our knowledge, this is the first report of a single compound biotransformed into more than 100 metabolites in vivo.

The major metabolic reactions of taxifolin in rats included ring-cleavage, sulphation, glucuronidation, methylation and dimerization. Furthermore, acetylamination and pyroglutamic acid conjugation were new metabolic reactions not described in any previous metabolism studies.

A total of 17 metabolites had similar bioactivites to taxifolin. The PharmMapper prediction showed that more than 60 metabolites had the same five targets. This suggested that the effective forms [68] of taxifolin are not only the parent form, but also the metabolites arising from it in vivo. And moreover, the effective metabolites are much larger in number than that of the imagination. These metabolites may exert the same pharmacological effects as taxifolin on the same targets. We therefore speculated that they might play the same role as the parent form through an additive effect [69]. These findings enhance the understanding of taxifolin metabolism and may provide further evidence of the beneficial effects of taxifolin and its derivatives in foods and other supplements. The study outcomes indicate that the metabolites and biotransformation of those bioactive constituents in foods and herbs require increased attention, especially to evaluate the biological activity of their metabolites. Our results may also provide a scientific support for our hypothesis of the traditional Chinese medicines (TCMs) efficacy theory [68], whereby TCMs exert their effects through the additive effects of numerous effective forms (including numerous original constituents and metabolites) on the same target, with synergistic effects based on the overall action of the additive effects on individual targets. Namely, numerous effective forms of incalculable constituents and their metabolites might participate in the process of pharmacodynamic action and could work together like an “army group”. Our results may also provide an explanation to the question of how TCMs can exert pharmacological actions when the blood concentrations of their pharmacodynamic substances (constituents or their metabolites) are usually very low.

## Figures and Tables

**Table 1:** Retention time (t_R_), HRMS data, molecular formula, and identification of taxifolin and its 191 metabolites in rats urine, plasma, faeces by HPLC-ESI-IT-TOF-MS^n^.

No.	t_R_ (min)	Formula	Ion	Meas. *m/z*	Pred. *m/z*	Diff (ppm)	DBE	Urine	Plasma	Faeces	Identification Level	Identification
**TAX**	41.023	C_15_H_12_O_7_	[M − H]^−^	303.0521	303.0510	3.63	10	▲	▲	▲	Level 1	Taxifolin (parent compound)
					**Metabolites having the aglycone of taxifolin or its isomers (M1–M32); two bioactive metabolites**							
**M1 ^a,b^**	40.508	C_15_H_12_O_7_	[M − H]^−^	303.0502	303.0510	−2.64	10	-	▲	▲	Level 2	Taxifolin isomer 1
**M2 ^a,b^**	42.883	C_15_H_12_O_7_	[M − H]^−^	303.0517	303.0510	2.31	10	▲	▲	▲	Level 2	Taxifolin isomer 2
**M3 ^b^**	21.517	C_15_H_12_O_10_S	[M − H]^−^	383.0080	383.0078	0.52	10	▲	-	-	Level 2	Taxifolin sulphate 1
**M4 ^b^**	31.242	C_15_H_12_O_10_S	[M − H]^−^	383.0089	383.0078	2.87	10	▲	-	-	Level 2	Taxifolin sulphate 2
**M5 ^b^**	32.145	C_15_H_12_O_10_S	[M − H]^−^	383.0073	383.0078	−1.31	10	-	▲	▲	Level 2	Taxifolin sulphate 3
**M6 ^b^**	35.292	C_15_H_12_O_10_S	[M − H]^−^	383.0078	383.0078	0.00	10	▲	-	-	Level 2	Taxifolin sulphate 4
**M7 ^b^**	36.717	C_15_H_12_O_10_S	[M − H]^−^	383.0079	383.0078	0.26	10	▲	▲	▲	Level 2	Taxifolin sulphate 5
**M8 ^b^**	37.925	C_15_H_12_O_10_S	[M − H]^−^	383.0070	383.0078	−2.09	10	▲	▲	-	Level 2	Taxifolin sulphate 6
**M9 ^b^**	39.375	C_15_H_12_O_10_S	[M − H]^−^	383.0087	383.0078	2.35	10	▲	-	▲	Level 2	Taxifolin sulphate 7
**M10 ^b^**	41.192	C_15_H_12_O_10_S	[M − H]^−^	383.0086	383.0078	2.09	10	▲	▲	▲	Level 2	Taxifolin sulphate 8
**M11 ^b^**	43.000	C_15_H_12_O_10_S	[M − H]^−^	383.0082	383.0078	1.04	10	▲	▲	▲	Level 2	Taxifolin sulphate 9
**M12 ^c^**	24.592	C_15_H_12_O_13_S_2_	[M − H]^−^	462.9644	462.9647	−0.65	10	▲	-	-	Level 3	Taxifolin disulphate 1
**M13 ^c^**	27.458	C_15_H_12_O_13_S_2_	[M − H]^−^	462.9670	462.9647	4.97	10	▲	-	-	Level 3	Taxifolin disulphate 2
**M14 ^c^**	31.075	C_15_H_12_O_13_S_2_	[M − H]^−^	462.9639	462.9647	−1.73	10	▲	-	-	Level 3	Taxifolin disulphate 3
**M15 ^c^**	39.767	C_15_H_12_O_13_S_2_	[M − H]^−^	462.9656	462.9647	1.94	10	▲	-	-	Level 3	Taxifolin disulphate 4
**M16 ^c^**	16.252	C_20_H_19_NO_13_S	[M − H]^−^	512.0509	512.0504	0.98	12	▲	-	-	Level 3	Taxifolin sulphate and pyroglutamic acid conjugate
**M17 ^b^**	15.408	C_21_H_20_O_13_	[M − H]^−^	479.0834	479.0831	0.63	12	▲	-	-	Level 2	Taxifolin glucuronide 1
**M18 ^b^**	18.637	C_21_H_20_O_13_	[M − H]^−^	479.0850	479.0831	3.97	12	-	▲	-	Level 2	Taxifolin glucuronide 2
**M19 ^b^**	20.253	C_21_H_20_O_13_	[M − H]^−^	479.0847	479.0831	3.34	12	▲	▲	-	Level 2	Taxifolin glucuronide 3
**M20 ^b^**	21.370	C_21_H_20_O_13_	[M − H]^−^	479.0843	479.0831	2.50	12	▲	▲	-	Level 2	Taxifolin glucuronide 4
**M21 ^b^**	22.267	C_21_H_20_O_13_	[M − H]^−^	479.0838	479.0831	1.46	12	▲	▲	-	Level 2	Taxifolin glucuronide 5
**M22 ^b^**	22.587	C_21_H_20_O_13_	[M − H]^−^	479.0847	479.0831	3.34	12	-	▲	-	Level 2	Taxifolin glucuronide 6
**M23 ^b^**	31.862	C_21_H_20_O_13_	[M − H]^−^	479.0830	479.0831	−0.21	12	▲	▲	-	Level 2	Taxifolin glucuronide 7
**M24 ^b^**	34.742	C_21_H_20_O_13_	[M − H]^−^	479.0832	479.0831	0.21	12	▲	-	-	Level 2	Taxifolin glucuronide 8
**M25 ^b^**	37.267	C_21_H_20_O_13_	[M − H]^−^	479.0834	479.0831	0.63	12	▲	▲	-	Level 2	Taxifolin glucuronide 9
**M26 ^c^**	13.888	C_21_H_20_O_16_S	[M − H]^−^	559.0388	559.0399	−1.97	12	-	▲	-	Level 3	Taxifolin glucuronide sulphate 1
**M27 ^c^**	16.703	C_21_H_20_O_16_S	[M − H]^−^	559.0423	559.0399	4.29	12	▲	▲	-	Level 3	Taxifolin glucuronide sulphate 2
**M28 ^c^**	19.928	C_21_H_20_O_16_S	[M − H]^−^	559.0406	559.0399	1.25	12	▲	▲	-	Level 3	Taxifolin glucuronide sulphate 3
**M29 ^c^**	21.812	C_21_H_20_O_16_S	[M − H]^−^	559.0411	559.0399	2.15	12	-	▲	-	Level 3	Taxifolin glucuronide sulphate 4
**M30 ^c^**	23.087	C_21_H_20_O_16_S	[M − H]^−^	559.0418	559.0399	3.40	12	▲	▲	-	Level 3	Taxifolin glucuronide sulphate 5
**M31 ^c^**	24.762	C_21_H_20_O_16_S	[M − H]^−^	559.0425	559.0399	4.65	12	-	▲	-	Level 3	Taxifolin glucuronide sulphate 6
**M32 ^c^**	25.797	C_21_H_20_O_16_S	[M − H]^−^	559.0411	559.0399	2.86	12	-	-	-	Level 3	Taxifolin glucuronide sulphate 7
					**Metabolites having the aglycone of methyl taxifolin (M33–M69)**							
**M33 ^b,d^**	50.292	C_16_H_14_O_7_	[M − H]^−^	317.0675	317.0667	2.52	10	▲	▲	▲	Level 2	3′-*O*-Methyltaxifolin
**M34 ^b,d^**	51.350	C_16_H_14_O_7_	[M − H]^−^	317.0673	317.0667	1.89	10	▲	▲	▲	Level 2	4′-*O*-Methyltaxifolin
**M35 ^b,d^**	52.875	C_16_H_14_O_7_	[M − H]^−^	317.0667	317.0667	0.00	10	▲	▲	▲	Level 2	7-*O*-Methyltaxifolin
**M36 ^b,d^**	53.592	C_16_H_14_O_7_	[M − H]^−^	317.0660	317.0667	−2.21	10	▲	-	-	Level 2	3-*O*-Methyltaxifolin
**M37 ^c^**	28.575	C_16_H_14_O_10_S	[M − H]^−^	397.0243	397.0235	2.01	10	▲	-	-	Level 3	Methyl taxifolin sulphate 1
**M38 ^c^**	33.942	C_16_H_14_O_10_S	[M − H]^−^	397.0240	397.0235	1.26	10	▲	▲	-	Level 3	Methyl taxifolin sulphate 2
**M39 ^c^**	34.420	C_16_H_14_O_10_S	[M − H]^−^	397.0247	397.0235	3.02	10	-	▲	-	Level 3	Methyl taxifolin sulphate 3
**M40 ^c^**	35.858	C_16_H_14_O_10_S	[M − H]^−^	397.0253	397.0235	4.53	10	▲	-	-	Level 3	Methyl taxifolin sulphate 4
**M41 ^c^**	38.092	C_16_H_14_O_10_S	[M − H]^−^	397.0241	397.0235	1.51	10	▲	-	-	Level 3	Methyl taxifolin sulphate 5
**M42 ^c^**	40.283	C_16_H_14_O_10_S	[M − H]^−^	397.0233	397.0235	−0.50	10	▲	▲	▲	Level 3	Methyl taxifolin sulphate 6
**M43 ^c^**	41.817	C_16_H_14_O_10_S	[M − H]^−^	397.0241	397.0235	1.51	10	▲	▲	▲	Level 3	Methyl taxifolin sulphate 7
**M44 ^c^**	42.717	C_16_H_14_O_10_S	[M − H]^−^	397.0230	397.0235	−1.26	10	▲	-	-	Level 3	Methyl taxifolin sulphate 8
**M45 ^c^**	43.600	C_16_H_14_O_10_S	[M − H]^−^	397.0235	397.0235	0.00	10	▲	▲	-	Level 3	Methyl taxifolin sulphate 9
**M46 ^c^**	45.558	C_16_H_14_O_10_S	[M − H]^−^	397.0238	397.0235	0.76	10	▲	▲	▲	Level 3	Methyl taxifolin sulphate 10
**M47 ^b^**	23.520	C_22_H_22_O_13_	[M − H]^−^	493.0973	493.0988	−3.04	12	-	▲	-	Level 2	Methyl taxifolin glucuronide 1
**M48 ^b^**	25.212	C_22_H_22_O_13_	[M − H]^−^	493.1012	493.0988	4.87	12	-	▲	-	Level 2	Methyl taxifolin glucuronide 2
**M49 ^b^**	26.687	C_22_H_22_O_13_	[M − H]^−^	493.1012	493.0988	4.87	12	-	▲	-	Level 2	Methyl taxifolin glucuronide 3
**M50 ^b^**	30.383	C_22_H_22_O_13_	[M − H]^−^	493.1012	493.0988	4.87	12	▲	▲	-	Level 2	Methyl taxifolin glucuronide 4
**M51 ^b^**	33.395	C_22_H_22_O_13_	[M − H]^−^	493.1007	493.0988	3.85	12	-	▲	-	Level 2	Methyl taxifolin glucuronide 5
**M52 ^b^**	35.692	C_22_H_22_O_13_	[M − H]^−^	493.0998	493.0988	2.03	12	▲	▲	-	Level 2	Methyl taxifolin glucuronide 6
**M53 ^b^**	36.025	C_22_H_22_O_13_	[M − H]^−^	493.1004	493.0988	3.24	12	▲	-	-	Level 2	Methyl taxifolin glucuronide 7
**M54 ^b^**	37.600	C_22_H_22_O_13_	[M − H]^−^	493.0998	493.0988	2.03	12	▲	-	-	Level 2	Methyl taxifolin glucuronide 8
**M55 ^b^**	42.375	C_22_H_22_O_13_	[M − H]^−^	493.1008	493.0988	4.06	12	▲	-	-	Level 2	Methyl taxifolin glucuronide 9
**M56**	34.742	C_22_H_22_O_16_S	[M − H]^−^	573.0560	573.0556	0.70	12	▲	-	-	Level 2	Methyl taxifolin glucuronide sulphate 1
**M57**	37.158	C_22_H_22_O_16_S	[M − H]^−^	573.0533	573.0556	−4.01	12	▲	-	-	Level 2	Methyl taxifolin glucuronide sulphate 2
**M58 ^c^**	16.490	C_21_H_21_NO_10_	[M − H]^−^	446.1107	446.1093	3.14	12	▲	-	-	Level 3	Methyl taxifolin pyroglutamic acid conjugate 1
**M59 ^c^**	18.483	C_21_H_21_NO_10_	[M − H]^−^	446.1086	446.1093	−1.57	12	▲	-	-	Level 3	Methyl taxifolin pyroglutamic acid conjugate 2
**M60 ^c^**	37.848	C_16_H_14_O_11_S	[M − H]^−^	413.0200	413.0184	3.87	10	-	▲	-	Level 3	Hydroxylated methyl taxifolin sulphate 1
**M61 ^c^**	41.943	C_16_H_14_O_11_S	[M − H]^−^	413.0175	413.0184	−2.18	10	-	▲	-	Level 3	Hydroxylated methyl taxifolin sulphate 2
**M62 ^c^**	42.375	C_16_H_14_O_11_S	[M − H]^−^	413.0198	413.0184	3.39	10	▲	-	-	Level 3	Hydroxylated methyl taxifolin sulphate 3
**M63 ^c^**	42.660	C_16_H_14_O_11_S	[M − H]^−^	413.0191	413.0184	1.69	10	-	▲	-	Level 3	Hydroxylated methyl taxifolin sulphate 4
**M64 ^c^**	55.808	C_16_H_14_O_9_	[M − H]^−^	349.0580	349.0565	4.30	10	▲	-	▲	Level 3	Methylated and dihydroxylated taxifolin 1
**M65 ^c^**	56.608	C_16_H_14_O_9_	[M − H]^−^	349.0551	349.0565	−4.01	10	-	-	-	Level 3	Methylated and dihydroxylated taxifolin 2
**M66 ^c^**	17.170	C_22_H_22_O_15_	[M − H]^−^	525.0865	525.0886	−4.00	12	-	▲	-	Level 3	Methylated and dihydroxylated taxifolin glucuronide 1
**M67 ^c^**	17.887	C_22_H_22_O_15_	[M − H]^−^	525.0908	525.0886	4.19	12	-	▲	-	Level 3	Methylated and dihydroxylated taxifolin glucuronide 2
**M68 ^c^**	18.637	C_22_H_22_O_15_	[M − H]^−^	525.0890	525.0886	0.76	12	-	▲	-	Level 3	Methylated and dihydroxylated taxifolin glucuronide 3
**M69 ^c^**	19.178	C_22_H_22_O_15_	[M − H]^−^	525.0911	525.0886	4.76	12	-	▲	-	Level 3	Methylated and dihydroxylated taxifolin glucuronide 4
					**Metabolites having the aglycone of quercetin(M70–M103); eight bioactive metabolites**							
**M70 ^a,d^**	**58.150**	C_15_H_10_O_7_	[M − H]^−^	301.0350	301.0354	−1.33	11	▲	-	▲	Level 2	Quercetin
**M71 ^d^**	**51.583**	C_15_H_10_O_10_S	[M − H]^−^	380.9933	380.9922	0.26	11	▲	-	-	Level 2	Quercetin-5 *-O-*sulphate
**M72 ^d^**	**52.647**	C_15_H_10_O_10_S	[M − H]^−^	380.9932	380.9922	2.89	11	-	-	-	Level 2	Quercetin-7-*O*-sulphate
**M73 ^a,d^**	**56.300**	C_15_H_10_O_10_S	[M − H]^−^	380.9922	380.9922	0.00	11	▲	-	▲	Level 2	Quercetin-4′-*O*-sulphate
**M74 ^a,d^**	**57.033**	C_15_H_10_O_10_S	[M − H]^−^	380.9932	380.9922	2.62	11	▲	-	▲	Level 2	Quercetin-3′-*O*-sulphate
**M75 ^a,d^**	**58.173**	C_15_H_10_O_10_S	[M − H]^−^	380.9937	380.9922	3.94	11	-	-	-	Level 2	Quercetin-3-*O*-sulphate
**M76 ^a^**	**37.542**	C_21_H_18_O_13_	[M − H]^−^	477.0688	477.0675	2.72	13	▲	-	-	Level 2	Quercetin glucuronide
**M77**	**40.727**	C_21_H_18_O_16_S	[M − H]^−^	557.0252	557.0243	1.62	13	-	▲	-	Level 2	Quercetin glucuronide sulphate 1
**M78**	**41.068**	C_21_H_18_O_16_S	[M − H]^−^	557.0268	557.0243	4.49	13	-	▲	-	Level 2	Quercetin glucuronide sulphate 2
**M79**	**41.443**	C_21_H_18_O_16_S	[M − H]^−^	557.0269	557.0243	4.67	13	-	▲	-	Level 2	Quercetin glucuronide sulphate 3
**M80 ^a,d^**	**65.417**	C_16_H_12_O_7_	[M − H]^−^	315.0503	315.0510	−2.22	11	▲	-	▲	Level 2	Isorhamnetin
**M81 ^d^**	**48.633**	C_16_H_12_O_10_S	[M − H]^−^	395.0081	395.0078	0.76	11	▲	▲	-	Level 2	Isorhamnetin-5-*O*-sulphate
**M82 ^d^**	**56.917**	C_16_H_12_O_10_S	[M − H]^−^	395.0082	395.0078	1.01	11	▲	-	▲	Level 2	Isorhamnetin-7-*O*-sulphate
**M83 ^a,d^**	**58.042**	C_16_H_12_O_10_S	[M − H]^−^	395.0085	395.0078	1.77	11	▲	▲	▲	Level 2	Isorhamnetin-3-*O*-sulphate
**M84 ^d^**	**58.922**	C_16_H_12_O_10_S	[M − H]^−^	395.0082	395.0078	1.01	11	-	▲	-	Level 2	Isorhamnetin-4′-*O*-sulphate
**M85 ^a^**	**48.308**	C_16_H_12_O_13_S_2_	[M − H]^−^	474.9658	474.9647	2.32	11	▲	▲	-	Level 2	Isorhamnetin disulphate
**M86 ^d^**	**49.212**	C_22_H_20_O_13_	[M − H]^−^	491.0852	491.0831	4.28	13	-	▲	-	Level 2	Isorhamnetin-4′-*O*-glucuronide
**M87 ^d^**	**50.428**	C_22_H_20_O_13_	[M − H]^−^	491.0836	491.0831	1.02	13	-	▲	-	Level 2	Isorhamnetin-7-*O*-glucuronide
**M88**	**40.143**	C_22_H_20_O_16_S	[M − H]^−^	571.0381	571.0399	−3.15	13	-	▲	-	Level 2	Isorhamnetin glucuronide sulphate 1
**M89**	41.118	C_22_H_20_O_16_S	[M − H]^−^	571.0413	571.0399	2.45	13	-	▲	-	Level 2	Isorhamnetin glucuronide sulphate 2
**M90**	44.673	C_22_H_20_O_16_S	[M − H]^−^	571.0395	571.0399	−0.70	13	-	▲	-	Level 2	Isorhamnetin glucuronide sulphate 3
**M91**	45.392	C_22_H_20_O_16_S	[M − H]^−^	571.0419	571.0399	3.50	13	-	-	-	Level 2	Isorhamnetin glucuronide sulphate 4
**M92**	27.987	C_15_H_10_O_11_S	[M − H]^−^	396.9882	396.9871	2.77	11	-	▲	-	Level 2	Hydroxylated quercetin sulphate 1
**M93**	28.487	C_15_H_10_O_11_S	[M − H]^−^	396.9868	396.9871	−0.76	11	-	▲	-	Level 2	Hydroxylated quercetin sulphate 2
**M94**	29.028	C_15_H_10_O_11_S	[M − H]^−^	396.9876	396.9871	1.26	11	-	▲	-	Level 2	Hydroxylated quercetin sulphate 3
**M95**	15.930	C_21_H_18_O_14_	[M − H]^−^	493.0642	493.0624	3.65	13	-	▲	-	Level 2	Hydroxylated quercetin glucuronide 1
**M96**	17.720	C_21_H_18_O_14_	[M − H]^−^	493.0601	493.0624	−4.66	13	-	▲	-	Level 2	Hydroxylated quercetin glucuronide 2
**M97**	39.160	C_16_H_12_O_11_S	[M − H]^−^	411.0022	411.0028	−1.46	11	-	▲	-	Level 2	Hydroxylated isorhamnetin sulphate 1
**M98**	39.710	C_16_H_12_O_11_S	[M − H]^−^	411.0043	411.0028	3.65	11	-	▲	-	Level 2	Hydroxylated isorhamnetin sulphate 2
**M99**	40.193	C_16_H_12_O_11_S	[M − H]^−^	411.0039	411.0028	2.68	11	-	▲	-	Level 2	Hydroxylated isorhamnetin sulphate 3
**M100**	59.017	C_16_H_12_O_11_S	[M − H]^−^	411.0030	411.0028	0.49	11	▲	▲	-	Level 2	Hydroxylated isorhamnetin sulphate 4
**M101**	25.103	C_22_H_20_O_14_	[M − H]^−^	507.0790	507.0780	1.97	13	-	▲	-	Level 2	Hydroxylated isorhamnetin glucuronide 1
**M102**	25.728	C_22_H_20_O_14_	[M − H]^−^	507.0758	507.0780	−4.34	13	-	▲	-	Level 2	Hydroxylated isorhamnetin glucuronide 2
**M103**	26.570	C_22_H_20_O_14_	[M − H]^−^	507.0805	507.0780	4.93	13	-	▲	-	Level 2	Hydroxylated isorhamnetin glucuronide 3
					**Metabolites having the aglycone of dehydroxylated taxifolin (M104–M112); two bioactive metabolites**							
**M104 ^a,b,d^**	40.733	C_15_H_12_O_6_	[M − H]^−^	287.0557	287.0561	−1.39	10	-	-	▲	Level 2	Eriodictyol
**M105 ^a,b,d^**	49.442	C_15_H_12_O_6_	[M − H]^−^	287.0555	287.0561	−2.09	10	▲	-	▲	Level 2	Dihydrokaempferol
**M106 ^d^**	37.325	C_15_H_12_O_9_S	[M − H]^−^	367.0128	367.0129	−0.27	10	▲	-	-	Level 2	Eriodictyol-7-*O*-sulphate
**M107 ^d^**	37.708	C_15_H_12_O_9_S	[M − H]^−^	367.0144	367.0129	4.09	10	▲	-	▲	Level 2	Dihydrokaempferol-7-*O*-sulphate
**M108 ^d^**	38.200	C_15_H_12_O_9_S	[M − H]^−^	367.0144	367.0129	4.09	10	▲	-	▲	Level 2	Eriodictyol-3′-*O*-sulphate
**M109 ^d^**	40.383	C_15_H_12_O_9_S	[M − H]^−^	367.0123	367.0129	−1.63	10	-	-	-	Level 2	Dihydrokaempferol-4′-*O*-sulphate
**M110**	28.045	C_21_H_20_O_12_	[M − H]^−^	463.0907	463.0882	5.40	12	-	▲	-	Level 2	Dehydroxylated taxifolin glucuronide 1
**M111**	28.753	C_21_H_20_O_12_	[M − H]^−^	463.0856	463.0882	−5.61	12	-	▲	-	Level 2	Dehydroxylated taxifolin glucuronide 2
**M112 ^d^**	28.970	C_21_H_20_O_12_	[M − H]^−^	463.0888	463.0882	1.30	12	-	▲	-	Level 2	Dihydrokaempferol-4′-*O*-glucuronide
					**Metabolites formed through dehydration and glucuronidation (M113–M116); one bioactive metabolite**							
**M113 ^a,d^**	16.017	C_21_H_18_O_12_	[M + NH_2_]^−^	478.1007	478.0991	3.35	13	▲	-	-	Level 2	Luteolin-7-*O*-glucuronide
**M114 ^d^**	16.525	C_21_H_18_O_12_	[M + NH_2_]^−^	478.1007	478.0991	3.35	13	▲	-	-	Level 2	Luteolin-3′/4′-*O*-glucuronide
**M115 ^d^**	17.425	C_21_H_18_O_12_	[M + NH_2_]^−^	478.1014	478.0991	4.81	13	▲	-	-	Level 2	Luteolin-3′/4′-*O*-glucuronide
**M116**	23.625	C_22_H_20_O_12_	[M + NH_2_]^−^	492.1160	492.1147	2.64	13	▲	-	-	Level 2	Methyl luteolin glucuronide
					**Metabolites having the aglycone of hydrogenated taxifolin (M117–M121)**							
**M117**	43.883	C_15_H_14_O_7_	[M − H]^−^	305.0652	305.0667	−4.92	9	▲	-	▲	Level 2	Hydrogenated taxifolin
**M118**	52.325	C_16_H_16_O_7_	[M − H]^−^	319.0813	319.0823	−3.13	9	▲	-	▲	Level 2	Hydrogenated methyl taxifolin
**M119**	38.567	C_15_H_14_O_10_S	[M − H]^−^	385.0224	385.0235	−2.86	9	-	-	▲	Level 2	Hydrogenated taxifolin sulphate 1
**M120**	43.433	C_15_H_14_O_10_S	[M − H]^−^	385.0224	385.0235	−2.86	9	-	-	▲	Level 2	Hydrogenated taxifolin sulphate 2
**M121**	45.442	C_15_H_14_O_10_S	[M − H]^−^	385.0227	385.0235	−2.08	9	▲	-	▲	Level 2	Hydrogenated taxifolin sulphate 3
					**Phenolic acid metabolites through ring cleavage (M122–M159); four bioactive metabolites**							
**M122 ^a,b,d^**	35.317	C_9_H_10_O_3_	[M − H]^−^	165.0555	165.0557	−1.21	5	-	-	▲	Level 2	3/4-Hydroxyphenylpropionic acid
**M123 ^a,d^**	35.917	C_9_H_10_O_3_	[M − H]^−^	165.0559	165.0557	1.21	5	▲	-	▲	Level 2	3/4-Hydroxyphenylpropionic acid
**M124 ^d^**	21.712	C_9_H_10_O_6_S	[M − H]^−^	245.0132	245.0125	2.86	5	▲	-	-	Level 2	4-Hydroxyphenylpropionic acid sulphate
**M125 ^d^**	23.683	C_9_H_10_O_6_S	[M − H]^−^	245.0133	245.0125	3.27	5	▲	▲	-	Level 2	3-Hydroxyphenylpropionic acid sulphate
**M126 ^d^**	23.787	C_15_H_18_O_9_	[M − H]^−^	341.0866	341.0878	−1.76	7	▲	-	-	Level 2	3/4-Hydroxyphenylpropionic acid glucuronide
**M127 ^d^**	24.078	C_15_H_18_O_9_	[M − H]^−^	341.0891	341.0878	3.81	7	▲	-	-	Level 2	3/4-Hydroxyphenylpropionic acid glucuronide
**M128 ^d^**	22.325	C_9_H_8_O_6_S	[M − H]^−^	242.9969	242.9969	0.00	6	▲	-	-	Level 2	*p/m*-Coumaric acid sulphate
**M129 ^d^**	25.758	C_9_H_8_O_6_S	[M − H]^−^	242.9972	242.9969	1.23	6	▲	▲	-	Level 2	*p/m*-Coumaric acid sulphate
**M130 ^d^**	27.067	C_9_H_8_O_6_S	[M − H]^−^	242.9971	242.9969	0.82	6	▲	-	-	Level 2	*p/m*-Coumaric acid sulphate
**M131 ^a,b,d^**	16.490	C8H8O4	[M − H]^−^	167.0349	167.0350	−0.60	5	-	-	▲	Level 2	Dihydroxyphenylacetic acid
**M132 ^d^**	16.258	C_8_H_8_O_7_S	[M − H]^−^	246.9927	246.9918	3.64	5	▲	-	▲	Level 2	Dihydroxyphenylacetic acid sulfae 1
**M133 ^d^**	15.800	C_8_H_8_O_7_S	[M − H]^−^	246.9927	246.9918	3.64	5	-	-	▲	Level 2	Dihydroxyphenylacetic acid sulfae 2
**M134 ^d^**	16.933	C_8_H_8_O_7_S	[M − H]^−^	246.9920	246.9918	0.81	5	▲	-	▲	Level 2	Dihydroxyphenylacetic acid sulfae 3
**M135 ^d^**	18.108	C_9_H_10_O_7_S	[M − H]^−^	261.0073	261.0074	−0.38	5	▲	-	-	Level 2	Homovanillic acid sulphate
**M136 ^d^**	22.508	C_9_H_10_O_4_	[M − H]^−^	181.0504	181.0506	−1.10	5	-	-	▲	Level 2	Dihydrocaffeic acid
**M137 ^d^**	20.033	C_9_H_10_O_7_S	[M − H]^−^	261.0082	261.0074	3.07	5	▲	-	-	Level 2	Dihydrocaffeic acid sulphate 1
**M138 ^d^**	20.942	C_9_H_10_O_7_S	[M − H]^−^	261.0084	261.0074	3.83	5	▲	-	-	Level 2	Dihydrocaffeic acid sulphate 2
**M139 ^d^**	13.108	C_11_H_13_NO_5_	[M − H]^−^	238.0720	238.0721	−0.42	6	-	-	▲	Level 2	Caffeic acid acetamide 1
**M140 ^d^**	13.592	C_11_H_13_NO_5_	[M − H]^−^	238.0724	238.0721	1.26	6	-	-	▲	Level 2	Caffeic acid acetamide 2
**M141 ^d^**	13.858	C_11_H_13_NO_5_	[M − H]^−^	238.0728	238.0721	2.94	6	-	-	▲	Level 2	Caffeic acid acetamide 3
**M142 ^d^**	11.692	C_9_H_10_O_5_	[M − H]^−^	197.0461	197.0455	3.04	5	▲	-	▲	Level 2	3-(3,4-Dihydroxyphenyl)-3-hydroxypropanoic acid
**M143 ^d^**	12.658	C_9_H_10_O_5_	[M − H]^−^	197.0456	197.0455	0.51	5	▲	-	▲	Level 2	3-(3,4-Dihydroxyphenyl)-2-hydroxypropanoic acid
**M144 ^d^**	12.700	C_9_H_10_O_8_S	[M − H]^−^	277.0024	277.0024	0.00	5	▲	-	▲	Level 2	Caffeic acid hydrate sulphate 1
**M145 ^d^**	13.433	C_9_H_10_O_8_S	[M − H]^−^	277.0025	277.0024	0.36	5	▲	-	▲	Level 2	Caffeic acid hydrate sulphate 2
**M146 ^d^**	22.667	C_10_H_12_O_7_S	[M − H]^−^	275.0236	275.0231	1.82	5	▲	-	-	Level 2	Dihydrogen ferulic acid sulphate
**M147 ^d^**	15.810	C_10_H_12_O_8_S	[M − H]^−^	291.0174	291.0180	3.78	5	▲	-	-	Level 2	Ferulic acid hydrate sulphate 1
**M148 ^d^**	16.233	C_10_H_12_O_8_S	[M − H]^−^	291.0184	291.0180	1.37	5	▲	-	-	Level 2	Ferulic acid hydrate sulphate 2
**M149**	25.208	C_7_H_8_O_5_S	[M − H]^−^	203.0021	203.0020	0.49	4	▲	-	-	Level 2	Hydroxybenzyl alcohol sulphate
**M150 ^d^**	29.025	C_13_H_16_O_8_	[M − H]^−^	299.0773	299.0772	0.33	6	▲	▲	-	Level 2	Hydroxybenzyl alcohol glucuronide 1
**M151 ^d^**	29.717	C_13_H_16_O_8_	[M − H]^−^	299.0771	299.0772	−0.33	6	▲	▲	-	Level 2	Hydroxybenzyl alcohol glucuronide 2
**M152 ^c,d^**	18.795	C_13_H_16_O_11_S	[M − H]^−^	379.0336	379.0341	−1.32	6	-	▲	-	Level 3	Hydroxybenzyl alcohol glucuronide sulphate 1
**M153 ^c,d^**	21.095	C_13_H_16_O_11_S	[M − H]^−^	379.0337	379.0341	−1.06	6	-	▲	-	Level 3	Hydroxybenzyl alcohol glucuronide sulphate 2
**M154 ^c,d^**	33.083	C_8_H_10_O_5_S	[M − H]^−^	217.0168	217.0176	−3.69	4	▲	-	-	Level 3	Methyl hydroxybenzyl alcohol sulphate 1
**M155 ^c,d^**	34.625	C_8_H_10_O_5_S	[M − H]^−^	217.0181	217.0176	2.30	4	▲	-	-	Level 3	Methyl hydroxybenzyl alcohol sulphate 2
**M156 ^d^**	17.512	C_7_H_6_O_6_S	[M − H]^−^	216.9822	216.9812	4.61	5	-	▲	-	Level 2	3/4-Hydroxy benzoic acid sulphate
**M157 ^d^**	17.937	C_7_H_6_O_6_S	[M − H]^−^	216.9810	216.9812	−0.92	5	-	▲	-	Level 2	3/4-Hydroxy benzoic acid sulphate
**M158 ^d^**	30.987	C_8_H_8_O_7_S	[M − H]^−^	246.9914	246.9918	−1.62	5	-	▲	-	Level 2	Vanillic acid sulphate
**M159 ^d^**	31.978	C_8_H_8_O_7_S	[M − H]^−^	246.9909	246.9918	−3.64	5	-	▲	-	Level 2	Isovanillic acid sulphate
					**Metabolites formed through polymerization(M160–M191)**							
**M160**	61.342	C_31_H_24_O_13_	[M − H]^−^	603.1151	603.1144	1.16	20	▲	-	-	Level 2	Dimer of taxiflolin and dehydroxylated methyl taxifolin
**M161 ^c^**	55.533	C_31_H_24_O_14_	[M − H]^−^	619.1063	619.1093	−4.85	20	▲	-	-	Level 3	Dimer of taxiflolin and methyl taxifolin 1
**M162 ^c^**	60.600	C_31_H_24_O_14_	[M − H]^−^	619.1090	619.1093	−0.48	20	▲	-	-	Level 3	Dimer of taxiflolin and methyl taxifolin 2
**M163 ^c^**	64.608	C_32_H_26_O_14_	[M − H]^−^	633.1249	633.1250	−0.16	20	▲	-	-	Level 3	Dimer of taxiflolin and dimethyl taxifolin
**M164 ^c^**	56.025	C_31_H_24_O_17_S	[M − H]^−^	699.0699	699.0661	5.44	20	▲	-	-	Level 3	Dimer of taxiflolin and methyl taxifolin sulphate 1
**M165 ^c^**	56.750	C_31_H_24_O_17_S	[M − H]^−^	699.0671	699.0661	1.43	20	▲	-	-	Level 3	Dimer of taxiflolin and methyl taxifolin sulphate 2
**M166 ^c^**	60.817	C_31_H_24_O_17_S	[M − H]^−^	699.0678	699.0661	2.43	20	▲	▲	-	Level 3	Dimer of taxiflolin and methyl taxifolin sulphate 3
**M167 ^c^**	59.725	C_32_H_26_O_17_S	[M − H]^−^	713.0844	713.0818	3.65	20	▲	-	-	Level 3	Dimer of taxiflolin and dimethyl taxifolin sulphate 1
**M168 ^c^**	60.167	C_32_H_26_O_17_S	[M − H]^−^	713.0839	713.0818	2.94	20	▲	-	-	Level 3	Dimer of taxiflolin and dimethyl taxifolin sulphate 2
**M169 ^c^**	64.125	C_32_H_26_O_17_S	[M − H]^−^	713.0843	713.0818	3.51	20	▲	▲	-	Level 3	Dimer of taxiflolin and dimethyl taxifolin sulphate 3
**M170 ^c^**	60.650	C_32_H_26_O_13_	[M − H]^−^	617.1291	617.1301	−1.62	20	▲	-	-	Level 3	Dimer of methyl taxiflolin and dehydroxylated methyl taxifolin 1
**M171 ^c^**	64.400	C_32_H_26_O_13_	[M − H]^−^	617.1311	617.1301	1.62	20	▲	-	-	Level 3	Dimer of methyl taxiflolin and dehydroxylated methyl taxifolin 2
**M172 ^c^**	64.925	C_32_H_26_O_13_	[M − H]^−^	617.1299	617.1301	−0.32	20	▲	-	-	Level 3	Dimer of methyl taxiflolin and dehydroxylated methyl taxifolin 3
**M173 ^c^**	65.142	C_32_H_24_O_14_	[M − H]^−^	631.1093	631.1093	0.00	21	▲	-	-	Level 3	Dimer of methyl quercetin and methyl taxifolin 1
**M174 ^c^**	66.142	C_32_H_24_O_14_	[M − H]^−^	631.1088	631.1093	−0.79	21	▲	-	-	Level 3	Dimer of methyl quercetin and methyl taxifolin 2
**M175 ^c^**	68.517	C_32_H_24_O_14_	[M − H]^−^	631.1106	631.1093	2.06	21	▲	-	-	Level 3	Dimer of methyl quercetin and methyl taxifolin 3
**M176 ^c^**	69.230	C_32_H_24_O_14_	[M − H]^−^	631.1105	631.1093	1.90	21	▲	-	-	Level 3	Dimer of methyl quercetin and methyl taxifolin 4
**M177 ^c^**	64.550	C_33_H_28_O_13_	[M − H]^−^	631.1435	631.1457	−3.49	20	▲	-	-	Level 3	Dimer of methyl taxiflolin and dehydroxylated dimethyl taxifolin 1
**M178 ^c^**	67.408	C_33_H_28_O_13_	[M − H]^−^	631.1482	631.1457	3.96	20	▲	-	-	Level 3	Dimer of methyl taxiflolin and dehydroxylated dimethyl taxifolin 2
**M179 ^c^**	67.633	C_33_H_28_O_13_	[M − H]^−^	631.1488	631.1457	4.91	20	▲	-	-	Level 3	Dimer of methyl taxiflolin and dehydroxylated dimethyl taxifolin 3
**M180 ^c^**	59.138	C_32_H_26_O_14_	[M − H]^−^	633.1257	633.1250	1.11	20	▲	-	-	Level 3	Dimer of methyl taxiflolin and methyl taxifolin 1
**M181 ^c^**	63.783	C_32_H_26_O_14_	[M − H]^−^	633.1252	633.1250	0.32	20	▲	▲	-	Level 3	Dimer of methyl taxiflolin and methyl taxifolin 2
**M182 ^c^**	69.755	C_33_H_26_O_14_	[M − H]^−^	645.1243	645.1250	−1.09	21	▲	-	-	Level 3	Dimer of methyl taxiflolin and dimethyl quercetin 1
**M183 ^c^**	71.097	C_33_H_26_O_14_	[M − H]^−^	645.1252	645.1250	0.31	21	▲	-	-	Level 3	Dimer of methyl taxiflolin and dimethyl quercetin 2
**M184 ^c^**	62.067	C_33_H_28_O_14_	[M − H]^−^	647.1432	647.1406	4.02	20	▲	-	-	Level 3	Dimer of methyl taxiflolin and dimethyl taxifolin 1
**M185 ^c^**	62.600	C_33_H_28_O_14_	[M − H]^−^	647.1420	647.1406	2.16	20	▲	-	-	Level 3	Dimer of methyl taxiflolin and dimethyl taxifolin 2
**M186 ^c^**	62.917	C_33_H_28_O_14_	[M − H]^−^	647.1419	647.1406	2.01	20	▲	-	-	Level 3	Dimer of methyl taxiflolin and dimethyl taxifolin 3
**M187 ^c^**	63.183	C_33_H_28_O_14_	[M − H]^−^	647.1406	647.1406	0.00	20	▲	-	-	Level 3	Dimer of methyl taxiflolin and dimethyl taxifolin 4
**M188 ^c^**	66.483	C_33_H_28_O_14_	[M − H]^−^	647.1434	647.1406	4.33	20	▲	-	-	Level 3	Dimer of methyl taxiflolin and dimethyl taxifolin 5
**M189 ^c^**	66.983	C_33_H_28_O_14_	[M − H]^−^	647.1405	647.1406	−0.15	20	▲	▲	-	Level 3	Dimer of methyl taxiflolin and dimethyl taxifolin 6
**M190 ^c^**	70.430	C_33_H_28_O_14_	[M − H]^−^	647.1421	647.1406	2.32	20	▲	-	-	Level 3	Dimer of methyl taxiflolin and dimethyl taxifolin 7
**M191 ^c^**	63.958	C_32_H_26_O_16_S	[M − H]^−^	697.0891	697.0869	3.16	20	▲	-	-	Level 3	Dimer of methyl taxiflolin and dehydroxylated methyl taxifolin sulphate

Abbreviations: ▲, detected; -, undetected; t_R_, retention time; ^a^ bioactivite metabolites; ^b^ known metabolites of taxifolin; ^c^ new compounds; ^d^ metabolites have specific structures. Among 191 metabolites, **M32**, **M65**, **M72**, **M75**, **M91**, **M109** were identified from the small intestine.

**Table 2:** Metabolic reactions forming 191 metabolites of taxifolin detected in rats.

		Metabolic Reaction													
No.	Amount	Phase I									Phase II				
		−H_2_O	−OH	+OH	−2H	+2H	RC	I	P		CH_3_	+SO_3_H	+GlcUA	+AA ^c^	+AM ^c^
**M1, M2**	2							▲							
**M3–M11**	9											▲			
**M12–M15**	4											▲ ^a^			
**M16**	1											▲		▲	
**M17–M25**	9												▲		
**M26–M32**	7			▲								▲	▲		
**M33–M36**	4										▲				
**M37–M46**	10										▲	▲			
**M47–M55**	9										▲		▲		
**M56, M57**	2										▲	▲	▲		
**M58, M59**	2										▲			▲	
**M60–M63**	4			▲							▲	▲			
**M64, M65**	2			▲ ^a^							▲				
**M66–M69**	4			▲ ^a^							▲		▲		
**M70**	1				▲										
**M71–M75**	5				▲							▲			
**M76**	1				▲								▲		
**M77–M79**	3				▲						▲				
**M80**	1				▲						▲	▲			
**M81–M84**	4				▲						▲	▲			
**M85**	1				▲						▲	▲ ^a^	▲		
**M86, M87**	2				▲						▲		▲		
**M88–M91**	4				▲						▲	▲	▲		
**M92–M94**	3			▲	▲							▲			
**M95, M96**	2			▲	▲								▲		
**M97–M100**	4			▲	▲						▲	▲			
**M101–M103**	3			▲	▲						▲		▲		
**M104, M105**	2		▲												
**M106–M109**	4		▲									▲			
**M110–M112**	3		▲										▲		
**M113–M115**	3	▲											▲		
**M116**	1	▲									▲		▲		
**M117**	1					▲									
**M118**	1					▲					▲				
**M119–M121**	3					▲						▲			
**M122, M123, M131, M136, M142, M143**	6						▲					▲			
**M124, M125, M128–M130, M132–M135,M137, M138, 144-M149, M154–M159**	23						▲					▲			
**M126, M127, M150, M151**	4						▲						▲		
**M139–M141**	3						▲								▲
**M152, M153**	2						▲					▲	▲		
**M160**	1		▲						▲		▲				
**M161–M162**	2								▲		▲				
**M163, M180, M181**	3								▲		▲ ^a^				
**M164–M166**	3								▲		▲	▲			
**M167–M169**	3								▲		▲ ^a^	▲			
**M170–M172**	3		▲						▲		▲ ^a^				
**M173–M176**	4				▲				▲		▲ ^a^				
**M177–M179**	3		▲						▲		▲ ^b^				
**M182, M183**	2				▲				▲		▲ ^b^				
**M184–M190**	7								▲		▲ ^b^				
**M191**	1		▲						▲		▲ ^a^				
**Sum**	191	4	17	29	40	5	38	2	32		93	103	57	3	3

Abbreviations: −H_2_O, dehydration; −OH, dehydroxylation; +OH, hydroxylation; −2H, dehydrogenation; +2H, hydrogenation; RC, ring cleavage; I, isomerization; P, polymerization; +CH_3_, methylation; +SO_3_H, sulphation; +GlcUA, glucuronidation; +AA, amino acid conjugation; +AM, acetylamination. ^a^ metabolic reaction repeated two times; ^b^ metabolic reaction repeated three times; ^c^ new metabolic reaction. ▲, denoting the metabolic reaction is detected.

**Table 3:** Distribution of taxifolin and its 46 metabolites in rats.

No.	Heart	Liver	Spleen	Lung	Kindey	Brain	Stomach	Intestine
**TAX**	▲	▲	▲	▲	▲	▲	▲	▲
**M2**	-	▲	-	▲	▲	▲	▲	▲
**M5**	-	▲	-	-	▲	-	▲	-
**M7**	-	-	-	-	-	-	-	▲
**M11**	▲	▲	▲	▲	▲	-	▲	▲
**M18**	▲	▲	▲	▲	▲	-	▲	▲
**M19**	▲	▲	▲	▲	▲	-	▲	▲
**M20**	-	-	▲	▲	▲	-	▲	▲
**M21**	-	▲	▲	▲	▲	-	▲	▲
**M22**	▲	-	-	-	-	-	-	-
**M23**	-	▲	-	-	▲	-	▲	▲
**M24**	-	▲	-	-	-	-	-	▲
**M25**	-	▲	-	-	▲	-	▲	▲
**M28**	-	-	-	-	▲	-	▲	-
**M29**	-	-	-	-	-	-	-	▲
**M30**	-	-	-	-	▲	-	▲	▲
**M31**	-	-	-	-	-	-	▲	▲
**M32**	-	▲	-	-	▲	-	-	▲
**M33**	▲	▲	▲	▲	▲	▲	▲	▲
**M34**	-	▲	▲	▲	▲	▲	▲	▲
**M35**	-	▲	-	-	▲	-	▲	▲
**M36**	-	-	-	-	-	-	▲	-
**M42**	-	▲	-	-	▲	-	▲	-
**M43**	▲	-	▲	▲	-	-	-	▲
**M44**	-	▲	-	-	▲	-	▲	-
**M45**	-	▲	-	-	▲	-	▲	▲
**M48**	-	▲	-	▲	▲	-	▲	▲
**M49**	▲	▲	▲	▲	▲	-	▲	▲
**M50**	-	▲	▲	▲	▲	-	▲	▲
**M51**	-	-	-	-	-	-	-	▲
**M52**	-	▲	-	-	▲	-	▲	▲
**M65**	-	-	-	-	-	-	▲	▲
**M70**	-	-	-	-	-	-	▲	▲
**M72**	-	-	-	-	▲	-	-	▲
**M75**	-	-	-	-	▲	-	-	▲
**M80**	-	-	-	-	▲	-	-	▲
**M84**	-	▲	-	-	▲	-	▲	▲
**M86**	-	-	-	-	▲	-	-	-
**M87**	-	-	-	-	-	-	-	▲
**M91**	-	-	-	-	-	-	-	▲
**M105**	-	▲	-	-	▲	-	▲	▲
**M109**	-	-	-	-	▲	-	-	▲
**M118**	-	-	-	-	-	-	-	▲
**M150**	-	-	-	-	▲	-	-	-
**M151**	-	-	-	-	▲	-	-	-
**M161**	-	-	-	-	-	-	▲	-
**M162**	-	-	-	-	-	-	▲	-
**SUM**	7	22	10	12	31	3	29	35

Abbreviations: ▲, detected; -, undetected.

**Table 4:** The common fragments (in red) and their related metabolites.

Fragment No.	Count of Metabolites	The Structures of Metabolites	Bioactive Metabolites and Related Pharmacological Effects
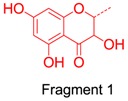	4	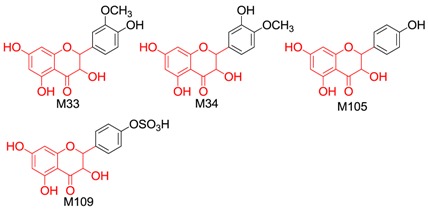	**M105** (one metabolite) Antioxidant, Anti-inflammatory, Antitumor, Antimicrobial, Xanthine oxidase inhibitor
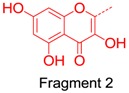	6	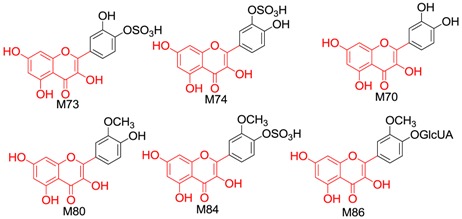	**M70**, **M73**, **M74**, **M80** (four metabolites) Antioxidant, Anti-inflammatory, Antitumor, Cardioprotective, Antidiabetic, Antimicrobial, Antiviral, Hepatoprotective, Prevention of Alzheimer disease, Immunoregulatory, Xanthine oxidase inhibitor, Neuroprotective
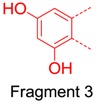	18	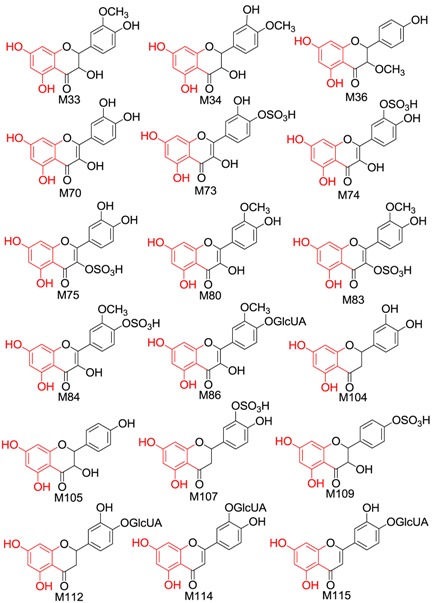	**M70**, **M73**, **M74**, **M75**, **M80**, **M83**, **M104**, **M105** (eight metabolites) Antioxidant, Anti-inflammatory, Antitumor, Cardioprotective, Antidiabetic, Antimicrobial, Antiviral, Hepatoprotective, Prevention of Alzheimer disease, Immunoregulatory, Xanthine oxidase inhibitor, Neuroprotective
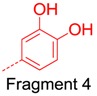	14	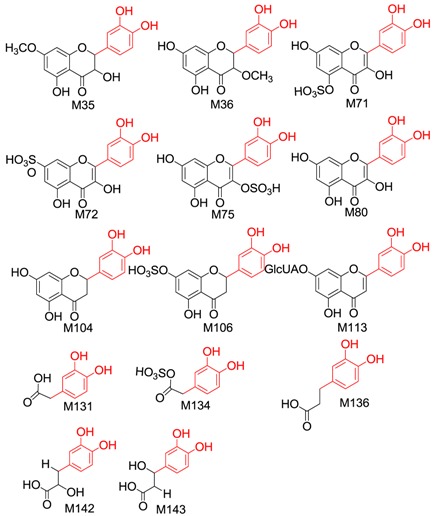	**M75**, **M80**, **M104**, **M113**, **M131**, **M136** (six metabolites) Antioxidant, Anti-inflammatory, Antitumor, Cardioprotective, Antidiabetic, Antimicrobial, Antiviral, Hepatoprotective, Prevention of Alzheimer disease, Immunoregulatory, Xanthine oxidase inhibitor, Neuroprotective

## Supplementary Materials

Supplementary materials can be accessed at: http://www.mdpi.com/1420-3049/21/9/1209/s1.
