# Supplementary material for: Detection of 191 Taxifolin Metabolites and Their Distribution in Rats Using HPLC-ESI-IT-TOF-MSn
Source: Molecules. 2016 Sep 13;21(9):1209. doi: 10.3390/molecules21091209 (PMC6273498; doi:10.3390/molecules21091209)
Supplement: Supplementary file 1 [file molecules-21-01209-s001.pdf]

Supplementary Materials: Detection of 191 Taxifolin Metabolites and Their Distribution in Rats Using HPLC-ESI-IT-TOF-MS^n

Ping Yang, Feng Xu, Hong-Fu Li, Yi-Wang, Feng-Chun Li, Ming-Ying Shang, Guang-Xue Liu, Xuan Wang, Shao-Qing Cai

Table S1. Retention time (t_R), HRMS^n data, molecular formula and identification of taxifolin and its metabolites in rats by HPLC-DAD-ESI-IT-TOF-MS^n.

	t_R(min)	Formula	Meas. *m/z*	Identification	Other Ions	HRMS^n(Characteristic Fragment Ions and Their Relative Abundance)
**TAX**	41.023	$C_{15}H_{12}O_7$	303.0521	Taxifolin(parent compound)	$[M + H]^+$: 305.0621	MS^2[303.0520]: 285.0407(100.0), 241.0524(26.25), 217.0529(3.33), 177.0253(12.87), 175.0440(15.76), 125.0290(8.65); MS^3[285.0406]: 241.0532(49.03), 213.0566(14.55), 199.0449(41.19), 175.0424(100.0)
**M1**	40.567	$C_{15}H_{12}O_7$	303.0502	Taxifolin isomer 1	$[M + H]^+$: 305.0624<br>$[2M - H]^-$: 607.1132	MS^2[303.0490]: 285.0371(100.0), 241.0504(18.49), 177.0236(11.8), 175.0525(10.39), 125.0286(8.51); MS^3[285.0371]: 241.0589(100), 243.0344(35.54), 175.0387(94.08)
**M2**	42.883	$C_{15}H_{12}O_7$	303.0517	Taxifolin isomer 2	$[M + H]^+$: 305.0618<br>$[2M - H]^-$: 607.1132	MS^2[303.0517]: 285.0425(100.0), 241.0521(33.23), 177.0302(5.75), 175.0444(9.38), 125.0258(7.83); MS^3[285.0423]: 241.0454(60.16), 175.0465(100.0)
**M3**	21.517	$C_{15}H_{12}O_{10}S$	383.0080	Taxifolin sulphate 1		MS^2[383.0078]: 303.0514(5.67), 285.0383(100.0), 241.05(17.89), 175.0459(23.67), 125.0274(3.4); MS^3[286.0426]: 241.0516(100.0), 217.0579(31.44), 199.0418(46.01), 175.0395(85.87)
**M4**	31.242	$C_{15}H_{12}O_{10}S$	383.0089	Taxifolin sulphate 2		MS^2[383.0089]: 303.049(12.71), 285.0384(100.0), 241.0512(14.26), 175.0457(10.69), 125.0288(1.69); MS^3[285.0383]: 241.0529(65.85), 217.0571(27.1), 199.0452(41.93), 175.0442(100.0)
**M5**	32.145	$C_{15}H_{12}O_{10}S$	383.0073	Taxifolin sulphate 3	$[M + HCOOH - H]^-$: 429.0178	MS^2[383.0073]: 303.05(11.06), 285.0394(100.0), 243.0294(1.76), 241.0481(11.2), 175.0424(4.11), 125.0317(1.39); MS^3[285.0393]: 243.0196(12.25), 241.0527(24.74), 217.0512(18.5), 175.0416(100.0)
**M6**	35.292	$C_{15}H_{12}O_{10}S$	383.0078	Taxifolin sulphate 4		MS^2[383.0077]: 303.0496(9.45), 285.0374(100.0), 241.0514(17.35), 175.0437(16.0), 125.0312(4.14); MS^3[285.0373]: 241.0568(16.81), 213.0608(16.81), 199.0371(39.63), 175.0396(100.0)
**M7**	36.717	$C_{15}H_{12}O_{10}S$	383.0079	Taxifolin sulphate 5		MS^2[383.0079]: 303.0578(0.69), 285.0375(100.0), 241.0505(16.27), 217.048(5.3), 175.0531(9.78)
**M8**	37.925	$C_{15}H_{12}O_{10}S$	383.0070	Taxifolin sulphate 6	$[M + HCOOH - H]^-$: 429.0147	MS^2[383.007]: 303.0488(11.05), 285.0375(100.0), 241.0503(22.65), 177.0265(10.85), 175.039(17.28), 125.0286(1.0); MS^3[285.0374]: 217.0466(100.0), 199.0392(36.07), 195.0459(43.17)
**M9**	39.375	$C_{15}H_{12}O_{10}S$	383.0087	Taxifolin sulphate 7	$[M + H]^+$: 385.0219	MS^2[383.0086]: 303.0488(15.4), 285.0392(100.0), 241.0516(20.79), 199.0432(5.02), 175.0428(14.67); MS^3[285.0392]: 243.0331(14.5), 241.0508(44.76), 217.0479(22.02), 199.0431(45.3), 175.0424(100.0)
**M10**	41.192	$C_{15}H_{12}O_{10}S$	383.0086	Taxifolin sulphate 8	$[M + H]^+$: 385.0222	MS^2[383.0089]: 303.0507(11.96), 285.0393(100.0), 241.0511(20.93), 175.0435(15.46), 125.0292(2.32); MS^3[285.0392]: 241.0478(47.49), 199.0426(61.46), 175.0416(100.0), 129.0387(5.86), 125.0392(1.41)
**M11**	43.000	$C_{15}H_{12}O_{10}S$	383.0082	Taxifolin sulphate 9	$[M + H]^+$: 385.0227	MS^2[383.0082]: 303.0503(9.17), 285.0388(100.0), 241.0513(21.31), 177.0215(14.8), 175.0423(16.18); MS^3[285.0387]:217.0557(20.19),213.0546(17.67),199.042(57.12), 175.0414(100.0)

	t_R(min)	Formula	Meas. *m/z*	Identification	Other Ions	HRMS^n(Characteristic Fragment Ions and Their Relative Abundance)
**M12**	24.592	$C_{15}H_{12}O_{13}S_2$	462.9644	Taxifolin disulphate 1	[M + CH_3COOH − H]$^-$: 522.9886	MS^2[462.9692]: 383.0074(100.0),303.0496(3.04),285.0342(1.52); MS^3[383.0074]: 285.0394(100.0), 241.0546(11.61), 177.0255(2.58), 175.0465(17.08), 151.012(8.59)
**M13**	27.458	$C_{15}H_{12}O_{13}S_2$	462.9670	Taxifolin disulphate 2	[M + $2H_2O$ − H]$^-$: 498.9592	MS^2[462.9673]: 383.0077(100.0), 303.0496(2.89), 285.0446(8.47), 230.9773(4.65); MS^3[383.0077]: 285.0418(100.0), 241.0527(5.91), 217.0476(11.25), 177.0308(10.04), 175.0392(10.13)
**M14**	31.075	$C_{15}H_{12}O_{13}S_2$	462.9639	Taxifolin disulphate 3	[M + HCOOH − H]$^-$: 509.0061 [M + CH_3COOH − H]$^-$: 523.0319	MS^2[462.9642]: 383.0077(100.0),303.0496(1.14),231.0069(1.69); MS^3[383.0076]: 303.0496(7.04), 285.0395(100.0), 241.0454(12.38), 175.0215(3.59), 125.0286(3.59)
**M15**	39.767	$C_{15}H_{12}O_{13}S_2$	462.9656	Taxifolin disulphate 4		MS^2[462.9657]: 383.0065(100.0), 303.061(3.2), 285.0368(5.53); MS^3[383.0065]: 303.0496(5.89), 285.0398(100.0), 241.0552(38.26), 175.0473(22.51), 125.0244(4.89)
**M16**	16.252	$C_{20}H_{19}NO_{13}S$	512.0509	Taxifolin sulphate and pyroglutamic acid conjugate	[M + H]$^+$: 514.1463	MS^2[512.054]: 415.0651(28.19), 397.0505(22.72), 383.0083(100.0), 303.0502(28.19), 285.0358(71.79), 241.059(13.11), 175.0446(13.8); MS^3[383.0083]: 353.2434(18.05), 285.0401(100.0), 259.0791(22.65), 241.0517(31.86), 177.0129(18.05)
**M17**	15.408	$C_{21}H_{20}O_{13}$	479.0834	Taxifolin glucuronide 1	[M + CH_3COOH − H]$^-$:539.1166	MS^2[479.0892]: 303.0448(21.29), 285.0386(100.0), 241.0601(42.59), 231.4908(15.29), 175.0403(12.24)
**M18**	18.637	$C_{21}H_{20}O_{13}$	479.0850	Taxifolin glucuronide 2	[M + HCOOH − H]$^-$: 525.0929	MS^2[479.0912]: 303.0534(24.22), 285.0401(100.0), 241.0498(10.69), 177.0213(11.32), 175.0409(10.89); MS^3[285.0400]: 243.0255(41.83), 197.0614(100.0)
**M19**	20.253	$C_{21}H_{20}O_{13}$	479.0847	Taxifolin glucuronide 3	[M + H]$^+$: 481.0965	MS^2[479.0847]: 303.0472(17.51), 285.038(100.0), 241.0508(21.38), 177.0203(16.27), 175.0521(2.08); MS^3[285.038]: 241.0527(14.07), 239.0353(14.07), 217.0466(21.1), 199.0464(100.0), 175.0502(45.58)
**M20**	21.370	$C_{21}H_{20}O_{13}$	479.0843	Taxifolin glucuronide 4	[M + H]$^+$: 481.0949	MS^2[479.0877]: 303.049(32.82), 285.0379(100.0), 241.0515(13.88), 177.0205(17.26), 175.0412(6.27); MS^3[285.0378]: 241.0513(72.43), 199.0414(100.0), 175.0465(58.38)
**M21**	22.267	$C_{21}H_{20}O_{13}$	479.0838	Taxifolin glucuronide 5	[M + H] $^+$: 481.1133	MS^2[479.0863]: 303.047(28.1), 285.0392(100.0), 241.0507(10.52), 217.0466(4.3), 177.0242(1.54), 175.039(3.09)
**M22**	22.587	$C_{21}H_{20}O_{13}$	479.0847	Taxifolin glucuronide 6	[M + H]$^+$: 481.0898	MS^2[479.0865]: 383.0002(2.13), 303.053(27.43), 285.037(100.0), 241.0527(6.27), 177.0297(11.87), 175.0411(2.48); MS^3[285.0368]: 217.0425(100.0), 175.0465(85.25), 81.139(57.92)
**M23**	31.862	$C_{21}H_{20}O_{13}$	479.0830	Taxifolin glucuronide 7	[M + H]$^+$: 481.0848	MS^2[479.0864]: 285.0423(100.0), 259.0577(4.72), 243.0256(5.84), 241.0704(22.56), 177.0255(4.72), 175.0443(34.32); MS^3[285.0422]: 175.0415(100.0), 129.0419(80.0)
**M24**	34.742	$C_{21}H_{20}O_{13}$	479.0832	Taxifolin glucuronide 8	[M + H]$^+$: 481.0872	N
**M25**	37.267	$C_{21}H_{20}O_{13}$	479.0834	Taxifolin glucuronide 9	[M + H]$^+$: 481.0972	MS^2[479.0865]: 303.0551(3.1), 285.0393(100.0), 259.0654(6.68), 241.0511(21.39), 177.0192(8.25), 175.0421(14.67); MS^3[285.0393]: 243.0492(50.0), 241.0601(100.0), 217.0884(74.51), 175.0516(62.25)

	t_R(min)	Formula	Meas. *m*/*z*	Identification	Other Ions	HRMS^n(Characteristic Fragment Ions and Their Relative Abundance)
**M26**	13.888	$C_{21}H_{20}O_{16}S$	559.0388	Taxifolin glucuronide sulphate 1	[M + K]$^+$: 599.0232	MS^2[559.0472]: 383.0082(100.0), 303.0551(29.1), 285.0448(86.56), 241.0454(19.52), 177.0192(19.52)
**M27**	16.703	$C_{21}H_{20}O_{16}S$	559.0423	Taxifolin glucuronide sulphate 2	[M + 2H_2O − H]$^-$: 595.0978	MS^2[559.0425]: 479.0768(3.82), 383.006(88.44), 303.0496(38.47), 285.0408(100.0), 241.0601(5.76)
**M28**	19.928	$C_{21}H_{20}O_{16}S$	559.0406	Taxifolin glucuronide sulphate 3	[M + 2H_2O − H]$^-$: 595.0895	MS^2[559.045]: 479.0975(1.96), 383.0075(93.41), 303.0478(50.31), 285.0385(100.0), 241.059(10.52), 177.0318(4.95)
**M29**	21.812	$C_{21}H_{20}O_{16}S$	559.0411	Taxifolin glucuronide sulphate 4	[M + HCOOH − H]$^-$: 605.0349	MS^2[559.0411]: 479.0872(2.9), 383.008(100.0), 303.0481(49.6), 285.0372(88.61), 241.0512(15.36), 175.0429(2.03); MS^3[383.008]: 285.043(100.0), 257.0496(4.04), 241.0468(15.28), 199.0472(6.1), 177.0235(31.64), 175.0434(17.22)
**M30**	23.087	$C_{21}H_{20}O_{16}S$	559.0418	Taxifolin glucuronide sulphate 5		MS^2[559.0429]: 383.0072(100.0), 303.0493(18.56), 285.0375(89.58), 241.0509(2.6), 175.0716(1.96); MS^3[383.007]: 285.0486(100.0), 243.027(7.03), 177.0507(10.55), 151.0586(10.55)
**M31**	24.762	$C_{21}H_{20}O_{16}S$	559.0425	Taxifolin glucuronide sulphate 6	[M + CH_3HCOOH − H]$^-$: 619.0803[M + K]$^+$: 599.0128	MS^2[559.0428]: 383.0062(100.0), 303.0488(39.46), 285.0367(91.03), 243.0393(1.65), 241.0528(18.71), 175.0358(6.0)
**M32**	25.797	$C_{21}H_{20}O_{16}S$	559.0411	Taxifolin glucuronide sulphate 7		MS^2[559.0411]: 383.0068(100.0), 303.0465(23.09), 285.0387(71.61), 241.0502(10.1), 177.02(1.82), 175.0348(1.21)
**M33**	50.292	$C_{16}H_{14}O_7$	317.0675	3′-*O*-Methyltaxifolin	[M + H]$^+$: 319.0782	MS^2[317.0674]: 299.0567(4.3), 289.0718(100.0), 275.0559(1.81), 273.0753(6.36), 231.0668(7.17), 230.0596(6.54), 152.0141(7.15), 125.0276(6.28); MS^3[289.0717]: 275.0491(20.25), 231.0637(16.06), 230.0642(38.12), 152.014(100.0), 125.0317(17.29)
**M34**	51.350	$C_{16}H_{14}O_7$	317.0673	4′-*O*-Methyltaxifolin	[M + H]$^+$: 319.0791	MS^2[317.2205]: 299.0586(4.08), 289.072(100.0), 275.056(3.09), 230.0592(6.3), 152.0147(7.56); MS^3[289.0718]: 275.0521(13.72), 274.0503(24.58), 231.0648(15.28), 152.0159(100.0), 125.0363(16.05)
**M35**	52.875	$C_{16}H_{14}O_7$	317.0667	7-*O*-Methyltaxifolin	[M + H]$^+$: 319.0786	MS^2[317.0665]: 299.0567(16.86), 289.0698(51.55), 231.0592(11.55), 230.0602(33.52), 216.0428(20.03), 214.0649(11.55), 166.0254(6.53), 165.0259(13.14), 152.0132(100.0), 125.0282(75.55); MS^3[274.046]: 152.0121(100.0), 125.018(9.13)
**M36**	53.592	$C_{16}H_{14}O_7$	317.0660	3-*O*-Methyltaxifolin	[M + H]$^+$: 319.0842	MS^2[317.0645]: 299.0606(46.64), 289.0714(65.79), 284.0328(29.14), 274.0481(51.89), 240.0466(20.24), 230.0559(27.86), 152.01(100.0), 125.0286(53.06); MS^3 [289.0713]: 275.4196(66.23), 230.0723(100.0), 152.0087(100.0)
**M37**	28.575	$C_{16}H_{14}O_{10}S$	397.0243	Methyl taxifolin sulphate 1	[M + H]$^+$: 399.0333	MS^2[397.0245]: 317.0646(95.45), 299.054(1.59), 289.0715(100.0), 273.0751(8.37), 258.0557(2.21), 231.0691(3.5); MS^3[289.0714]: 274.0383(21.25), 229.0535(7.08), 179.0726(7.08), 152.0171(100.0), 125.0233(7.08)
**M38**	33.942	$C_{16}H_{14}O_{10}S$	397.0240	Methyl taxifolin sulphate 2	[M + H]$^+$: 399.0333	MS^2[397.0241]: 317.0646(97.68), 299.0554(2.38), 289.0703(100.0), 274.0471(3.37), 273.0752(7.19), 231.0632(5.98); MS^3[289.0703]:

	t_R(min)	Formula	Meas. *m*/*z*	Identification	Other Ions	HRMS^n(Characteristic Fragment Ions and Their Relative Abundance)
						275.0635(4.97), 274.0473(5.81), 230.0636(4.13), 152.0193(100.0), 125.0286(3.29), 123.0037(3.29)
**M39**	34.420	$C_{16}H_{14}O_{10}S$	397.0247	Methyl taxifolin sulphate 3		MS^2[397.0248]: 317.0651(100.0), 289.0700(100.0), 273.0809(4.17), 258.0527(2.09), 231.0673(5.55)
**M40**	35.858	$C_{16}H_{14}O_{10}S$	397.0253	Methyl taxifolin sulphate 4	[M + HCOOH − H]$^-$: 443.0598	MS^2[397.0256]: 317.066(100.0), 299.0496(1.27), 289.0717(83.29), 273.0771(8.94), 231.0657(3.43), 152.0195(1.27); MS^3[317.0659]: 299.0501(10.55), 289.0722(100.0), 274.0446(21.01), 137.0202(8.44), 125.0339(8.44)
**M41**	38.092	$C_{16}H_{14}O_{10}S$	397.0241	Methyl taxifolin sulphate 5	[M + H]$^+$: 399.0617	MS^2[397.0242]: 317.0653(100.0), 299.0576(4.32), 289.0717(97.36), 258.0519(3.75), 231.0677(5.08), 152.012(0.52); MS^3[317.0652]: 299.0522(2.69), 289.0715(100.0), 275.0601(2.69), 274.0487(19.53), 152.014(11.28), 125.0291(10.3)
**M42**	40.283	$C_{16}H_{14}O_{10}S$	397.0233	Methyl taxifolin sulphate 6	[M + H]$^+$: 399.0428	MS^2[397.0235]: 317.0646(95.02), 299.0567(1.12), 289.0713(100.0), 273.0782(10.29), 231.0645(4.86); MS^3[289.0713]: 230.0637(28.09), 213.0677(22.55), 152.0048(100.0)
**M43**	41.817	$C_{16}H_{14}O_{10}S$	397.0241	Methyl taxifolin sulphate 7	[M + H]$^+$: 399.0333	MS^2[397.0244]: 317.066(76.6), 299.0485(1.14), 289.0712(100.0), 274.049(7.7), 273.0763(13.71), 231.0645(7.5), 230.0565(7.61), 152.0178(5.16); MS^3[289.0711]: 274.0383(10.84), 230.0651(7.13), 152.0105(100.0)
**M44**	42.717	$C_{16}H_{14}O_{10}S$	397.0230	Methyl taxifolin sulphate 8	[M + H]$^+$: 399.0333	MS^2[397.0232]: 317.0643(86.98), 299.0558(2.81), 289.0705(100.0), 274.0424(2.81), 273.0753(9.12), 258.0517(4.65), 231.0625(2.13), 152.0327(2.56); MS^3[289.0704]: 274.054(11.83), 230.0538(20.75), 152.0107(100.0), 125.0498(11.83)
**M45**	43.600	$C_{16}H_{14}O_{10}S$	397.0235	Methyl taxifolin sulphate 9	[M + H]$^+$: 399.0523	MS^2[397.0236]: 317.0638(81.59), 299.0546(4.06), 289.0698(100.0), 273.0764(7.76), 258.0515(4.4), 231.0744(6.64), 152.0139(3.27); MS^3[289.0697]: 274.0477(8.95), 258.0588(4.49), 230.0579(28.57), 152.0215(100.0), 125.0286(3.62)
**M46**	45.558	$C_{16}H_{14}O_{10}S$	397.0238	Methyl taxifolin sulphate 10	[M + CH_3HCOOH − H]$^-$: 457.0121	MS^2[397.024]: 317.0652(97.99), 299.0532(38.06), 289.072(76.49), 274.0481(18.77), 258.0491(13.35), 245.0114(100.0), 165.0444(26.72), 152.0134(4.48); MS^3 [245.0115]: 165.0584(100.0), 150.3843(18.37), 150.0244(27.21)
**M47**	23.520	$C_{22}H_{22}O_{13}$	493.0973	Methyl taxifolin glucuronide 1	[M + H]$^+$: 495.1021	MS^2[493.1057]: 317.0651(100.0), 299.0567(1.28), 289.0713(65.83), 273.0804(5.82), 231.0645(3.87), 230.0608(4.52); MS^3[317.065]: 289.0734(100.0), 274.0446(29.98)
**M48**	25.212	$C_{22}H_{22}O_{13}$	493.1012	Methyl taxifolin glucuronide 2	[M + H]$^+$: 495.1153	MS^2[493.1014]: 317.0644(100.0), 299.0526(2.98), 289.0707(86.87), 273.075(2.96), 229.0893(2.22), 152.0309(1.86); MS^3[317.0642]: 289.072(100.0), 230.0579(20.19), 125.0286(29.9)
**M49**	26.687	$C_{22}H_{22}O_{13}$	493.1012	Methyl taxifolin glucuronide 3	[M + H]$^+$: 495.1101	MS^2[493.1032]: 317.0646(91.34), 299.064(2.65), 289.0708(100.0), 274.0553(9.18), 273.0792(5.73), 258.0537(3.78), 231.064(5.88),

	t_R(min)	Formula	Meas. *m/z*	Identification	Other Ions	HRMS^n(Characteristic Fragment Ions and Their Relative Abundance)
						230.0609(4.53); MS^3[289.0708]: 274.054(11.46), 230.0493(9.55), 152.012(100.0), 125.0392(11.46)
**M50**	30.383	$C_{22}H_{22}O_{13}$	493.1012	Methyl taxifolin glucuronide 4	[M + H]$^+$: 495.1335	MS^2[493.1028]: 317.0648(100.0), 299.0595(16.32), 289.0707(98.02), 274.054(4.15), 273.0703(10.31), 231.0583(2.41); MS^3[317.0646]: 289.0711(100.0), 258.0527(27.04), 231.0645(27.04)
**M51**	33.395	$C_{22}H_{22}O_{13}$	493.1007	Methyl taxifolin glucuronide 5	[M + H]$^+$: 495.0873	MS^2[493.1011]: 475.0909(17.95), 359.0714(7.65), 331.0818(19.27), 317.0646(41.77), 299.0548(39.97), 289.0703(100.0),284.0371(10.41), 274.0462(7.17); MS^3[289.0702]: 152.0106(100.0)
**M52**	35.692	$C_{22}H_{22}O_{13}$	493.0998	Methyl taxifolin glucuronide 6	[M + H]$^+$: 495.1161	MS^2[493.1046]: 317.0645(55.37), 299.0525(9.98), 289.0695(100.0), 273.0709(5.43), 258.0527(4.2), 230.0615(5.98), 188.0479(5.63); MS^3[289.0694]: 274.0383(57.92), 243.0639(57.92), 223.8944(57.92), 152.0095(100.0)
**M53**	36.025	$C_{22}H_{22}O_{13}$	493.1004	Methyl taxifolin glucuronide 7	[M + H]$^+$: 495.1375	MS^2[493.1006]: 359.0698(1.25), 331.0861(2.45), 317.0653(60.45), 299.0546(18.79), 289.0693(100.0), 284.0289(2.15), 275.0426(1.86), 273.0803(5.52), 258.0546(8.17), 152.0029(1.25)
**M54**	37.600	$C_{22}H_{22}O_{13}$	493.0998	Methyl taxifolin glucuronide 8	[M + H]$^+$: 495.1176	MS^2[493.1001]: 389.0919(9.65), 361.1005(7.22), 359.0722(100.0), 341.0611(8.43), 331.0791(65.09), 317.0686(55.48), 289.0682(93.96), 231.0711(13.26)
**M55**	42.375	$C_{22}H_{22}O_{13}$	493.1008	Methyl taxifolin glucuronide 9	[M + H]$^+$: 495.1031	N
**M56**	34.742	$C_{22}H_{22}O_{16}S$	573.0560	Methyl taxifolin glucuronide sulphate 1		MS^2[573.0562]: 493.0971(79.52), 373.0538(100.0), 345.0588(20.05), 327.0453(3.96), 317.064(2.82); MS^3[373.0538]: 345.0626(45.61), 327.0478(45.69), 317.0623(100.0), 301.0697(14.01), 176.994(11.94), 165.0203(8.11), 137.0258(11.94)
**M57**	37.158	$C_{22}H_{22}O_{16}S$	573.0533	Methyl taxifolin glucuronide sulphate 2		MS^2[573.0613]: 493.0986(95.06), 373.0576(100.0), 345.0592(27.89), 327.0626(15.21), 317.0625(4.76), 301.0744(3.57); MS^3[373.0577]: 345.068(66.23), 317.0899(66.23), 193.0275(100.0)
**M58**	16.490	$C_{21}H_{21}NO_{10}$	446.1107	Methyl taxifolin pyroglutamic acid conjugate 1	[M + H]$^+$: 448.1151	N
**M59**	18.483	$C_{21}H_{21}NO_{10}$	446.1086	Methyl taxifolin pyroglutamic acid conjugate 2	[M + H]$^+$:448.1187	MS^2[446.1086]: 429.1075(3.05), 317.0688(27.21), 289.0729(100.0), 273.075(4.57)
**M60**	37.848	$C_{16}H_{14}O_{11}S$	413.0200	Hydroxylated methyl taxifolin sulphate 1		MS^2[413.02]: 333.06(66.16), 316.0592(6.39), 315.0486(100.0), 285.0382(8.51), 283.0225(36.67), 255.0281(19.97); MS^3[315.0486]: 285.0358(8.8), 284.0278(14.05), 283.0213(66.56), 256.0334(7.09), 255.03(100.0)
**M61**	41.943	$C_{16}H_{14}O_{11}S$	413.0175	Hydroxylated methyl taxifolin sulphate 2		N
**M62**	42.375	$C_{16}H_{14}O_{11}S$	413.0198	Hydroxylated methyl taxifolin sulphate 3		MS^2[413.0197]: 333.0578(4.33), 315.0468(100.0), 305.0684(18.88), 283.0237(25.58), 255.0298(6.5), 211.0392(7.26), 181.0156(5.93), 177.025(20.05), 155.0399(18.85); MS^3[315.0467]: 300.0565(21.22),

	t_R(min)	Formula	Meas. *m*/*z*	Identification	Other Ions	HRMS^n(Characteristic Fragment Ions and Their Relative Abundance)
						283.0265(68.37), 255.0342(94.29), 239.0353(21.22), 211.0425(100.0), 139.0619(21.22)
**M63**	42.660	$C_{16}H_{14}O_{11}S$	413.0191	Hydroxylated methyl taxifolin sulphate 4		MS^2[413.0222]: 333.0634(6.35), 315.0531(100.0), 305.0708(9.05), 290.0406(26.17), 283.028(12.67), 211.0336(3.65), 183.0292(5.47), 181.0168(7.23), 177.0246(6.35), 155.042(9.12), 153.0262(3.65), 149.0285(5.47), 139.0061(3.65)
**M64**	55.808	$C_{16}H_{14}O_9$	349.0580	Methylated and dihydroxylated taxifolin 1	$[M + H]^+$: 351.1037	MS^2[349.0579]: 331.0377(45.13), 313.0387(5.7), 285.0422(5.7), 269.043(100.0), 209.0125(38.12), 169.0145(15.5), 165.0251(7.12), 151.0105(97.97), 149.0228(5.7)
**M65**	56.608	$C_{16}H_{14}O_9$	349.0551	Methylated and dihydroxylated taxifolin 2		MS^2[349.0617]: 331.0549(38.24), 287.0453(13.9), 227.0237(13.9), 209.0081(100.0), 181.0113(13.9), 169.0177(31.02), 161.0308(13.9), 151.0035(79.28), 139.0403(13.9), 121.0273(20.86)
**M66**	17.170	$C_{22}H_{22}O_{15}$	525.0865	Methylated and dihydroxylated taxifolin glucuronide 1	$[M + K]^+$: 565.0779	MS^2[525.0866]: 349.0552(17.05), 332.0346(4.38), 331.0427(88.77), 301.0334(10.64), 300.0237(44.17), 299.0195(100.0), 273.0359(4.38), 272.025(6.45), 271.0206(79.82), 227.037(8.53), 211.052(6.45)
**M67**	17.887	$C_{22}H_{22}O_{15}$	525.0908	Methylated and dihydroxylated taxifolin glucuronide 2	$[M + K]^+$: 565.0803	MS^2[525.0934]: 349.0525(19.31), 331.0425(100.0), 317.0224(1.95), 301.0306(19.8), 300.0266(31.91), 299.0183(71.56), 272.0197(3.87), 271.0237(65.13), 255.0191(2.89), 227.0323(2.91); MS^3[331.0424]: 301.0317(29.61), 300.0237(58.77), 298.9748(35.54), 271.0223(100.0)
**M68**	18.637	$C_{22}H_{22}O_{15}$	525.0890	Methylated and dihydroxylated taxifolin glucuronide 3	$[M + K]^+$: 565.0891	MS^2[525.093]: 349.0508(23.98), 331.0433(100.0), 301.0334(22.62), 300.0256(49.86), 299.0186(62.42), 271.0248(52.95); MS^3[331.0432]: 300.0247(36.78), 299.0151(20.83), 271.0269(100.0), 149.0632(6.38)
**M69**	19.178	$C_{22}H_{22}O_{15}$	525.0911	Methylated and dihydroxylated taxifolin glucuronide 4	$[M + K]^+$: 565.0762	MS^2[525.0942]: 349.0541(37.32), 331.043(100.0), 301.0289(12.54), 300.0267(46.8), 299.0167(75.71), 271.0242(67.54), 255.0302(5.14); MS^3[331.0428]: 301.0307(9.56), 300.0309(25.46), 299.0325(59.2), 271.0214(100.0), 179.0156(6.41)
**M70**	58.150	$C_{15}H_{10}O_7$	301.0350	Quercetin	$[M + H]^+$: 303.0462	MS^2[301.0349]: 283.0221(1.27), 273.0359(4.34), 271.0265(5.52), 243.0196(1.1), 229.0546(3.45), 227.0385(2.73), 193.0197(3.38), 178.9972(46.63), 151.0073(100.0), 121.0354(3.27), 107.0152(6.98)
**M71**	51.583	$C_{15}H_{10}O_{10}S$	380.9933	Quercetin-5-*O*-sulphate	$[M + H]^+$: 383.0062	MS^2[380.9944]: 301.0337(100.0), 179.0002(2.12), 151.0086(2.11); MS^3[301.0336]: 227.0287(7.24), 217.0536(9.5), 213.038(18.99), 199.0422(4.84), 183.042(7.29), 179.0017(32.68), 163.027(12.95), 151.0048(100.0), 107.0212(15.59)
**M72**	52.647	$C_{15}H_{10}O_{10}S$	380.9932	Quercetin-7-*O*-sulphate	$[M + H]^+$: 383.0058	MS^2[380.9932]: 301.0335(100.0); MS^3[301.0334]: 211.0305(100.0), 161.038(72.13)
**M73**	56.300	$C_{15}H_{10}O_{10}S$	380.9922	Quercetin- 4′-*O*-sulphate	$[M + H]^+$: 382.9957	MS^2[380.9962]: 301.0336(100.0), 179.0036(7.84), 151.0063(5.4); MS^3[301.0336]: 285.1062(7.11), 229.0392(10.67), 211.0474(14.22), 179.0042(26.53), 151.0064(100.0)

	t_R(min)	Formula	Meas. *m*/*z*	Identification	Other Ions	HRMS^n(Characteristic Fragment Ions and Their Relative Abundance)
**M74**	57.033	$C_{15}H_{10}O_{10}S$	380.9932	Quercetin-3′-*O*-sulphate	$[M + H]^+$: 383.0044	MS^2[380.9933]: 301.0332(100.0), 179.0019(10.35), 151.0081(5.43); MS^3[302.0374]: 273.0391(11.66), 229.0529(9.83), 193.0174(7.62), 179.0024(69.17), 151.0093(100.0), 121.0308(1.09), 107.0187(4.38)
**M75**	58.173	$C_{15}H_{10}O_{10}S$	380.9937	Quercetin-3-*O*-sulphate		MS^2[380.9871]: 301.0323(100.0), 300.0739(4.11), 179.0083(24.98), 151.0033(22.85)
**M76**	37.542	$C_{21}H_{18}O_{13}$	477.0688	Quercetin glucuronide	$[M + H]^+$: 479.0798	MS^2[477.0773]: 343.0457(5.11), 301.032(100.0), 273.0359(1.86), 178.997(17.1), 161.0301(2.79), 151.0004(1.86)
**M77**	40.727	$C_{21}H_{18}O_{16}S$	557.0252	Quercetin glucuronide sulphate 1	$[M + Na]^+$: 580.9350	MS^2[557.0273]: 477.0707(3.6), 301.0325(100.0), 273.0423(1.64) MS^3[301.0325]: 178.9978(62.02), 152.0029(50.0), 151.012(100.0)
**M78**	41.068	$C_{21}H_{18}O_{16}S$	557.0268	Quercetin glucuronide sulphate 2	$[M + 2H_2O - H]^-$: 593.0172	MS^2[557.0302]: 477.0682(5.04), 380.9827(0.67), 301.0326(100.0), 179.0052(2.62); MS^3[301.0324]: 229.0519(86.98), 151.0109(100.0)
**M79**	41.443	$C_{21}H_{18}O_{16}S$	557.0269	Quercetin glucuronide sulphate 3	$[M + HCOOH - H]^-$: 602.9751	MS^2[557.0277]: 380.9935(3.47), 301.0342(100.0), 178.9903(0.91)
**M80**	65.417	$C_{16}H_{12}O_7$	315.0503	Isorhamnetin	$[M + H]^+$: 317.0623	MS^2[315.0504]: 301.0307(30.16), 300.0274(100.0), 271.0252(5.39), 227.0417(3.37), 151.0064(7.64); MS^3[299.8252]: 271.0263(100.0), 255.0645(8.31), 243.0281(14.39), 228.0799(16.62), 227.0396(33.01), 151.0095(24.7), 107.0076(8.31)
**M81**	48.633	$C_{16}H_{12}O_{10}S$	395.0081	Isorhamnetin-5-*O*-sulphate	$[M + H]^+$: 397.0162	MS^2[395.0083]: 315.0478(100.0), 300.0257(9.79), 228.037(2.71), 151.0054(1.35), 107.9698(0.77); MS^3[315.0478]: 300.0212(100.0), 284.0198(6.56), 273.0359(4.43), 228.037(8.78), 192.0157(4.43)
**M82**	56.917	$C_{16}H_{12}O_{10}S$	395.0082	Isorhamnetin-7-*O*-sulphate	$[M + H]^+$: 397.0179	MS^2[395.0084]: 315.0491(100.0), 300.0279(10.27), 287.0443(0.94), 151.0098(4.64); MS^3[315.0492]: 300.0297(100.0), 285.0262(7.32), 284.0294(9.19), 272.0353(25.84), 151.0115(67.22), 149.4103(7.32)
**M83**	58.042	$C_{16}H_{12}O_{10}S$	395.0085	Isorhamnetin-3-*O*-sulphate	$[M + H]^+$: 397.0235	MS^2[395.0086]: 315.0487(100.0), 301.0291(1.79), 300.0266(17.54), 283.0312(0.13); MS^3[315.0487]: 301.0305(42.99), 300.0265(100.0), 271.0254(4.58), 227.0376(2.67), 151.011(8.71)
**M84**	58.922	$C_{16}H_{12}O_{10}S$	395.0082	Isorhamnetin-4′-*O*-sulphate	$[M + H]^+$: 396.9741 $[M + HCOOH - H]^-$: 441.0783	MS^2[395.0054]: 315.0481(100.0), 300.0299(33.72), 297.0348(5.5), 289.0695(3.69), 221.0411(7.31), 165.0446(3.69), 152.0379(3.69), 137.0147(3.69); MS^3[315.0481]: 300.0352(100.0), 299.9024(11.01)
**M85**	48.308	$C_{16}H_{12}O_{13}S_2$	474.9658	Isorhamnetin disulphate	$[M + HCOOH - H]^-$: 520.9840	MS^2[474.9694]: 395.0033(83.92), 315.0486(100.0); MS^3[315.0486]: 300.0299(100.0)
**M86**	49.212	$C_{22}H_{20}O_{13}$	491.0852	Isorhamnetin-4′-*O*-glucuronide	$[M + H]^+$: 493.0949	MS^2[491.0855]: 315.0516(100.0), 300.0276(34.63), 231.0357(1.74); MS^3[315.0516]: 300.0311(100.0), 151.012(7.71)
**M87**	50.428	$C_{22}H_{20}O_{13}$	491.0836	Isorhamnetin-7-*O*-glucuronide	$[M + H]^+$: 493.1021	MS^2[491.0838]: 315.0505(100.0), 301.0252(7.02), 300.0301(31.77); MS^3[315.0504]: 300.0401(100.0)
**M88**	40.143	$C_{22}H_{20}O_{16}S$	571.0381	Isorhamnetin glucuronide sulphate 1	$[M + H]^+$: 573.0258	MS^2[571.038]: 491.0783(1.6), 315.0491(100.0), 300.0234(26.2), 271.021(1.9); MS^3[315.0492]: 300.0288(100.0)
**M89**	41.118	$C_{22}H_{20}O_{16}S$	571.0413	Isorhamnetin glucuronide sulphate 2	$[M + H]^+$: 573.0551	MS^2[571.0411]: 491.0842(5.07), 411.0694(0.3), 315.0489(100.0), 300.0282(31.47), 271.0216(1.94); MS^3[315.049]: 300.0252(100.0),

	t_R(min)	Formula	Meas. *m/z*	Identification	Other Ions	HRMS^n(Characteristic Fragment Ions and Their Relative Abundance)
						272.0328(4.93), 271.021(1.66), 255.0191(2.21), 243.0344(1.66), 151.0353(3.29)
**M90**	44.673	$C_{22}H_{20}O_{16}S$	571.0395	Isorhamnetin glucuronide sulphate 3		MS^2[571.0499]: 491.0803(14.58), 316.0519(18.16), 315.0491(100.0)
**M91**	45.392	$C_{22}H_{20}O_{16}S$	571.0419	Isorhamnetin glucuronide sulphate 4		MS^2[571.0425]: 491.0923(4.72), 395.0117(1.58), 315.0478(100.0), 300.024(14.53); MS^3[315.0476]: 301.0262(24.92), 300.0268(100.0), 283.0162(16.83), 243.0265(20.87), 151.0012(41.75)
**M92**	27.987	$C_{15}H_{10}O_{11}S$	396.9882	Hydroxylated quercetin sulphate 1	[M + H]$^+$: 399.0226	MS^2[396.9883]: 317.0295(41.74), 299.0193(28.16), 273.0437(17.99), 258.0207(6.24), 255.0264(10.43), 227.0326(7.49), 211.0391(4.16), 206.9943(24.59), 190.9997(100.0), 183.042(3.31), 175.0027(1.66), 163.0048(32.05)
**M93**	28.487	$C_{15}H_{10}O_{11}S$	396.9868	Hydroxylated quercetin sulphate 2	[M + H]$^+$: 398.9855	MS^2[396.9902]: 317.0298(54.7), 299.0187(24.85), 273.0326(14.75), 258.0128(4.86), 255.0282(17.0), 227.0323(3.65), 207.0018(26.94), 190.9991(100.0), 179.0029(4.86), 163.0064(20.04), 135.0083(3.65)
**M94**	29.028	$C_{15}H_{10}O_{11}S$	396.9876	Hydroxylated quercetin sulphate 3	[M + H]$^+$: 399.0144	MS^2[396.9902]: 317.0281(74.16), 300.0729(4.26), 299.0286(7.41), 273.0359(10.56), 258.0136(7.41), 255.0307(25.43), 231.0285(8.44), 228.0442(6.39), 211.0391(5.32), 208.0002(7.41), 206.994(32.76), 191.0013(100.0), 163.0041(40.13)
**M95**	15.930	$C_{21}H_{18}O_{14}$	493.0642	Hydroxylated quercetin glucuronide 1	[M + HCOOH − H]$^-$: 539.1279	MS^2[493.0656]: 343.0829(2.42), 317.0289(100.0), 299.0217(26.78), 255.0357(32.73), 207.0024(38.18), 191.0001(88.23); MS^3[317.0288]: 296.0672(66.67), 227.0228(100.0), 190.9935(66.67)
**M96**	17.720	$C_{21}H_{18}O_{14}$	493.0601	Hydroxylated quercetin glucuronide 2	[M + H]$^+$: 495.0604	MS^2[493.0664]: 317.0277(90.58), 299.0181(21.56), 255.0314(18.18), 206.9961(33.94), 190.9985(100.0), 163.0115(9.09)
**M97**	39.160	$C_{16}H_{12}O_{11}S$	411.0022	Hydroxylated isorhamnetin sulphate 1		MS^2[411.0058]: 332.0518(11.57), 331.0425(78.23), 303.0488(100.0), 287.0523(3.17), 275.0529(28.07), 207.001(14.06), 152.0192(2.64); MS^3[303.049]: 276.0959(15.89), 275.0593(100.0), 260.0211(35.83)
**M98**	39.710	$C_{16}H_{12}O_{11}S$	411.0043	Hydroxylated isorhamnetin sulphate 2	[M + H]$^+$: 413.0005	MS^2[411.0043]: 331.0433(73.82), 304.0531(8.54), 303.0509(100.0), 287.0604(2.17), 275.0551(35.38), 244.0394(2.17), 151.012(2.17); MS^3[303.051]: 275.0516(100.0), 259.0317(23.63), 256.0334(13.69)
**M99**	40.193	$C_{16}H_{12}O_{11}S$	411.0039	Hydroxylated isorhamnetin sulphate 3		MS^2[411.0083]: 332.0503(18.28), 331.0462(79.96), 313.022(2.34), 303.0499(100.0), 287.053(7.45), 275.0554(44.52), 151.012(2.34); MS^3[303.0499]: 275.0553(100.0), 260.0189(34.75), 259.0273(95.74)
**M100**	59.017	$C_{16}H_{12}O_{11}S$	411.0030	Hydroxylated isorhamnetin sulphate 4		MS^2[411.0031]: 331.0433(86.27), 313.0341(50.37), 303.0471(100.0), 287.0443(3.73), 233.0641(3.1), 195.0459(2.47), 179.0029(2.47), 175.0403(3.73), 153.0321(2.47); MS^3[303.0471]: 285.0262(50.48), 175.034(50.48), 153.0204(100.0)
**M101**	25.103	$C_{22}H_{20}O_{14}$	507.0790	Hydroxylated isorhamnetin glucuronide 1	[M + H]$^+$: 509.0944	MS^2[507.0858]: 331.0414(60.2), 303.0472(100.0), 275.0568(33.72), 179.0079(27.21); MS^3[303.0471]: 275.0583(100.0), 260.0342(66.23), 259.0181(83.12)
**M102**	25.728	$C_{22}H_{20}O_{14}$	507.0758	Hydroxylated isorhamnetin glucuronide 2	[M + H]$^+$: 509.0874	N

	t_R(min)	Formula	Meas. *m/z*	Identification	Other Ions	HRMS^n(Characteristic Fragment Ions and Their Relative Abundance)
**M103**	26.570	$C_{22}H_{20}O_{14}$	507.0805	Hydroxylated isorhamnetin glucuronide 3	$[M + H]^+$: 509.0877	MS^2[507.0804]: 475.0432(4.94), 331.0466(98.68), 303.0516(100.0), 275.0573(59.71), 259.0212(6.2), 217.0884(4.94), 179.0013(13.66); MS^3[303.0516]: 275.0531(100.0)
**M104**	40.733	$C_{15}H_{12}O_6$	287.0557	Eriodictyol	$[M + H]^+$: 289.0645	MS^2[287.0557]: 269.0482(5.27), 241.0493(7.48), 175.034(1.76), 165.0205(100.0), 137.0222(5.97), 135.0046(0.75), 107.0174(0.51)
**M105**	49.442	$C_{15}H_{12}O_6$	287.0555	Dihydrokaempferol	$[M + H]^+$: 289.0721	MS^2[287.0555]: 269.0368(4.63), 259.0621(100.0), 243.0647(4.57), 201.0554(8.75), 173.0683(12.96), 125.0317(17.71); MS^3[260.0672]: 215.0703(39.08), 173.0659(100.0), 151.0111(15.31), 137.0258(4.83), 125.029(60.18), 107.0076(3.2)
**M106**	37.325	$C_{15}H_{12}O_9S$	367.0128	Eriodictyol-7-*O*-sulphate	$[M + H]^+$: 369.0350	MS^2[367.0125]: 287.0543(41.18), 269.0465(4.37), 165.0219(100.0), 137.0308(4.67)
**M107**	37.708	$C_{15}H_{12}O_9S$	367.0144	Dihydrokaempferol-7-*O*-sulphate	$[M + H]^+$: 369.0123 $[M+HCOOH-H]^-$: 413.0559	MS^2[367.0143]: 287.055(100.0), 269.0496(7.44), 259.0621(95.06), 243.0699(1.87), 165.0203(2.27); MS^3[287.0548]: 259.0692(100.0), 241.0454(5.36), 215.063(5.36), 125.0216(7.99)
**M108**	38.200	$C_{15}H_{12}O_9S$	367.0144	Eriodictyol-3′-*O*-sulphate	$[M + H]^+$: 369.0131 $[M + CH_3HCOOH - H]^-$: 427.0378	MS^2[367.0143]: 287.0584(100.0), 269.0524(3.42), 165.0203(52.17); MS^3[287.0584]: 244.913(45.3), 165.027(100.0)
**M109**	40.383	$C_{15}H_{12}O_9S$	367.0123	Dihydrokaempferol- 4′-*O*-sulphate		MS^2[367.0121]: 297.0793(5.6), 287.0555(100.0), 269.0922(5.6), 259.0651(95.85), 243.0575(5.6); MS^3[287.0555]: 259.0719(100.0), 243.0649(44.81)
**M110**	28.045	$C_{21}H_{20}O_{12}$	463.0907	Dehydroxylated taxifolin glucuronide 1	$[M + H]^+$: 465.0951	N
**M111**	28.753	$C_{21}H_{20}O_{12}$	463.0856	Dehydroxylated taxifolin glucuronide 2	$[M + H]^+$: 465.1055	N
**M112**	28.970	$C_{21}H_{20}O_{12}$	463.0888	Dihydrokaempferol 4′-*O*-glucuronide	$[M + H]^+$: 465.0837	MS^2[463.0889]: 329.0731(10.11), 301.0674(17.49), 287.0565(77.45), 259.0566(100.0), 221.0609(12.54)
**M113**	16.017	$C_{21}H_{18}O_{12}$	478.1007	Luteolin-7-*O*- glucuronide	$[M + NH_4]^+$: 480.1114	MS^2[478.1044]: 461.0707(17.38), 285.0386(100.0), 223.0349(1.78), 217.0536(2.54), 175.0423(1.52); MS^3[285.0386]: 175.034(100.0)
**M114**	16.525	$C_{21}H_{18}O_{12}$	478.1007	Luteolin-3′/4′-*O*-glucuronide	$[M + NH_4]^+$: 480.1122	MS^2[478.1031]: 461.0765(14.77), 285.0408(100.0), 241.0527(1.75), 217.0548(2.98), 177.0255(5.34), 175.044(2.46), 163.0078(4.04); MS^3[285.0408]: 199.0339(76.0), 177.0318(100.0)
**M115**	17.425	$C_{21}H_{18}O_{12}$	478.1014	Luteolin-3′/4′-*O*-glucuronide	$[M + NH_4]^+$: 480.1120	MS^2[478.1014]: 461.0754(20.56), 285.0374(100.0), 241.0573(4.06), 217.0502(5.89), 177.0169(3.99), 175.044(1.34); MS^3[285.0374]: 243.0492(49.84), 239.0645(49.84), 217.0575(100.0), 175.034(49.84), 163.0311(33.87)
**M116**	23.962	$C_{22}H_{20}O_{12}$	492.1165	Methyl luteolin glucuronide	$[M + NH_4]^+$: 494.1282	MS^2[492.1144]: 475.0829(30.71), 299.0511(100.0), 284.0283(9.33);MS^3[299.0498]: 284.0412(100.0), 256.027(35.16), 188.0416(35.16)

	t_R(min)	Formula	Meas. *m*/*z*	Identification	Other Ions	HRMS^n(Characteristic Fragment Ions and Their Relative Abundance)
**M117**	43.883	$C_{15}H_{14}O_7$	305.0652	Hydrogenated taxifolin	[M + H]$^+$: 307.0816	MS^2[305.2124]: 287.0565(27.3), 243.0639(5.84), 183.0309(84.98), 165.0249(21.98), 161.0287(19.64), 137.0301(10.2), 125.0274(100.0); MS^3[287.0565]: 174.0706(14.4), 161.0202(57.2), 125.0275(100.0)
**M118**	52.325	$C_{16}H_{16}O_7$	319.0813	Hydrogenated methyl taxifolin	[M + H]$^+$: 321.0935	MS^2[319.0794]: 317.073(2.11), 301.0722(7.8), 289.0676(13.17), 239.0889(9.24), 221.0862(10.03), 165.0267(30.46), 164.0131(100.0), 125.0282(23.45)
**M119**	38.567	$C_{15}H_{14}O_{10}S$	385.0224	Hydrogenated taxifolin sulphate 1		MS^2[385.0225]: 305.0606(100.0), 303.0496(2.04), 287.058(14.45), 183.0371(49.64), 161.029(8.54), 125.0286(8.04); MS^3[305.0605]: 183.0274(100.0)
**M120**	43.433	$C_{15}H_{14}O_{10}S$	385.0224	Hydrogenated taxifolin sulphate 2	[M + H]$^+$: 387.0402	MS^2[385.0226]: 305.0639(100.0), 287.0531(34.27), 183.0313(74.2), 179.037(5.95), 165.0221(16.19), 161.0286(18.96), 125.0318(28.64); MS^3[305.0638]: 219.0603(13.22), 183.028(81.64), 137.0244(14.5), 125.0307(100.0), 123.0563(5.33)
**M121**	45.442	$C_{15}H_{14}O_{10}S$	385.0227	Hydrogenated taxifolin sulphate 3	[M + H]$^+$: 387.0354	MS^2[385.0228]: 305.0635(100.0), 303.0609(3.84), 287.0568(36.85), 221.0473(19.37), 183.0345(56.85), 165.0244(19.71), 161.0269(14.49), 125.0305(20.95); MS^3[305.0634]: 272.8169(13.6), 221.0411(13.6), 183.0361(81.21), 177.0444(13.6), 160.964(13.6), 134.7956(13.6), 125.0263(100.0)
**M122**	35.317	$C_9H_{10}O_3$	165.0555	3/4-Hydroxyphenylpropionic acid	[M + HCOOH − H]$^-$: 211.0672	N
**M123**	35.917	$C_9H_{10}O_3$	165.0570	3/4-Hydroxyphenylpropionic acid	[M + H]$^+$: 167.0834 [M + HCOOH − H]$^-$: 211.0611	MS^2[165.0566]: 121.0738(100.0), 119.0403(5.32), 106.0355(5.32)
**M124**	21.712	$C_9H_{10}O_6S$	245.0132	4- Hydroxyphenylpropionic acid sulphate	[M + H]$^+$: 247.0623 [M + CH_3COOH − H]$^-$: 305.0807	MS^2[245.0085]: 165.058(100.0), 121.0659(51.43), 118.9012(2.54)
**M125**	23.683	$C_9H_{10}O_6S$	245.0133	3- Hydroxyphenylpropionic acid sulphate	[M + H]$^+$: 247.0701	MS^2[245.0133]: 165.058(70.75), 121.0704(100.0), 119.054(20.97); MS^3[165.0581]: 121.0892(100.0)
**M126**	23.787	$C_{15}H_{18}O_9$	341.0866	3/4-Hydroxyphenylpropionic acid glucuronide	[M + H]$^+$: 343.0897	N
**M127**	24.078	$C_{15}H_{18}O_9$	341.0891	3/4-Hydroxyphenylpropionic acid glucuronide	[M+H]$^+$: 343.0984	MS^2[341.0891]: 323.0638(39.84), 319.516(60.16), 285.0357(39.84), 175.0225(60.16), 165.0477(60.16), 121.0641(100.0)
**M128**	22.325	$C_9H_8O_6S$	242.9969	*p*/*m*-Coumaric acid sulphate	[M + H]$^+$: 245.1021	MS^2[242.9968]: 199.0183(2.83), 163.0435(100.0), 119.0546(42.98)
**M129**	25.758	$C_9H_8O_6S$	242.9972	*p*/*m*-Coumaric acid sulphate	[M + H]$^+$: 245.0905	MS^2[242.9999]: 163.0438(100.0), 119.0602(17.51)
**M130**	27.067	$C_9H_8O_6S$	242.9971	*p*/*m*-Coumaric acid sulphate		MS^2[242.9971]: 163.0429(100.0), 119.0548(18.77); MS^3[163.0429]: 119.0495(100.0)
**M131**	16.490	$C_8H_8O_4$	167.0349	Dihydroxyphenylacetic acid	[M + H]$^+$: 169.0487	MS^2[167.0352]: 123.0458(100.0)

	t_R(min)	Formula	Meas. *m*/*z*	Identification	Other Ions	HRMS^n(Characteristic Fragment Ions and Their Relative Abundance)
**M132**	16.258	$C_8H_8O_7S$	246.9927	Dihydroxyphenylacetic acid sulfae 1	[M + HCOOH − H]⁻: 293.0782	MS²[246.9926]: 203.0035(98.33), 167.0337(3.62), 123.0502(100.0), 96.9592(2.41), 80.9685(2.41)
**M133**	15.800	$C_8H_8O_7S$	246.9927	Dihydroxyphenylacetic acid sulfae 2	[M + H]⁺: 249.0907 [M + HCOOH − H]⁻: 293.0674	MS²[246.9927]: 203.0043(40.61), 167.1071(4.54), 123.047(100.0), 96.9592(7.65)
**M134**	16.933	$C_8H_8O_7S$	246.9920	Dihydroxyphenylacetic acid sulfae 3	[M + HCOOH − H]⁻: 293.0765	MS²[246.9921]: 203.0052(25.11), 123.0481(100.0), 104.9069(4.22), 96.9592(6.37)
**M135**	18.108	$C_9H_{10}O_7S$	261.0073	Homovanillic acid sulphate		MS²[261.0072]: 217.0189(60.08), 181.0569(100.0), 137.0659(69.26), 122.0471(14.26), 107.0468(0.79); MS³[181.057]: 137.0775(100.0), 123.052(28.58), 122.0393(23.29)
**M136**	22.508	$C_9H_{10}O_4$	181.0504	Dihydrocaffeic acid	[M + H]⁺: 183.0802 [M + HCOOH − H]⁻: 227.0506	MS²[181.0504]: 137.0642(100.0)
**M137**	20.033	$C_9H_{10}O_7S$	261.0082	Dihydrocaffeic acid sulphate 1	[M + H]⁺: 263.0382	MS²[261.0082]: 217.0208(8.69), 181.0563(77.44), 137.0644(100.0), 119.1207(2.90)
**M138**	20.942	$C_9H_{10}O_7S$	261.0084	Dihydrocaffeic acid sulphate 2		MS²[261.0085]: 181.0559(100.0), 137.0673(55.09), 119.0552(5.14); MS³[181.0559]: 137.059(100.0), 107.9501(66.67)
**M139**	13.108	$C_{11}H_{13}NO_5$	238.0720	Caffeic acid acetamide 1	[M + H]⁺: 240.0842	MS²[238.072]: 179.0431(100.0), 176.0566(22.08), 152.0729(22.08), 135.042(55.63), 123.0528(33.33)
**M140**	13.592	$C_{11}H_{13}NO_5$	238.0724	Caffeic acid acetamide 2	[M + H]⁺: 240.0925	MS²[238.0762]: 198.0597(49.02), 196.0722(49.02), 179.06(49.02), 176.0705(61.76), 135.0555(100.0)
**M141**	13.858	$C_{11}H_{13}NO_5$	238.0728	Caffeic acid acetamide 3	[M + H]⁺: 240.0822	MS²[238.0729]: 220.0566(23.69), 196.0632(94.08), 179.0505(100.0), 161.0541(35.54), 135.0502(53.3), 122.1231(23.69), 107.8813(23.69)
**M142**	11.692	$C_9H_{10}O_5$	197.0461	3-(3,4-Dihydroxyphenyl)-3-hydroxypropanoic acid	[M + H]⁺: 199.0994	MS²[197.0486]: 179.0378(65.39), 135.0627(100.0)
**M143**	12.658	$C_9H_{10}O_5$	197.0456	3-(3,4-Dihydroxyphenyl)-2-hydroxypropanoic acid	[M + H]⁺: 199.0860	MS²[197.0482]: 179.0353(100.0), 135.0474(18.06)
**M144**	12.700	$C_9H_{10}O_8S$	277.0024	Caffeic acid hydrated sulphate 1	[M + HCOOH − H]⁻: 323.0367	MS²[277.0025]: 258.9906(3.42), 230.9964(29.18), 214.9979(28.31), 197.0456(100.0), 179.0362(75.84), 151.0353(2.75), 149.0285(2.75), 135.0497(46.29), 123.048(14.48), 107.0517(2.75)
**M145**	13.433	$C_9H_{10}O_8S$	277.0025	Caffeic acid hydrated sulphate 2	[M + HCOOH − H]⁻: 323.0610	MS²[277.0027]: 258.9884(6.27), 215.0035(41.39), 197.0484(86.06), 179.0394(100.0), 151.0403(4.02), 135.0514(78.31), 123.0497(4.57), 107.0664(2.29); MS³[179.0394]: 135.0486(100.0)
**M146**	22.667	$C_{10}H_{12}O_7S$	275.0236	Dihydrogen ferulic acid sulphate		MS²[275.0236]: 195.0681(100.0), 151.0845(14.67), 149.0632(1.61), 136.0607(25.99), 119.0578(12.43)
**M147**	15.810	$C_{10}H_{12}O_8S$	291.0191	Ferulic acid hydratedsulphate 1	[M + H]⁺: 293.0529	MS²[291.0162]: 245.0016(5.26), 229.0245(5.26), 211.0622(100.0), 193.0556(92.66), 165.0517(4.21), 150.0371(4.21), 149.0654(5.26), 134.0406(35.58), 107.0624(4.21)
**M148**	16.233	$C_{10}H_{12}O_8S$	291.0184	Ferulic acid hydratedsulphate 2		MS²[291.0231]: 229.0105(18.43), 211.0577(36.68), 193.0594(100.0), 165.0568(27.64), 134.0369(68.74)

	t_R(min)	Formula	Meas. *m*/*z*	Identification	Other Ions	HRMS^n(Characteristic Fragment Ions and Their Relative Abundance)
**M149**	25.208	$C_7H_8O_5S$	203.0021	Hydroxybenzyl alcohol sulphate		MS^2[203.0021]: 123.0532(100.0), 80.9599(1.6), 79.9639(3.98)
**M150**	29.025	$C_{13}H_{16}O_8$	299.0773	Hydroxybenzyl alcohol glucuronide 1	[M + H]$^+$: 301.0777	MS^2[299.0773]: 283.0312(21.37), 175.0225(100.0), 160.2379(21.37), 127.583(31.85), 123.0572(89.52), 113.0266(84.27)
**M151**	29.717	$C_{13}H_{16}O_8$	299.0771	Hydroxybenzyl alcohol glucuronide 2	[M + H]$^+$: 301.0752	MS^2[299.0772]: 175.0269(100.0), 123.05(53.08), 113.0296(87.01)
**M152**	18.795	$C_{13}H_{16}O_{11}S$	379.0336	Hydroxybenzyl alcohol glucuronide sulphate 1	[M + Na]$^+$: 403.0538	MS^2[379.0367]: 299.073(13.22), 280.5353(13.22), 204.0215(26.43), 203.003(100.0), 182.9267(13.22), 175.0215(13.22)
**M153**	21.095	$C_{13}H_{16}O_{11}S$	379.0337	Hydroxybenzyl alcohol glucuronide sulphate 2	[M + Na]$^+$: 403.0728	MS^2[379.0337]: 325.2361(3.3), 299.073(4.86), 203.0028(100.0), 175.034(3.3), 159.0413(3.3), 123.0458(8.17)
**M154**	33.083	$C_8H_{10}O_5S$	217.0168	Methyl hydroxybenzyl alcohol sulphate 1	[M + HCOOH − H]$^-$: 263.0667	MS^2[217.0198]: 201.9975(9.57), 137.0699(88.45), 122.0442(100.0), 79.9576(9.57)
**M155**	34.625	$C_8H_{10}O_5S$	217.0181	Methyl hydroxybenzyl alcohol sulphate 2	[M + HCOOH − H]$^-$: 263.0530	MS^2[217.0181]: 202.0013(8.38), 137.0652(98.69), 122.0346(100.0)
**M156**	17.512	$C_7H_6O_6S$	216.9822	3/4-Hydroxy benzoic acid sulphate		MS^2[216.9822]: 137.0291(100.0)
**M157**	17.937	$C_7H_6O_6S$	216.9810	3/4-Hydroxy benzoic acid sulphate	[M + 2H_2O − H]$^-$: 252.9380	MS^2[216.9765]: 137.0236(100.0)
**M158**	30.987	$C_8H_8O_7S$	246.9914	Vanillic acid sulphate		MS^2[246.991]: 167.0362(100.0), 152.0208(2.6), 137.0317(2.6), 110.9775(2.6), 108.0293(2.6); MS^3[168.0317]: 152.0077(100.0)
**M159**	31.978	$C_8H_8O_7S$	246.9909	Isovanillic acid sulphate		MS^2[246.9898]: 167.0427(100.0), 152.0253(11.02), 110.978(3.24)
**M160**	61.342	$C_{31}H_{24}O_{13}$	603.1151	Dimer of taxiflolin and dehydroxylated methyl taxifolin	[M + H]$^+$: 605.1226	MS^2[603.1152]: 429.0094(2.05), 315.0449(6.63), 303.0557(4.08), 299.0515(36.21), 297.0388(100.0), 285.0433(33.47), 269.0479(29.94), 253.0522(25.92), 241.0552(13.28), 227.0355(28.46); MS^3[297.0388]: 227.0299(100.0)
**M161**	55.533	$C_{31}H_{24}O_{14}$	619.1063	Dimer of taxiflolin and methyl taxifolin 1	[M + H]$^+$: 621.1186	MS^2[619.106]: 315.046(15.33), 303.0519(7.41), 297.0383(100.0), 285.0391(34.96), 241.0485(6.04), 227.036(29.0); MS^3[297.0383]: 279.0147(26.67), 269.0446(40.0), 255.0191(26.67), 253.0569(26.67), 235.0548(26.67), 227.0384(100.0)
**M162**	60.600	$C_{31}H_{24}O_{14}$	619.1090	Dimer of taxiflolin and methyl taxifolin 2	[M + H]$^+$: 621.1206	MS^2[619.1091]: 315.0475(16.72), 303.0475(4.28), 297.0383(100.0), 285.0371(30.42), 269.0421(8.15), 241.0586(9.68), 227.0328(25.51); MS^3[297.0383]: 269.0368(11.99), 253.0486(21.7), 241.0552(11.99), 227.031(100.0), 225.0485(11.99)
**M163**	64.608	$C_{32}H_{26}O_{14}$	633.1249	Dimer of taxiflolin and dimethyl taxifolin	[M + H]$^+$: 635.1386	MS^2[633.1249]: 330.0702(14.17), 329.0637(15.62), 303.0496(8.48), 297.0395(100.0), 285.0388(39.82), 269.0446(11.27), 241.0517(9.87), 227.0345(35.53), 197.0614(11.27)
**M164**	56.025	$C_{31}H_{24}O_{17}S$	699.0699	Dimer of taxiflolin and methyl taxifolin sulphate 1	[M + H]$^+$: 701.0665	MS^2[699.0759]: 619.1047(19.07), 601.093(1.74), 383.0133(5.56), 315.0472(34.65), 303.044(3.82), 297.0376(100.0), 285.0378(28.94),

	t_R(min)	Formula	Meas. *m*/*z*	Identification	Other Ions	HRMS^n(Characteristic Fragment Ions and Their Relative Abundance)
						241.0454(8.53), 227.0367(23.27); MS^3[297.0375]: 269.0446(57.14), 255.0251(71.43), 227.0289(100.0)
**M165**	56.750	$C_{31}H_{24}O_{17}S$	699.0671	Dimer of taxiflolin and methyl taxifolin sulphate 2		MS^2[699.0674]: 619.1041(55.56), 601.093(1.27), 315.0477(44.86), 303.0496(2.13), 297.0371(100.0), 285.0377(17.5), 241.0503(2.57), 227.0361(10.34); MS^3[297.037]: 227.0386(100.0)
**M166**	60.817	$C_{31}H_{24}O_{17}S$	699.0678	Dimer of taxiflolin and methyl taxifolin sulphate 3	[M + H]$^+$: 701.0781	MS^2[699.0706]: 619.106(15.71), 383.0068(5.18), 315.0466(31.98), 303.0475(11.98), 297.0376(100.0), 285.0367(31.91), 241.0495(6.36), 227.0356(19.51); MS^3[297.0375]: 269.0444(19.89), 255.0327(20.25), 227.034(100.0), 225.0544(17.46), 197.0547(6.34), 173.0235(5.92)
**M167**	59.725	$C_{32}H_{26}O_{17}S$	713.0844	Dimer of taxiflolin and dimethyl taxifolin sulphate 1	[M + H]$^+$: 715.0883	MS^2[713.0846]: 633.121(78.77), 395.0105(1.13), 383.0081(1.33), 330.0682(9.95), 329.064(55.43), 317.0642(29.04), 315.0486(34.88), 303.0436(9.74), 301.0732(7.27), 299.0485(0.76), 298.0384(15.99), 297.0387(100.0),289.0666(6.5),285.0448(30.95),269.0458(4.0), 253.0477(4.4), 241.0479(7.26), 227.0358(17.51)
**M168**	60.167	$C_{32}H_{26}O_{17}S$	713.0839	Dimer of taxiflolin and dimethyl taxifolin sulphate 2	[M + H]$^+$: 715.0630	MS^2[713.084]: 633.1203(100.0), 329.0637(10.29), 315.0486(30.08), 303.0451(6.63), 297.0403(69.79), 289.0534(0.63), 285.0382(8.55), 227.0323(7.8); MS^3[633.1202]: 329.0628(15.18), 315.0526(9.1), 303.0331(6.03), 301.058(6.03), 297.0399(100.0), 285.0469(46.0), 269.0601(6.03), 241.0559(10.57), 227.0344(24.22), 199.1007(6.03)
**M169**	64.125	$C_{32}H_{26}O_{17}S$	713.0843	Dimer of taxiflolin and dimethyl taxifolin sulphate 3	[M + H]$^+$: 715.0977	MS^2[713.0842]: 633.1208(54.36), 330.0683(5.99), 329.0632(22.97), 317.0634(9.76), 315.047(29.92), 303.0488(10.78), 298.0414(21.45), 297.0382(100.0), 285.0372(21.79), 269.0441(8.23), 227.0363(9.55); MS^3[297.0382]: 269.0498(41.76), 255.0307(19.55), 253.0517(28.29), 241.0527(5.45), 227.0335(100.0), 225.0544(26.44), 211.0461(7.71)
**M170**	60.650	$C_{32}H_{26}O_{13}$	617.1291	Dimer of methyl taxiflolin and dehydroxylated methyl taxifolin 1	[M + H]$^+$: 619.1473	MS^2[617.1266]: 329.0627(100.0), 313.0705(58.45), 299.0496(27.28), 297.0278(94.74), 227.0285(19.48), 217.0047(15.59)
**M171**	64.400	$C_{32}H_{26}O_{13}$	617.1311	Dimer of methyl taxiflolin and dehydroxylated methyl taxifolin 2	[M + H]$^+$: 619.1407	MS^2[617.1313]: 330.0665(13.11), 329.0643(100.0), 317.0627(34.37), 301.0707(16.82), 300.0566(15.75), 299.052(71.1), 289.0705(27.38), 285.0742(15.72), 281.0495(21.43); MS^3[329.0643]: 270.0552(31.07), 136.0354(100.0)
**M172**	64.925	$C_{32}H_{26}O_{13}$	617.1299	Dimer of methyl taxiflolin and dehydroxylated methyl taxifolin 3	[M + H]$^+$: 619.1315	MS^2[617.1281]: 313.0705(54.0), 304.056(4.69), 301.0727(15.12), 297.0385(100.0), 285.0434(44.87), 269.0509(5.82), 241.0527(6.95), 227.0386(34.84), 211.0336(6.95), 189.0484(6.95); MS^3[297.0385]: 227.0656(100.0), 227.0299(66.67)
**M173**	65.142	$C_{32}H_{24}O_{14}$	631.1093	Dimer of methyl quercetin and methyl taxifolin 1	[M + H]$^+$: 633.1569	MS^2[631.1112]: 615.115(8.3), 509.0973(100.0), 495.0759(7.37), 343.0303(5.51), 329.0608(47.85), 317.0621(15.64), 315.0491(25.73), 314.0673(5.55), 313.0713(24.84), 303.0496(3.72), 301.058(3.72), 299.073(7.44), 297.0398(34.1), 289.0695(7.37), 285.0367(26.48), 241.0454(3.72)

	t_R(min)	Formula	Meas. *m*/*z*	Identification	Other Ions	HRMS^n(Characteristic Fragment Ions and Their Relative Abundance)
**M174**	66.142	$C_{32}H_{24}O_{14}$	631.1088	Dimer of methyl quercetin and methyl taxifolin 2	$[M + H]^+$: 633.1517	MS²[631.1091]: 329.0633(17.71), 317.0641(7.07), 313.0317(100.0), 301.048(23.97), 289.0635(2.99), 285.0811(2.63), 241.0567(7.08), 213.0424(2.25), 207.0355(2.25); MS³[313.0317]: 269.0368(85.08), 241.0454(57.46), 198.0331(85.08), 175.0027(85.08), 163.0035(100.0)
**M175**	68.517	$C_{32}H_{24}O_{14}$	631.1106	Dimer of methyl quercetin and methyl taxifolin 3	$[M + H]^+$: 633.1196	MS²[631.1107]: 479.0975(5.04), 355.0434(7.47), 329.068(100.0), 317.0679(22.55), 313.0345(21.29), 301.0495(70.28), 289.0695(25.02), 285.0502(5.04), 257.0648(7.47), 241.0454(9.99)
**M176**	69.230	$C_{32}H_{24}O_{14}$	631.1105	Dimer of methyl quercetin and methyl taxifolin 4	$[M + H]^+$: 633.1162	MS²[631.1044]: 453.0727(6.78), 369.0622(6.78), 327.0433(13.64), 315.0606(36.03), 300.0175(9.05), 297.0385(100.0), 285.0415(37.54), 227.0317(18.23)
**M177**	64.550	$C_{33}H_{28}O_{13}$	631.1435	Dimer of methyl taxiflolin and dehydroxylated dimethyl taxifolin 1	$[M + H]^+$: 633.1605	MS²[631.1416]: 329.0634(88.41), 324.0928(33.48), 317.0646(44.21), 313.0722(100.0), 301.0662(33.48)
**M178**	67.408	$C_{33}H_{28}O_{13}$	631.1482	Dimer of methyl taxiflolin and dehydroxylated dimethyl taxifolin 2	$[M + H]^+$: 633.1335	MS²[631.1483]: 380.9735(5.27), 329.0625(88.35), 317.0722(42.47), 313.0697(100.0), 301.0662(10.34), 299.0485(5.27), 289.0715(30.98), 273.0776(7.81), 192.0157(10.34)
**M179**	67.633	$C_{33}H_{28}O_{13}$	631.1488	Dimer of methyl taxiflolin and dehydroxylated dimethyl taxifolin 3	$[M + H]^+$: 633.1291	MS²[631.1421]: 329.0684(44.66), 317.0718(29.31), 313.0703(100.0), 301.0683(16.73), 298.0421(4.18), 289.0701(31.4), 285.0802(5.57), 280.0438(4.18), 273.0896(4.89), 253.0418(4.18)
**M180**	59.133	$C_{32}H_{26}O_{14}$	633.1257	Dimer of methyl taxiflolin and methyl taxifolin 1	$[M + H]^+$: 635.1213	MS²[633.1254]: 330.0724(4.49), 329.0641(72.09), 317.0639(33.46), 303.0496(2.45), 301.0646(6.07), 297.0364(100.0), 289.0718(13.36), 285.0496(46.07), 269.0391(9.7), 253.0478(4.05), 243.0281(2.84), 241.0556(7.3), 227.0352(18.6);MS³[297.0364]: 227.0299(100.0), 225.065(66.23)
**M181**	63.783	$C_{32}H_{26}O_{14}$	633.1252	Dimer of methyl taxiflolin and methyl taxifolin 2	$[M + H]^+$: 635.1386	MS²[633.1251]: 329.0625(12.42), 317.0713(10.84), 315.0453(9.56), 303.0487(4.23), 297.0377(100.0), 289.0673(8.81), 269.046(12.72), 253.0492(9.24), 241.0523(13.0), 227.0362(38.16), 225.0619(10.66); MS³[297.0376]: 253.0468(11.92), 227.037(100.0), 225.0627(11.92)
**M182**	69.755	$C_{33}H_{26}O_{14}$	645.1243	Dimer of methyl taxiflolin and dimethyl quercetin 1	$[M + H]^+$: 647.1483	MS²[645.1245]: 330.0647(19.51), 329.0635(99.94), 327.0475(100.0), 317.0643(58.75), 311.0208(14.56), 301.0699(20.67), 300.0288(12.19), 289.0697(53.9), 285.0749(12.64), 283.0229(21.43), 270.052(14.41); MS³[327.0473]: 311.0157(100.0), 299.0567(17.32), 283.0212(34.63)
**M183**	71.097	$C_{33}H_{26}O_{14}$	645.1252	Dimer of methyl taxiflolin and dimethyl quercetin 2	$[M + H]^+$: 647.1389	MS²[645.1285]: 329.0651(100.0), 327.0452(88.75), 317.0642(73.12), 315.0486(62.58), 301.0706(39.65), 300.0237(13.28), 289.0685(37.93), 285.0742(26.43), 257.0477(18.26); MS³[327.045]: 256.0334(100.0)
**M184**	62.067	$C_{33}H_{28}O_{14}$	647.1432	Dimer of methyl taxiflolin and dimethyl taxifolin 1	$[M + 2H_2O − H]^−$: 683.0739	MS²[647.1432]: 330.067(11.56), 329.0637(100.0), 317.0623(41.85), 301.0705(16.57), 289.0661(27.13), 285.0668(11.9), 273.0851(4.5), 270.0483(4.52), 231.0645(4.03)
**M185**	62.600	$C_{33}H_{28}O_{14}$	647.1420	Dimer of methyl taxiflolin and dimethyl taxifolin 2	$[M+H]^+$: 649.1402	MS²[647.1421]: 331.0632(1.39), 330.0683(12.06), 329.0651(100.0), 317.0634(54.54), 301.0681(31.1), 289.071(37.81), 285.0758(18.72), 270.0536(9.99); MS³[329.065]: 270.0485(100.0), 164.0181(85.56)

	t_R(min)	Formula	Meas. *m*/*z*	Identification	Other Ions	HRMS^n(Characteristic Fragment Ions and Their Relative Abundance)
**M186**	62.917	$C_{33}H_{28}O_{14}$	647.1419	Dimer of methyl taxiflolin and dimethyl taxifolin 3	$[M + H]^+$: 649.1544	MS^2[647.1419]: 330.0676(14.44), 329.0678(100.0), 317.0676(33.72), 301.0724(17.31), 289.0709(39.46), 285.0742(14.41), 273.0672(4.34), 271.0678(4.34), 269.0446(4.34), 231.0645(4.34)
**M187**	63.183	$C_{33}H_{28}O_{14}$	647.1406	Dimer of methyl taxiflolin and dimethyl taxifolin 4		MS^2[647.1368]: 330.0674(37.82), 329.0669(100.0), 317.0632(25.16), 311.0552(14.76), 303.0562(10.65), 301.067(23.06), 289.0673(46.05), 285.0422(8.55), 263.0667(8.55), 253.0461(14.76), 213.047(8.55)
**M188**	66.483	$C_{33}H_{28}O_{14}$	647.1434	Dimer of methyl taxiflolin and dimethyl taxifolin 5	$[M + H]^+$: 649.1516	MS^2[647.1434]: 330.0678(32.82), 329.0637(100.0), 317.061(25.6), 301.0701(22.99), 289.0707(32.3), 285.0753(14.74), 273.078(4.33), 270.0498(13.1); MS^3[329.0637]: 301.0888(24.67), 287.0443(13.46), 270.0519(46.67), 268.0359(44.51), 215.0699(26.74), 165.0081(13.46), 164.0127(100), 137.032(99.63)
**M189**	66.983	$C_{33}H_{28}O_{14}$	647.1405	Dimer of methyl taxiflolin and dimethyl taxifolin 6	$[M + H]^+$: 649.1557	MS^2[647.1406]: 330.0688(8.27), 329.0637(100.0), 317.0629(32.11), 301.0687(14.07), 289.0707(21.35), 285.0696(3.42), 274.0453(4.38), 269.0601(5.85), 268.0378(11.67)
**M190**	70.430	$C_{33}H_{28}O_{14}$	647.1421	Dimer of methyl taxiflolin and dimethyl taxifolin 7	$[M + H]^+$: 649.1720	MS^2[647.1424]: 332.0691(5.8), 331.0789(100.0), 319.0782(97.55), 317.0477(3.82), 313.0722(15.5), 301.0761(14.51), 300.0155(3.82), 211.0749(3.82)
**M191**	63.958	$C_{32}H_{26}O_{16}S$	697.0891	Dimer of methyl taxiflolin and dehydroxylated methyl taxifolin sulphate	$[M + H]^+$: 699.1071	MS^2[697.0789]: 617.1241(100.0), 329.0639(24.77), 317.0706(10.6), 313.0455(1.29), 299.0553(22.41), 297.0459(6.25); MS^3[617.1243]: 329.0645(100.0), 317.0666(76.67), 301.0744(15.27), 299.054(74.09), 289.0637(28.07), 285.0796(15.37)

Abbreviations: N means that the fragment ions of a metabolite are not obtained, and its corresponding extracted ion chromatograms in negative ion mode is shown in Figure **S4–S11**.

Table S2. Bioactivities of taxifolin metabolites.

No.	Identification	B1	B2	B3	B4	B5	B6	B7	B8	B9	B10	B11	B12
**M0**	Taxifolin(parent compound)	▲ [1]	▲ [2,3]	▲ [4]	▲ [5,6]	▲ [7]	▲ [8]	▲ [2]	▲ [9]	▲ [10]	▲ [11]	▲ [12]	▲ [13,14]
**M1, M2**	Taxifolin isomer	▲ [15]	▲ [16]	▲ [16,17]			▲ [18]			▲ [19]			
**M70**	Quercetin	▲ [1]	▲ [20]	▲ [21]	▲ [5]	▲ [22]	▲ [8,18]	▲ [23,24]	▲ [24]	▲ [25]	▲ [26]	▲ [27]	▲ [14,28]
**M73**	Quercetin-4′-*O*-sulfate	▲ [29]											
**M74**	Quercetin-3′-*O*-sulfate	▲ [29]		▲ [30,31]					▲ [32]				
**M75**	Quercetin-3-*O*-sulfate	▲ [33]	▲ [34]								▲ [34]		
**M76**	Quercetin -glucuronide	▲ [29]	▲ [35]	▲ [30,31]	▲ [5]				▲ [32]			▲ [27]	▲ [36]
**M80**	Isorhamnetin	▲ [37]	▲ [20]	▲ [21]	▲ [5]		▲ [8]	▲ [38]	▲ [39]	▲ [25,40]		▲ [41]	▲ [14,28]
**M83**	Isorhamnetin-3-*O*-sulfate	▲ [29]											
**M85**	Isorhamnetin disulfate	▲ [33]				▲ [42]							
**M104**	Eriodictyol	▲ [1]	▲ [43]	▲ [44]	▲ [44,45]	▲ [46]	▲ [47]	▲ [23]	▲ [48]	▲ [49]		▲ [50]	▲ [51]
**M105**	Dihydrokaempferol	▲ [52]	▲ [53]	▲ [53]			▲ [54,55]					▲ [56]	
**M113**	Luteolin-7-*O*-glucuronide		▲ [57]			▲ [58]							
**M122**	3/4-hydroxyphenylpropionic acid					▲ [59]	▲ [60,61]						
**M123**	3/4-hydroxyphenylpropionic acid					▲ [59]	▲ [60,61]						
**M131**	Dihydroxyphenylacetic acid	▲ [29]	▲ [62]	▲ [63]		▲ [59]	▲ [64]					▲ [65]	
**M136**	Dihydrocaffeic acid	▲ [29]	▲ [62,66]			▲ [59]	▲ [60]	▲ [67]					
**Sum**		12	11	10	6	8	10	6	7	5	5	8	6

Abbreviations: B1, Antioxidant; B2, Anti-inflammatory; B3, Antitumor; B4, Cardioprotective; B5, Antidiabetic; B6, Antimicrobial; B7, Antiviral; B8, Hepatoprotective; B9, Prevention of Alzheimer disease; B10, Immunoregulatory; B11, Xanthine oxidase inhibitor; B12, Neuroprotective

Table S3. The reported targets of taxifolin and its bioactive metabolites.

Target Name	Parent and Metabolites
Nrf2	**TAX** [68], **M80** [39], **M70** [69], **M104** [70]
β-amyloid (Aβ) peptides	**TAX** [71], **M70** [71], **M76** [72]
PI3-K phosphoinositide 3-kinase (PI3K)	**TAX** [73], **M80** [74], **M70** [75]
MEK1	**M70** [76], **M80** [74]
EGF receptor (EGFR)	**TAX** [73], **M70** [77]
heme oxygenase-1 (HO-1),	**M70** [78], **M104** [70]
microRNA-155	**M70** [79], **M80** [79]
TBK1-kinase	**M70** [80], **M104** [80]

Table S4. Targets of quercetin (**M70**) as predicted by PharmMapper.

Rank	PDB ID	Fit Score	Target Name
2	1uzf	3.844	Angiotensin-converting enzyme
64	1j1b	2.984	Glycogen synthase kinase-3 beta
139	1fcm	2.915	Beta-lactamase
269	1c9c	2.897	Beta-secretase 1
172	1m4h	2.85	Aspartate aminotransferase

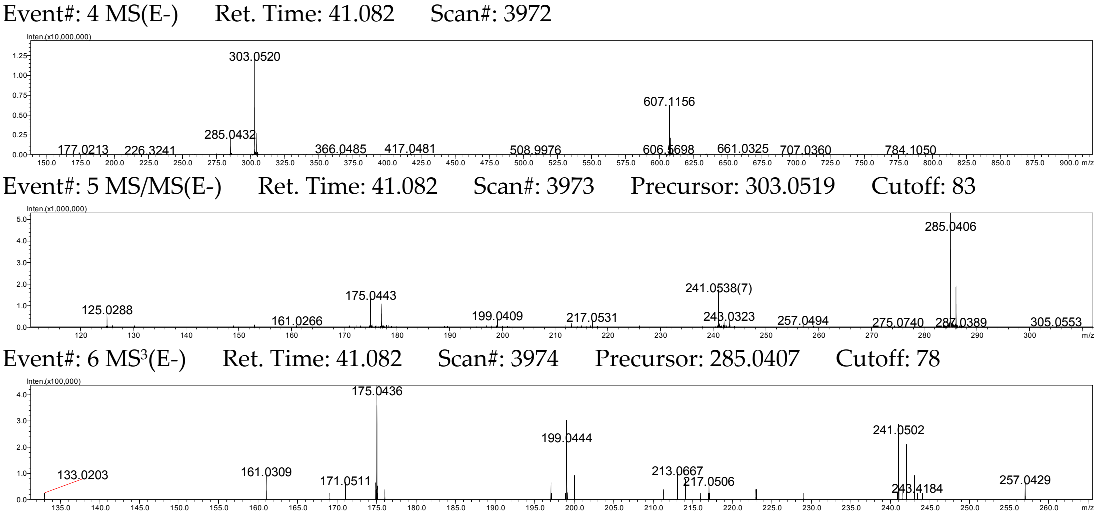

Figure. S1. Negative MS^n spectra of taxifolin.

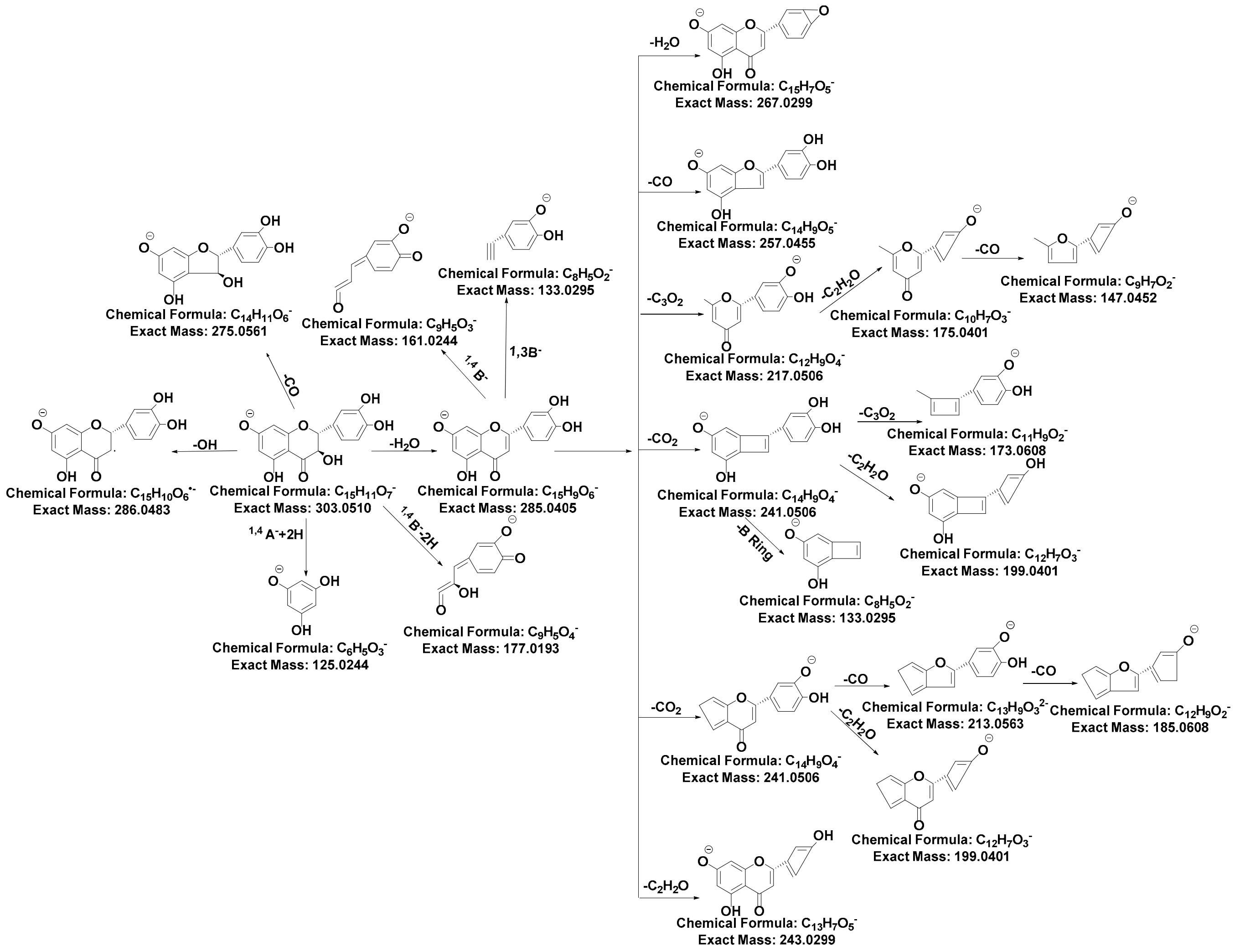

Figure S2. Proposed fragmentation pathways of taxifolin in negative ion detection mode mass spectra.

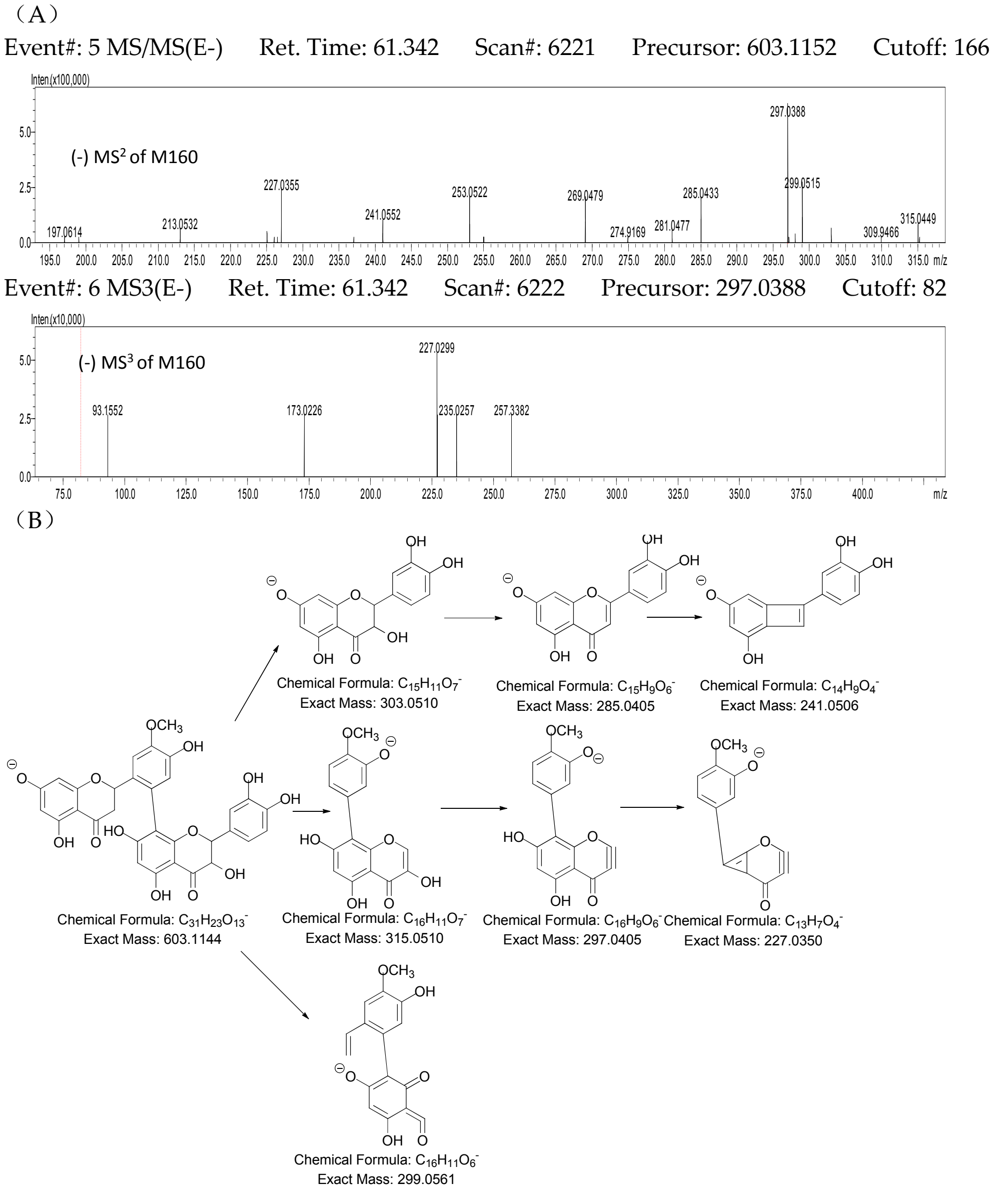

Figure S3. Proposed fragmentation pathways of **M160** in negative ion detection mode; (**A**) MS^n spectra of **M160** (**B**) fragmentation pathways, characteristic fragment ions, and proposed structure.

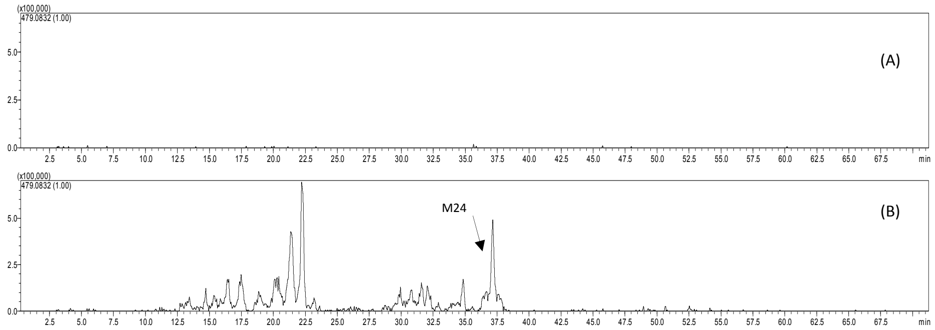

Figure S4. Extracted ion chromatograms at *m*/*z* 479.0832 in negative ion mode of urine samples: (**A**) blank group; (**B**) drug group.

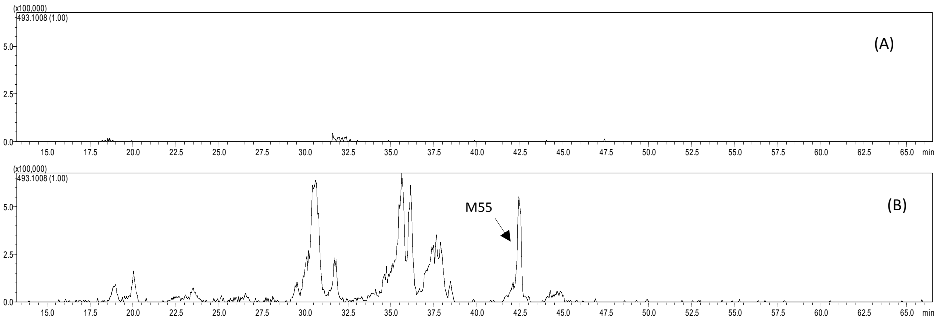

Figure S5. Extracted ion chromatograms at *m*/*z* 493.1008 in negative ion mode of urine samples: (**A**) blank group; (**B**) drug group.

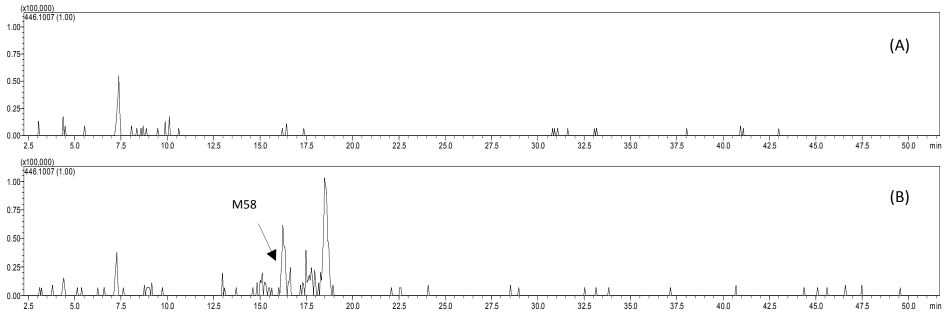

Figure S6. Extracted ion chromatograms at *m*/*z* 446.1007 in negative ion mode of urine samples: (**A**) blank group; (**B**) drug group.

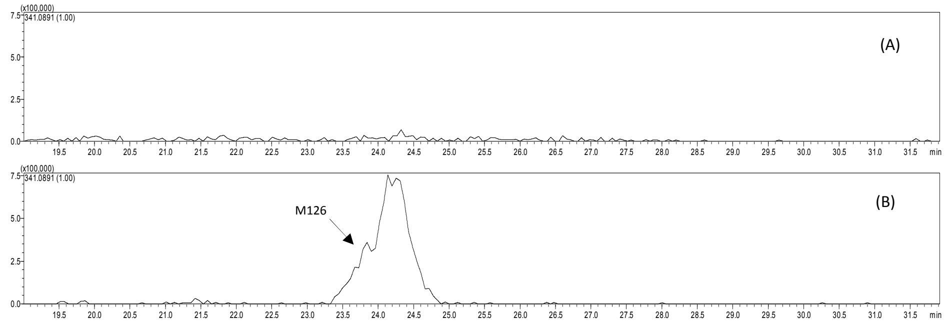

Figure S7. Extracted ion chromatograms at *m*/*z* 341.0891 in negative ion mode of urine samples: (**A**) blank group; (**B**) drug group.

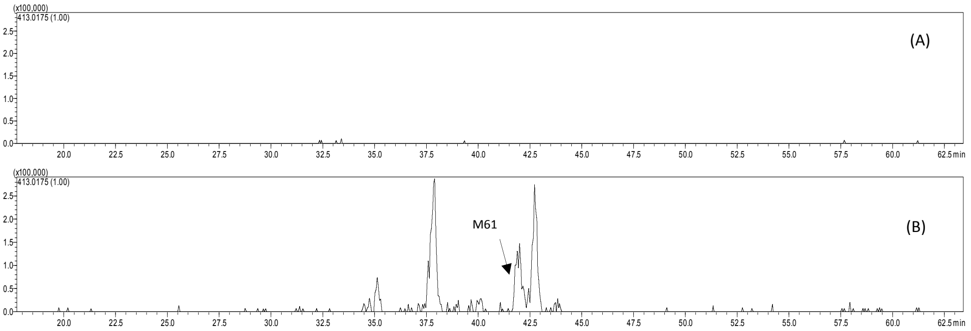

Figure S8. Extracted ion chromatograms at *m*/*z* 413.0175 in negative ion mode of plasma samples: (**A**) blank group; (**B**) drug group.

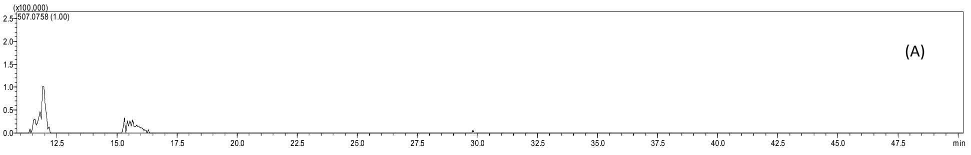

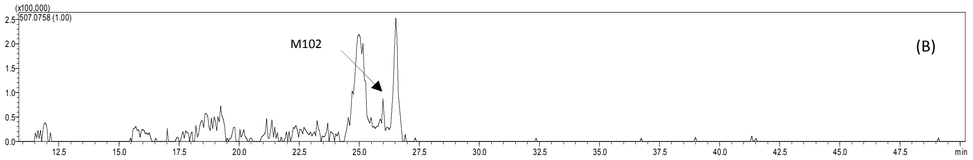

Figure S9. Extracted ion chromatograms at *m*/*z* 507.0758 in negative ion mode of plasma samples: (**A**) blank group; (**B**) drug group.

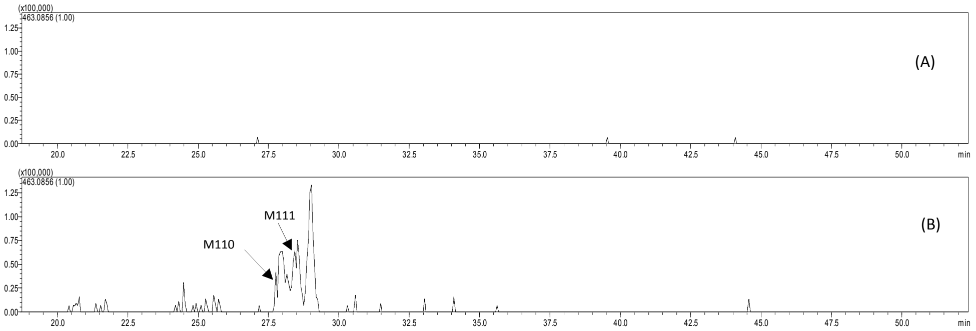

Figure S10. Extracted ion chromatograms at *m*/*z* 463.0856 in negative ion mode of plasma samples: (**A**) blank group; (**B**) drug group

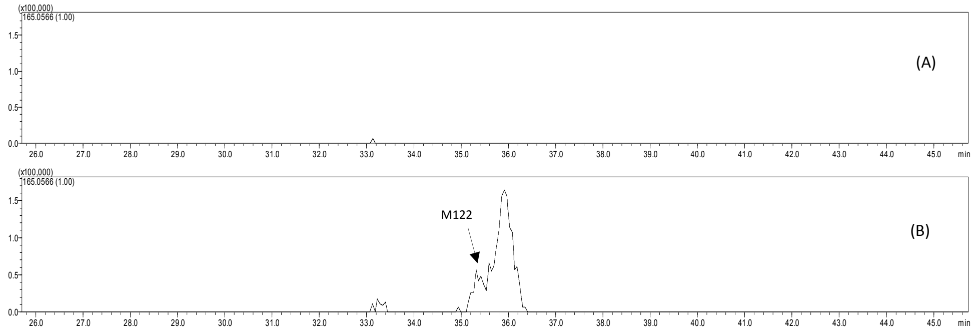

Figure S11. Extracted ion chromatograms at *m*/*z* 165.0566 in negative ion mode of faecal samples: (**A**) blank group; (**B**) drug group.

References

1. Sawai, Y. NMR analytical approach to clarify the molecular mechanisms of the antioxidative and radical-scavenging activities of antioxidants in tea: Reaction of polyphenols with stable free radicals. *Yasai Chagyo Kenkyusho Kenkyu Hokoku* **2007**, *6*, 23–58.
2. Yadava, R.N.; Raj, M. A new antiviral flavone glycoside from vernonia cinerea less. *Asian J. Chem.* **2013**, *25*, 3542–3544.
3. Delporte, C.; Backhouse, N.; Erazo, S.; Negrete, R.; Vidal, P.; Silva, X.; López-Pérez, J.L.; Feliciano, A.S.; Muñoz, O. Analgesic-antiinflammatory properties of proustia pyrifolia. *J. Ethnopharmacol.* **2005**, *99*, 119–124.
4. Horn, A.H.C. Letter to the editor: Quercetin and taxifolin completely break MDM2-p53 association: Molecular dynamics simulation study by s. Verma, a. Singh, a. Mishra. *Med. Chem. Res.* **2014**, *24*, 291–294.
5. Loke, W.M.; Proudfoot, J.M.; Mckinley, A.J.; Needs, P.W.; Kroon, P.A.; Hodgson, J.M.; Croft, K.D. Quercetin and its in vivo metabolites inhibit neutrophil-mediated low-density lipoprotein oxidation. *J. Agric. Food. Chem.* **2008**, *56*, 3609–3615.
6. Guo, H.; Zhang, X.; Cui, Y.; Zhou, H.; Xu, D.; Shan, T.; Zhang, F.; Guo, Y.; Chen, Y.; Wu, D. Taxifolin protects against cardiac hypertrophy and fibrosis during biomechanical stress of pressure overload. *Toxicol. Appl. Pharmacol.* **2015**, *287*, 168–177.
7. Fedosova, N.F.; Alisievich, S.V.; Lyadov, K.V.; Romanova, E.P.; Rud'Ko, I.A.; Kubatiev, A.A. Mechanisms underlying diquertin-mediated regulation of neutrophil function in patients with non-insulin-dependent diabetes mellitus. *Bull. Exp. Biol. Med.***2004**, *137*, 143–146.

8. Bunghez, F.; Socaciu, C.; Zagrean, F.; Pop, R.M.; Ranga, F.; Romanciuc, F. Characterisation of an aromatic plant-based formula using UV-Vis spectroscopy, LC-ESI(+)QTOF-MS and HPLC-DAD analysis. *Food Sci. Technol.* **2013**, *70*, 16–24.
9. Teselkin, Y.O.; Babenkova, I.V.; Kolhir, V.K.; Baginskaya, A.I.; Tjukavkina, N.A.; Kolesnik, Y.A.; Selivanova, I.A.; Eichholz, A.A. Dihydroquercetin as a means of antioxidative defence in rats with tetrachloromethane hepatitis. *Phytother. Res.* **2000**, *14*, 160–162.
10. Gocer, H.; Topal, F.; Topal, M.; Kucuk, M.; Teke, D.; Gulcin, I.; Alwasel, S.H.; Supuran, C.T. Acetylcholinesterase and carbonic anhydrase isoenzymes i and ii inhibition profiles of taxifolin. *J. Enzyme Inhib. Med. Chem.* **2015**, 1–7.
11. Ahn, J.Y.; Choi, S.E.; Jeung, M.S.; Park, K.H.; Moon, N.J.; Joo, S.S.; Lee, C.S.; Choi, Y.W.; Li, K.; Lee, M.-K.; et al. Effect of taxifolin glycoside on atopic dermatitis-like skin lesions in NC/Nga mice. *Phytother. Res.* **2010**, *24*, 1071–1077.
12. Van Norren, K.; Van Hoorn, E.C.; Leuvenink, H.G.D.; Hofman, Z. Compositions suitable for the treatment of damage caused by ischemia/reperfusion or oxidative stress. European Patent EP20010915929, 28 June 2006.
13. Wang, Q.Y.; Gao, X.; Wang, X.L.; Zhang, L.Y. Neuroprotective effects and mechanism of taxifolin against neural damage induced by aβ. *Zhongguo Shiyan Fangjixue Zazhi* **2014**, *20*, 164–167.
14. Dok-Go, H.; Lee, K.H.; Kim, H.J.; Lee, E.H.; Lee, J.; Song, Y.S.; Lee, Y.-H.; Jin, C.; Lee, Y.S.; Cho, J. Neuroprotective effects of antioxidative flavonoids, quercetin, (+)-dihydroquercetin and quercetin 3-methyl ether, isolated from opuntia ficus-indica var. Saboten. *Brain Res.* **2003**, *965*, 130–136.
15. Jiang, W.-J.; Ishiuchi, K.-i.; Furukawa, M.; Takamiya, T.; Kitanaka, S.; Iijima, H. Stereospecific b inhibition of nitric oxide production in macrophage cells by flavanonols: Synthesis and the structure-activity relationship. *Bioorg. Med. Chem.* **2015**, *23*, 6922–6929.
16. Kim, K.H.; Moon, E.; Choi, S.U.; Pang, C.; Kim, S.Y.; Lee, K.R. Identification of cytotoxic and anti-inflammatory constituents from the bark of toxicodendron vernicifluum (stokes) f.A. Barkley. *J. Ethnopharmacol.* **2015**, *162*, 231–237.
17. Wang, Y.-F.; Cao, J.-X.; Efferth, T.; Lai, G.-F.; Luo, S.-D. Cytotoxic and new tetralone derivatives from berchemia floribunda (wall.) brongn. *Chem. Biodivers.* **2006**, *3*, 646–653.
18. Nandan, D.; Lopez, M.; Ban, F.; Huang, M.; Li, Y.; Reiner, N.E.; Cherkasov, A. Indel-based targeting of essential proteins in human pathogens that have close host ortholog(s): Discovery of selective inhibitors for leishmania donovani elongation factor-1α. *Proteins: Struct. Funct. Bioinf.* **2007**, *67*, 53–64.
19. Sato, M.; Murakami, K.; Uno, M.; Ikubo, H.; Nakagawa, Y.; Katayama, S.; Akagi, K.; Irie, K. Structure-activity relationship for (+)-taxifolin isolated from silymarin as an inhibitor of amyloid beta aggregation. *Biosci. Biotechnol. Biochem.* **2013**, *77*, 1100–1103.
20. Boesch-Saadatmandi, C.; Loboda, A.; Wagner, A.E.; Stachurska, A.; Jozkowicz, A.; Dulak, J.; Doring, F.; Wolffram, S.; Rimbach, G. Effect of quercetin and its metabolites isorhamnetin and quercetin-3-glucuronide on inflammatory gene expression: Role of MIR-155. *J. Nutr. Biochem.* **2011**, *22*, 293–299.
21. Delgado, L.; Fernandes, I.; Gonzalez-Manzano, S.; de Freitas, V.; Mateus, N.; Santos-Buelga, C. Anti-proliferative effects of quercetin and catechin metabolites. *Food Funct* **2014**, *5*, 797–803.
22. Boonpawa, R.; Spenkelink, A.; Rietjens, I.M.C.M.; Punt, A. A physiologically based kinetic (pbk) model describing plasma concentrations of quercetin and its metabolites in rats. *Biochem. Pharmacol.* **2014**, *89*, 287–299.
23. Li, Y.; Wang, H.; Fan, Z.; Su, M.; Xue, J.; Li, T.; Cen, Y. Flavonoids from adina pilulifera and their in vitro antiviral activity. *Tianran Chanwu Yanjiu Yu Kaifa* **2009**, *21*, 740–743.
24. Kumar, S.; Pandey, A.K. Chemistry and biological activities of flavonoids: An overview. *Scientific World J.* **2013**, *2013*, 162750.
25. Li, C.; Hu, L.; Shen, X.; Yang, Z.; Jiang, H. Extraction of β-secretase inhibitor flavonol compound from gingko leaf and its application for treatment of alzheimer's disease. Chinese Patent, Application No.: CN200610028826.4, 11 July 2006.
26. Sternberg, Z.; Chadha, K.; Lieberman, A.; Hojnacki, D.; Drake, A.; Zamboni, P.; Rocco, P.; Grazioli, E.; Weinstock-Guttman, B.; Munschauer, F. Quercetin and interferon-β modulate immune response(s) in peripheral blood mononuclear cells isolated from multiple sclerosis patients. *J. Neuroimmun.* **2008**, *205*, 142–147.
27. Day, A.J.; Bao, Y.; Morgan, M.R.A.; Williamson, G. Conjugation position of quercetin glucuronides and

effect on biological activity. *Free Radic. Biol. Med.* **2000**, *29*, 1234–1243.
28. Karjalainen, R.; Anttonen, M.; Saviranta, N.; Hilz, H.; Torronen, R.; Stewart, D.; McDougall, G.J.; Mattila, P. A review on bioactive compounds in black currants (Ribes nigrum L.) and their potential health-promoting properties. *Acta Horticulturae* **2009**, *839*, 301–307.
29. Duenas, M.; Surco-Laos, F.; Gonzalez-Manzano, S.; Gonzalez-Paramas, A.M.; Santos-Buelga, C. Antioxidant properties of major metabolites of quercetin. *Eur. Food Res. Technol.* **2011**, *232*, 103–111.
30. Donnini, S.; Finetti, F.; Lusini, L.; Morbidelli, L.; Cheynier, V.; Barron, D.; Williamson, G.; Waltenberger, J.; Ziche, M. Divergent effects of quercetin conjugates on angiogenesis. *Br. J. Nutr.* **2006**, *95*, 1016–1023.
31. Yang, J.-H.; Hsia, T.-C.; Kuo, H.-M.; Chao, P.-D.L.; Chou, C.-C.; Wei, Y.-H.; Chung, J.-G. Inhibition of lung cancer cell growth by quercetin glucuronides via G2/M arrest and induction of apoptosis. *Drug Metab. Dispos.* **2006**, *34*, 296–304.
32. Yokoyama, A.; Sakakibara, H.; Crozier, A.; Kawai, Y.; Matsui, A.; Terao, J.; Kumazawa, S.; Shimoi, K. Quercetin metabolites and protection against peroxynitrite-induced oxidative hepatic injury in rats. *Free Radical Res.* **2009**, *43*, 913–921.
33. Yagi, A.; Uemura, T.; Okamura, N.; Haraguchi, H.; Imoto, T.; Hashimoto, K. Antioxidative sulfated flavonoids in leaves of polygonum hydropiper. *Phytochemistry* **1994**, *35*, 885–887.
34. Ksouri, R.; Ksouri, W.M.; Jallali, I.; Debez, A.; Magne, C.; Hiroko, I.; Abdelly, C. Medicinal halophytes: Potent source of health promoting biomolecules with medical, nutraceutical and food applications. *Crit. Rev. Biotechnol.* **2012**, *32*, 289–326.
35. Derlindati, E.; Dall'Asta, M.; Ardigo, D.; Brighenti, F.; Zavaroni, I.; Crozier, A.; Del Rio, D. Quercetin-3-*O*-glucuronide affects the gene expression profile of M1 and M2a human macrophages exhibiting anti-inflammatory effects. *Food Funct.* **2012**, *3*, 1144–1152.
36. Faria, A.; Meireles, M.; Fernandes, I.; Santos-Buelga, C.; Gonzalez-Manzano, S.; Duenas, M.; de Freitas, V.; Mateus, N.; Calhau, C. Flavonoid metabolites transport across a human BBB model. *Food Chem.* **2014**, *149*, 190–196.
37. Seo, K.; Yang, J.H.; Kim, S.C.; Ku, S.K.; Ki, S.H.; Shin, S.M. The antioxidant effects of isorhamnetin contribute to inhibit COX-2 expression in response to inflammation: A potential role of HO-1. *Inflammation* **2014**, *37*, 712–722.
38. Du, L.-n.; Shan, J.-j.; Xu, J.-y.; Dai, Q.-g.; Wang, S.-c. In vitro experimental study of anti-respiratory syncytial virus effect of jinxin oral liquid transitional ingredients in blood. *Zhonghua Zhongyiyao Zazhi* **2013**, *28*, 955–958.
39. Yang, J.H.; Shin, B.Y.; Han, J.Y.; Kim, M.G.; Wi, J.E.; Kim, Y.W.; Cho, I.J.; Kim, S.C.; Shin, S.M.; Ki, S.H. Isorhamnetin protects against oxidative stress by activating nrf2 and inducing the expression of its target genes. *Toxicol. Appl. Pharmacol.* **2014**, *274*, 293–301.
40. Xu, S.L.; Zhu, K.Y.; Bi, C.W.C.; Choi, R.C.Y.; Miernisha, A.; Yan, A.L.; Maiwulanjiang, M.; Men, S.W.X.; Dong, T.T.X.; Tsim, K.W.K. Flavonoids induce the expression of synaptic proteins, synaptotagmin, and postsynaptic density protein-95 in cultured rat cortical neuron. *Planta Med.* **2013**, *79*, 1710–1714.
41. Liu, Z.; Liu, S.; Xing, J.; Song, F.; Zheng, C.; Liu, S. Method for screening xanthine oxidase inhibitor by ultrahigh-performance liquid chromatography-mass spectrometry. Chinese Patent, Application No.: CN201010577534.2, 8 December 2010.
42. Haraguchi, H.; Ohmi, I.; Sakai, S.; Fukuda, A.; Toihara, Y.; Fujimoto, T.; Okamura, N.; Yagi, A. Effect of polygonum hydropiper sulfated flavonoids on lens aldose reductase and related enzymes. *J. Nat. Prod.* **1996**, *59*, 443–445.
43. Armah, F.A.; Annan, K.; Mensah, A.Y.; Amponsah, I.K.; Tocher, D.A.; Habtemariam, S. Erythroivorensin: A novel anti-inflammatory diterpene from the root-bark of erythrophleum ivorense (a chev.). *Fitoterapia* **2015**, *105*, 37–42.
44. Lee, S.E.; Yang, H.; Son, G.W.; Park, H.R.; Park, C.-S.; Park, Y.S.; Jin, Y.-H. Eriodictyol protects endothelial cells against oxidative stress-induced cell death through modulating ERK/Nrf2/are-dependent heme oxygenase-1 expression. *Int. J. Mol. Sci.* **2015**, *16*, 14526–14539.
45. Wang, C.; Zhang, Y.F.; Zhao, X.P. Study on screening cardioprotective components from zhishi xiebai guizhi tang. *Zhongguo Zhong Yao Za Zhi* **2013**, *38*, 1601–1605.
46 Zhang, W.Y.; Lee, J.J.; Kim, Y.; Kim, I.S.; Han, J.H.; Lee, S.G.; Ahn, M.J.; Jung, S.H.; Myung, C.S. Effect of eriodictyol on glucose uptake and insulin resistance in vitro. *J. Agric. Food Chem.* **2012**, *60*, 7652–7658.
47. Habtemariam, S.; Dagne, E. Comparative antioxidant, prooxidant and cytotoxic activity of sigmoidin A and

eriodictyol. *Planta Med.* **2010**, *76*, 589–594.

48. Ferreira, P.S.; Nasser, A.L.M.; Goncalves, D.; Spolidorio, L.C.; Manthey, J.A.; Cesar, T.B. In Citrus Flavonones Decreases Oxidative Stress in the Liver and Blood Serum Caused by High-Fat Diet Feeding in c57bl/6j Mice, ACS National Meeting & Exposition, Boston, MA, USA, 16–20 August 2015.
49. Jing, X.; Shi, H.; Zhu, X.; Wei, X.; Ren, M.; Han, M.; Ren, D.; Lou, H. Eriodictyol attenuates β-amyloid 25-35 peptide-induced oxidative cell death in primary cultured neurons by activation of Nrf2. *Neurochem. Res.* **2015**, *40*, 1463–1471.
50. Nguyen, M.T.T.; Awale, S.; Tezuka, Y.; le Tran, Q.; Watanabe, H.; Kadota, S. Xanthine oxidase inhibitory activity of vietnamese medicinal plants. *Biol. Pharm.Bull.* **2004**, *27*, 1414–1421.
51. Cho, N.; Choi, J.H.; Yang, H.; Jeong, E.J.; Lee, K.Y.; Kim, Y.C.; Sung, S.H. Neuroprotective and anti-inflammatory effects of flavonoids isolated from rhus verniciflua in neuronal HT22 and microglial BV2 cell lines. *Food Chem. Toxicol.* **2012**, *50*, 1940–1945.
52. Lee, I.K.; Song, K.S.; Kim, C.J.; Kim, H.M.; Oh, G.T.; Yoo, I.D. Tumor cell growth inhibition and antioxidant activity of flavonoids from the stem bark of cudrania tricuspidata. *Han'guk Nonghwa Hakhoechi* **1994**, *37*, 105–109.
53. Tan, Y.; Liu, C.; Chen, R. Phenolic constituents from stem bark of morus wittiorum and their anti-inflammation and cytotoxicity. *Zhongguo Zhong Yao Za Zhi* **2010**, *35*, 2700–2703.
54. Ango, P.Y.; Kapche, D.W.F.G.; Kuete, V.; Ngadjui, B.T.; Bezabih, M.; Abegaz, B.M. Chemical constituents of trilepisium madagascariense (moraceae) and their antimicrobial activity. *Phytochem. Lett.* **2012**, *5*, 524–528.
55. Tajuddeen, N.; Sallau, M.S.; Musa, A.M.; Habila, D.J.; Yahaya, S.M. Flavonoids with antimicrobial activity from the stem bark of commiphora pedunculata (kotschy & peyr.) engl. *Natural Prod. Res.* **2014**, *28*, 1915–1918.
56. Xu, W.A.; Yin, L.; Pan, H.Y.; Shi, L.; Xu, L.; Zhang, X.; Duan, J.A. Study on the correlation between constituents detected in serum from rhizoma smilacis glabrae and the reduction of uric acid levels in hyperuricemia. *J. Ethnopharmacol.* **2013**, *150*, 747–754.
57. Min, Y.S.; Bai, K.L.; Yim, S.H.; Lee, Y.J.; Song, H.J.; Kim, J.H.; Ham, I.; Whang, W.K.; Sohn, U.D. The effect of luteolin-7-*O*-β-d-glucuronopyranoside on gastritis and esophagitis in rats. *Arch. Pharmacal Res.* **2006**, *29*, 484–489.
58. Shimizu, M.; Ito, T.; Terashima, S.; Hayashi, T.; Arisawa, M.; Morita, N.; Kurokawa, S.; Ito, K.; Hashimoto, Y. Inhibition of lens aldose reductase by flavonoids. *Phytochemistry* **1984**, *23*, 1885–1888.
59. Fernandez-Millan, E.; Ramos, S.; Alvarez, C.; Bravo, L.; Goya, L.; Martin, M.A. Microbial phenolic metabolites improve glucose-stimulated insulin secretion and protect pancreatic beta cells against tert-butyl hydroperoxide-induced toxicity via ERKs and PKC pathways. *Food Chem. Toxicol.* **2014**, *66*, 245–253.
60. Kabbash, C.; Silverstein, S.C.; Shuman, H.A.; Blanchard, J.S. Antimicrobial activity of gemfibrozil and related compounds and derivatives and metabolites based on inhibition of enoyl-[acyl carrier protein] reductase. US Patent US1999-438144, 19 April 2005.
61. Cueva, C.; Moreno-Arribas, M.V.; Martin-Alvarez, P.J.; Bills, G.; Vicente, M.F.; Basilio, A.; Rivas, C.L.; Requena, T.; Rodriguez, J.M.; Bartolome, B. Antimicrobial activity of phenolic acids against commensal, probiotic and pathogenic bacteria. *Res. Microbiol.* **2010**, *161*, 372–382.
62. Monagas, M.; Khan, N.; Andres-Lacueva, C.; Urpi-Sarda, M.; Vazquez-Agell, M.; Lamuela-Raventos, R.M.; Estruch, R. Dihydroxylated phenolic acids derived from microbial metabolism reduce lipopolysaccharide-stimulated cytokine secretion by human peripheral blood mononuclear cells. *Br. J. Nutr.* **2009**, *102*, 201–206.
63. Gao, K.; Xu, A.; Krul, C.; Venema, K.; Liu, Y.; Niu, Y.; Lu, J.; Bensoussan, L.; Seeram, N.P.; Heber, D.; et al. Of the major phenolic acids formed during human microbial fermentation of tea, citrus, and soy flavonoid supplements, only 3,4-dihydroxyphenylacetic acid has antiproliferative activity. *J. Nutrition* **2006**, *136*, 52–57.
64. Alakomi, H.L.; Puupponen-Pimiae, R.; Aura, A.M.; Helander, I.M.; Nohynek, L.; Oksman-Caldentey, K.M.; Saarela, M. Weakening of salmonella with selected microbial metabolites of berry-derived phenolic compounds and organic acids. *J. Agric. Food. Chem.* **2007**, *55*, 3905–3912.
65. Kubo, I.; Masuoka, N.; Nihei, K.I.; Burgheim, B. Manicoba, a quercetin-rich amazonian dish. *J. Food Compos. Anal.* **2006**, *19*, 579–588.
66. Yang, X.W.; Wang, N.; Li, W.; Xu, W.; Wu, S. Biotransformation of 4,5-*O*-dicaffeoylquinic acid methyl ester by human intestinal flora and evaluation on their inhibition of no production and antioxidant activity of the products. *Food Chem. Toxicol.* **2013**, *55*, 297–303.

67. Helbig, B.; Thiel, K.D. Comparison of the antiviral activity of oxidized caffeic acid and hydrocaffeic acid against herpesvirus hominis types 1 and 2 in vitro. *Die Pharmazie* **1982**, *37*, 603–604.
68. Samatiwat, P.; Prawan, A.; Senggunprai, L.; Kukongviriyapan, V. Taxifolin exerts cytoprotective effect by activation of Nrf2-are signaling pathway in HepG2 cells. *Srinagarind Med. J.* **2014**, 29, 122–125.
69. Granado-Serrano, A.B.; Martín, M.A.; Bravo, L.; Goya, L.; Ramos, S. Quercetin modulates NRF2 and glutathione-related defenses in HepG2 cells: Involvement of p38. *Chem. Biol. Interact.* **2012**, *195*, 154–164.
70. Lee, S.E.; Yang, H.; Son, G.W.; Park, H.R.; Park, C.S.; Jin, Y.H.; Park, Y.S. Eriodictyol protects endothelial cells against oxidative stress-induced cell death through modulating ERK/Nrf2/ARE-dependent heme oxygenase-1 expression. *Int. J. Mol. Sci.* **2015**, *16*, 14526–14539.
71. Hanaki, M.; Murakami, K.; Akagi, K.I.; Irie, K. Structural insights into mechanisms for inhibiting amyloid β42 aggregation by non-catechol-type flavonoids. *Bioorg. Med. Chem.* **2016**, *24*, 304–313.
72. Ho, L.; Ferruzzi, M.G.; Janle, E.M.; Wang, J.; Gong, B.; Chen, T.Y.; Lobo, J.; Cooper, B.; Wu, Q.L.; Talcott, S.T. Identification of brain-targeted bioactive dietary quercetin-3-*O*-glucuronide as a novel intervention for Alzheimer's disease. *FASEB. J.* **2013**, *27*, 769–781.
73. Oi, N.; Chen, H.; Kim, M.O.; Lubet, R.A.; Bode, A.M.; Dong, Z. Taxifolin suppresses UV-induced skin carcinogenesis by targeting EGFR and PI3K. *Cancer Prev. Res.***2012**, *5*, 1103–1114.
74. Kim, J.E.; Lee, D.E.; Lee, K.W.; Son, J.E.; Seo, S.K.; Li, J.; Jung, S.K.; Heo, Y.S.; Mottamal, M.; Bode, A.M. Isorhamnetin suppresses skin cancer through direct inhibition of mek1 and PI3-K. *Cancer Prev. Res.* **2011**, *4*, 582–591.
75. Yoshizumi, M.A.M.T.U.; Tsuchiya, K.; Kirima, K.; Kyaw, M.; Suzaki, Y.; Tamaki, T. Quercetin inhibits Shc- and phosphatidylinositol 3-kinase-mediated c-Jun N-terminal kinase activation by angiotensin ii in cultured rat aortic smooth muscle cells. *Mol. Pharmacol.* **2001**, *60*, 656–665.
76. Nguyen, T.T.T.; Tran, E.; Nguyen, T.H.; Do, P.T.; Huynh, T.H.; Huynh, H. The role of activated MEK-ERK pathway in quercetin. *Carcinogenesis* **2004**, *25*, 647–659.
77. Fridrich, D.; Teller, N.; Esselen, M.; Pahlke, G.; Marko, D. Comparison of delphinidin, quercetin and (−)-epigallocatechin-3-gallate as inhibitors of the EGFR and the ErbB2 receptor phosphorylation. *Mol. Nutr. Food Res.* **2008**, *52*, 815–822
78. Rayamajhi, N.; Kim, S.K.; Go, H.; Joe, Y.; Callaway, Z.; Kang, J.G.; Ryter, S.W.; Chung, H.T. Quercetin induces mitochondrial biogenesis through activation of HO-1 in HepG2 cells. *Oxid. Med. Cell. Longev.* **2013**, *2013*, 450–450.
79. Boesch-Saadatmandi, C.; Loboda, A.; Wagner, A.E.; Stachurska, A.; Jozkowicz, A.; Dulak, J.; Döring, F.; Wolffram, S.; Rimbach, G. Effect of quercetin and its metabolites isorhamnetin and quercetin-3-glucuronide on inflammatory gene expression: Role of miR-155 . *J. Nutr. Biochem.* **2011**, *22*, 293–299.
80. Lee, J.K.; Kim, S.Y.; Kim, Y.S.; Lee, W.H.; Hwang, D.H.; Lee, J.Y. Suppression of the TRIF-dependent signaling pathway of toll-like receptors by luteolin. *Biochem. Pharmacol.* **2009**, *77*, 1391–1400.
